# ATP synthase is a promising target for identifying activated and non-activated adipose tissues

**DOI:** 10.1038/s41467-026-71343-w

**Published:** 2026-04-15

**Authors:** Caitlin V.M.L. Jie, Aro Delparente, Tongtong Wang, Lisa Reichert, Petra Krajnovic, Marc Schläppi, Lukas Reininger, Laura T. L. Brandt, Claudia Keller, Julien Orts, Roland Riek, Stefanie D. Krämer, Markus Stoffel, Christian Wolfrum, Roger Schibli, Linjing Mu

**Affiliations:** 1 Department of Chemistry and Applied Biosciences, Institute of Pharmaceutical Sciences, ETH Zürich, Zürich, Switzerland; 2 Department of Health Sciences and Technology, Laboratory of Translational Nutrition Biology, ETH Zürich, Schwerzenbach, Switzerland; 3 Department of Biology, Institute for Molecular Health Sciences, ETH Zürich, Zürich, Switzerland; 4 Department of Chemistry and Applied Biosciences, Institute for Molecular Physical Sciences, ETH Zürich, Zürich, Switzerland; 5 Department of Pharmaceutical Sciences, University of Vienna, Vienna, Austria; 6 Center for Radiopharmaceutical Sciences, PSI Center for Life Sciences, Villigen-PSI, Switzerland

**Keywords:** Positron-emission tomography, Predictive markers

## Abstract

Adipose tissue has gained increasing attention as a therapeutic target to combat human obesity and related metabolic disorders. We propose ATP synthase as a target to identify activated and non-activated adipose tissue. We investigated ATP synthase using the radiotracer [^11^C]J147 and confirmed the specificity of the radiotracer by in vitro autoradiography, cell knockdown studies, and in vivo competition binding studies. In addition to the interscapular brown adipose tissue (BAT), [^11^C]J147 could visualise several other BAT depots (supraspinal, infrascapular and axillary BAT) via in vivo positron emission tomography imaging after activation with a β_3_-adrenergic receptor agonist, as confirmed by immunohistochemistry and biodistribution studies. Furthermore, [^11^C]J147 demonstrated higher sensitivity for BAT and WAT (white adipose tissue) identification compared to the commonly used radiotracer [^18^F]FDG and the mitochondrial complex I tracer [^18^F]BCPP-EF. Our study uncovers ATP synthase as a promising target for monitoring adipose tissue and [^11^C]J147 could facilitate drug development for metabolic diseases such as obesity.

## Abstract (web summary)

This study explored ATP synthase as a target for PET imaging of activated and nonactivated adipose tissue using the radiotracer [¹¹C]J147, providing a potential tool to support drug development for metabolic diseases such as obesity.

## Introduction

Adipose tissue plays a central role in metabolism and energy homoeostasis. The two main types of adipose tissues are the brown adipose tissue (BAT) and white adipose tissue (WAT)^1^. The discovery of BAT in adult humans has highlighted the potential of targeting cellular bioenergetics to dissipate excess energy, offering a promising therapeutic approach for obesity and related diseases^2^. Developing BAT-targeted obesity treatments requires tools to directly measure, quantify, and monitor the thermogenic adipocytes in vivo as it is essential for patient selection, treatment monitoring, and accelerating drug development.

Positron emission tomography (PET) is a translational molecular imaging modality with exceptional sensitivity. Several PET tracers have been evaluated for their effectiveness in quantifying BAT. [^18^F]Fluorodeoxyglucose ([^18^F]FDG), a glucose analogue, has confirmed that metabolically activated BAT exists in the adult population within the neck and supraclavicular areas^3–5^. A variety of other radiotracers have been developed to investigate different aspects of tissue metabolism and mitochondrial function. These include metabolic tracers such as [^18^F]THA and [^11^C]acetate^6^; adrenergic receptors tracers such as [^123^I]metaiodobenzylguanidine and [^18^F]F-DA^7,8^; translocator protein tracers ([^18^F]fmPBR28-*d*_2_ and [^18^F]FEPPA); mitochondrial membrane potential-targeting tracers ([^18^F]FBnTP and [^18^F]BODIPY1)^9–11^, and a fatty acid tracer ([^123/125^I]-β-Methyl-p-iodophenyl-pentadecanoic acid [^123/125^I-BMIPP])^12^. Despite these advances, the accurate and specific imaging of adipose tissue remains challenging. Whilst tracers such as [^18^F]fmPBR28-*d*_2_ and [^18^F]BODIPY1 could detect interscapular BAT in thermoneutral and cold-exposed conditions^13,14^, others like [^18^F]FEPPA and [^18^F]BCPP-EF displayed no increased uptake in β_3_-adrenergic agonist-activated BAT, or even demonstrated decreased uptake after activation of adipose tissue via cold exposure in the case of [^18^F]FBnTP^10,15,16^. The current gold standard, [¹⁸F]FDG, targets glucose metabolism but has limited specificity in distinguishing increased tracer uptake arising from adipocyte metabolic activity versus neuroinflammatory processes^17–19^. Consequently, it cannot reliably or conclusively detect the presence or absence of BAT in some cases. These findings underscore the necessity of exploring novel targets and PET tracers to enable more reliable and accurate imaging of adipose tissue.

The mitochondria uncoupling protein 1 (UCP1) has been identified as the principal mediator of thermogenesis, particularly within brite adipocytes and BAT, where it orchestrates heat production through its unique ability to uncouple oxidative phosphorylation from ATP synthase^20–22^. Beyond the well-established upregulation of UCP1 following BAT activation (e.g. cold stimulation), increases in BAT mass and ATP synthase content have been reported^23^. Several studies have further demonstrated that UCP1 increase is accompanied by the upregulation of mitochondrial genes and proteins involved in the respiratory chain, including ATP synthase as well as markers of mitochondrial biogenesis^23–27^. Consistent with these results, ATP synthase upregulation has also been reported in UCP1-recombinant yeast cells, with a 3.6-fold increase observed in strains exhibiting high UCP1 expression^28^. These findings support a positive association between BAT activation, UCP1 expression and increased ATP synthase abundance.

In recent years, researchers have increasingly recognised the importance of ATP-dependent futile cycles, including lipid, calcium, and creatine cycles^29–32^. These futile cycles depend on ATP to produce the relevant product but result in an immediate conversion back to the substrate, resulting in zero net change in metabolite concentrations and dissipation of energy as heat. Due to the central role of ATP synthase in energy homoeostasis in BAT, we explored it as a potential biomarker for imaging activated and non-activated adipose tissue. The curcumin-derivative J147 was discovered via a phenotypic screening for the selection of neuroprotective compounds^33,34^. The neuroprotective effect of J147 was investigated in several rodent models, including models for Alzheimer’s disease^35^, aging^36^ and fatty liver disease^37^. J147 targets the alpha subunit of ATP synthase (ATP5A) and inhibits at low nanomolar concentrations, as shown in isolated bovine heart mitochondria^34^. As previous curcumin-based probes have demonstrated to be selective fluorescence imaging probes for BAT and allow monitoring of the browning of BAT^38^, we explored ^11^C-labelled J147 ([^11^C]J147) as a potential PET tracer for monitoring and characterising adipose tissues by targeting the ATP synthase^39^. [^11^C]J147 has been reported shortly after the discovery of J147 however, no further in vitro and in vivo studies have been reported since.

In this study, we radiosynthesised [^11^C]J147 by adapting the previously published method and evaluated its specificity to ATP synthase in vitro by autoradiography with BAT and WAT tissue slices and uptake in ATP5A knockdown cells. In vivo PET imaging, biodistribution, and competition binding studies were performed in healthy and β_3_-adrenergic agonist CL316,243-treated mice. The in vitro and in vivo results with [^11^C]J147 were compared to immunohistochemistry images of ATP5A and UCP1. The gold standard [^18^F]FDG and mitochondrial complex I tracer [^18^F]BCPP-EF were included in our studies for comparison with [^11^C]J147. To further evaluate ATP synthase as a valuable imaging target in metabolic disease, we investigated its expression in adipose tissues in a mouse model of metabolic disease and studied the effect of liraglutide treatment on ATP synthase expression in healthy mice.

## Results

### [^11^C]J147 can monitor BAT and WAT activation and ATP5A expression in vitro

**Fig. 1:**
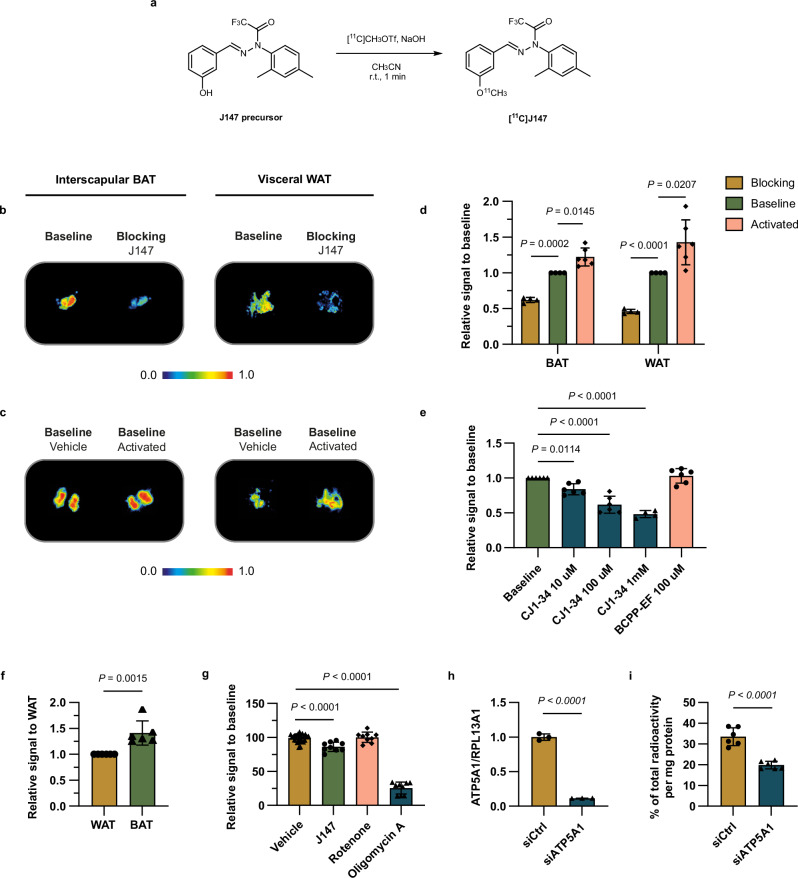
In vitro evaluation of [^11^C]J147. **a** Radiolabelling procedure of [^11^C]J147. **b**, **c** In vitro autoradiography with [^11^C]J147 on tissue slices from interscapular BAT and visceral WAT, representative autoradiographs for each group. **b** Incubation with [^11^C]J147 (3–5 nM; baseline condition) or [^11^C]J147 (3–5 nM) together with 100 µM unlabelled J147 for competition (blocking condition). **c** Mice were treated with vehicle (control) or CL316,243 (activated), and tissue slices were incubated with [^11^C]J147 (3–5 nM). **d** Semiquantitative analysis of interscapular BAT and visceral WAT autographs following blockade (brown) or activation (pink) compared to baseline (green) (*n* = 6 for each). J147 blockade decreased signal in BAT and WAT (*p* = 0.0002; *p* < 0.0001), whereas signal increased in both activated tissues respectively (*p* = 0.0145; *p* = 0.0207). **e** Semiquantification of autoradiographs from interscapular BAT slices of CL316,243-treated mice incubated with [¹¹C]J147 (*n* = 6, 3–5 nM; baseline condition, green) and co-incubated with indicated concentrations of CJ1-34 (10 µM, *n* = 6, *p* = 0.0114; 100 µM, *n* = 6, *p* < 0.0001 and 1 mM, *n* = 4, *p* < 0.0001, blue) or BCPP-EF (100 µM, *n* = 6, pink, served as a negative control). **f** Semiquantifcation of autoradiographs of WAT (brown) vs BAT (green) (non-activated animals, same data as in (**b**, **d**) (*n* = 6). **g** ATP hydrolysis assay showing significant effect by J147 treatment (*n* = 9, *p* < 0.0001, green) and oligomycin A treatment (*n* = 9, *p* < 0.0001 blue) but no effect by rotenone (*p*ink) compared to vehicle (brown) (all 100 µM, *n* = 9). The results are from three independent experiments measured in triplicates. **h** Significant knockdown of *ATP5A* gene expression in *ATP5A* siRNA treated PC-3 cells (blue) compared to *Ctrl* siRNA PC-3 cells (brown) (*n* = 3 per condition, *p* < 0.0001). **i** Significantly reduced [^11^C]J147 uptake in *ATP5A* siRNA treated PC-3 cells (blue) compared to *Ctrl* siRNA treated cells (brown) (*n* = 6 independent cell samples per condition, *p* < 0.0001). Data are represented as mean ± s.d. Two-tailed Student’s *t* tests with Welch correction and correction for multiple comparisons using Holm-Šídák method (**d**, **f**, **h**, **i**). one-way ANOVA with Dunnett’s multiple comparisons test (**e**, **g**). **g**, **h** Measured in 3 independent biological experiments.

J147 and J147 precursor were synthesised as reported previously^39^. The radiosynthesis of [^11^C]J147 was performed using [^11^C]MeOTf and NaOH (5 M, aq. sol.) in acetonitrile at room temperature. (Fig. 1a) High radiochemical purity (>95%) was achieved after semipreparative high-performance liquid chromatography purification. The molar activity of [^11^C]J147 was 257 ± 154 GBq/µmol (*n* = 50) at the end of synthesis. [^11^C]J147 demonstrated good stability in its formulation (5% EtOH, 25% PEG300, 70% PBS, pH 7.4) two hours after tracer production. (Supplementary Figs. S1, 2)

Firstly, [^11^C]J147 was evaluated by in vitro autoradiography using interscapular BAT and visceral WAT dissected from healthy mice. Higher radioactive signals were observed in BAT than WAT (Fig. 1b). The in vitro specificity of [^11^C]J147 was assessed by including blocking conditions, where the tissues were either incubated with only [^11^C]J147, or both [^11^C]J147 and an excess of unlabelled J147. Significant blocking was found for both BAT (*P* = 0.0002, *n* = 6) and WAT (*P* < 0.0001, *n* = 6). (Fig. 1b, d) To assess whether we can use [^11^C]J147 to monitor BAT and WAT activation, autoradiography studies were conducted using [^11^C]J147 in interscapular BAT and visceral WAT dissected from mice which were treated with the β_3_-adrenergic receptor agonist CL316,243 (1 mg/kg, i.p., 24 h and 1 h before tissue collection). (Fig. 1c) The activation of adipose tissues resulted in a significantly increased accumulation of [^11^C]J147 in BAT (*P* = 0.0145, *n* = 6) and in WAT (*P* = 0.0207, *n* = 6). (Fig. 1d) Similar to non-activated conditions, [^11^C]J147 accumulation was significantly higher in interscapular BAT than visceral WAT (*P* = 0.0015). (Fig. 1f) Moreover, to corroborate the specificity of [^11^C]J147 accumulation in activated BAT, we co-incubated slices of intrascapular BAT of CL316,243-treated mice with [^11^C]J147 (3–5 nM) and our recently developed partial inhibitor of ATP synthase, CJ1-34^40^, at 10 µM, 100 µM and 1 mM. BCPP-EF, which binds to complex I, was used as a negative control. CJ1-34 competed with [^11^C]J147 binding in a concentration-dependent manner, while no significant blocking was found for BCPP-EF (*P* = 0.973) (Fig. 1e). Representative radioautographs are shown in Supplementary Fig. S3.

The specificity was further investigated in an in vitro functional assay with isolated mitochondria, monitoring ATP hydrolysis^41^. Both J147 and oligomycin A, a full inhibitor of ATP synthase, significantly reduced the mitochondrial ATP synthase activity at 100 µM. The mitochondrial complex I inhibitor rotenone had no effect at the same concentration (*P* = 0.951) (Fig. 1g).

The in vitro specificity of [^11^C]J147 was further evaluated via *ATP5A* knockdown experiments in PC-3 cells. PC-3 cells were transfected with *control* (*Ctrl*) or *ATP5A* siRNA for 24 h, resulting in a significant reduction of *ATP5A* mRNA expression in *ATP5A* siRNA transfected PC-3 cells compared to *Ctrl* siRNA transfected cells (*P* < 0.0001, *n* = 3). (Fig. 1h). Cell uptake of [^11^C]J147 was significantly lower in the *ATP5A* siRNA-transfected than the *Ctrl* cells (*P* < 0.0001), again confirming the specificity of [^11^C]J147. (Fig. 1i)

### Interscapular BAT and visceral WAT display increased ATP5A and UCP1 expression after activation with CL316,243

**Fig. 2:**
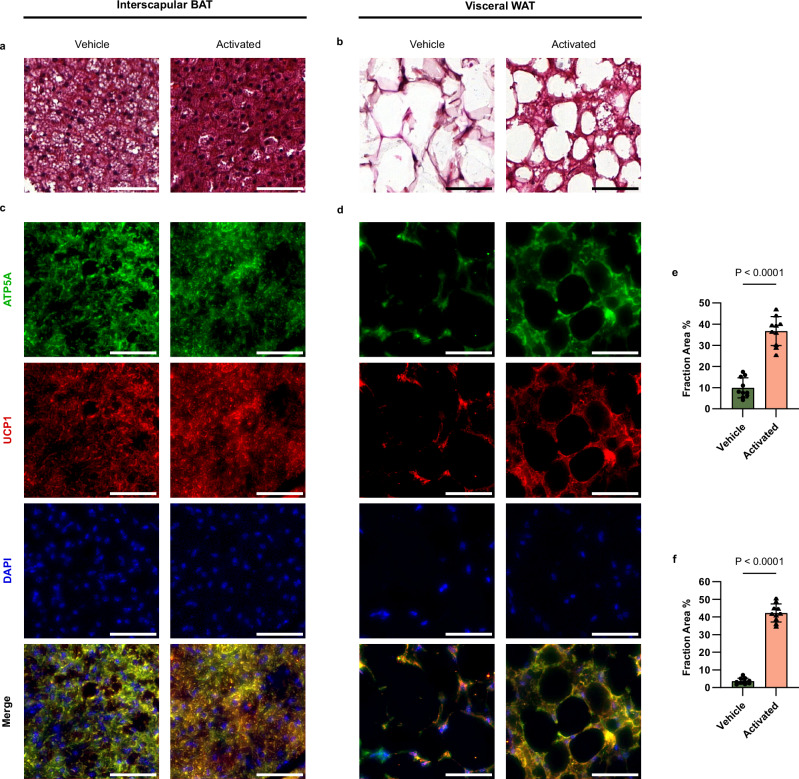
ATP5A expression in interscapular BAT and visceral WAT. H&E staining (scale bar 100 µm) of interscapular BAT (**a**), visceral WAT (**b**), either vehicle-treated or activated with CL316,243 (1 mg/kg, i.p., 24 h and 1 h) as indicated. Immunofluorescence staining of ATP5A (green), UCP1 (red), DAPI (blue) and merged ATP5A and UCP1 (yellow) in vehicle-treated or CL316,243-treated interscapular BAT (**c**) and visceral WAT (**d**) of Balb/c mice. Scale bar, 100 µm. Semiquantification of the immunofluorescence images of ATP5A in interscapular BAT (**e**, *n* = 10 images per condition, *p* < 0.0001) and visceral WAT (**f**, *n* = 10 images per condition, *p* < 0.0001) depicted in fraction area percentage. *n* = 2 mice per group, 5 images per animal. Data are represented as mean ± s.d. Two-tailed Student’s *t* test with Welch correction (**e**, **f**).

Further characterisation of the same interscapular BAT and visceral WAT showed different cellular morphology between BAT and WAT as indicated by cell size and number in the H&E staining. (Fig. 2a, b) Particularly, adipocytes in WAT were larger compared to BAT and both exhibited increased number of cells after β_3_-adenergic agonist activation. The immunohistochemistry results for ATP5A corroborate the results we observed in the autoradiography studies. (Figs. 1b–f and 2) Activation with CL316.243 resulted in significantly increased ATP5A expression in interscapular BAT (*P* < 0.0001, *n* = 10; Fig. 2e), visceral WAT (*P* < 0.0001, *n* = 10; Fig. 2f) and inguinal WAT (*P* < 0.0001, *n* = 10; Supplementary Fig. S4) compared to the non-activated tissues. The expression of UCP1 in the same tissues followed a similar trend with higher UCP1 expression in all the tested activated BAT (*P* < 0.0001, *n* = 10) and WAT (*P* < 0.0001, *n* = 10) compared to the non-activated tissues. (Supplementary Fig. S5)

### [^11^C]J147 detects interscapular BAT in healthy mice in vivo

**Fig. 3:**
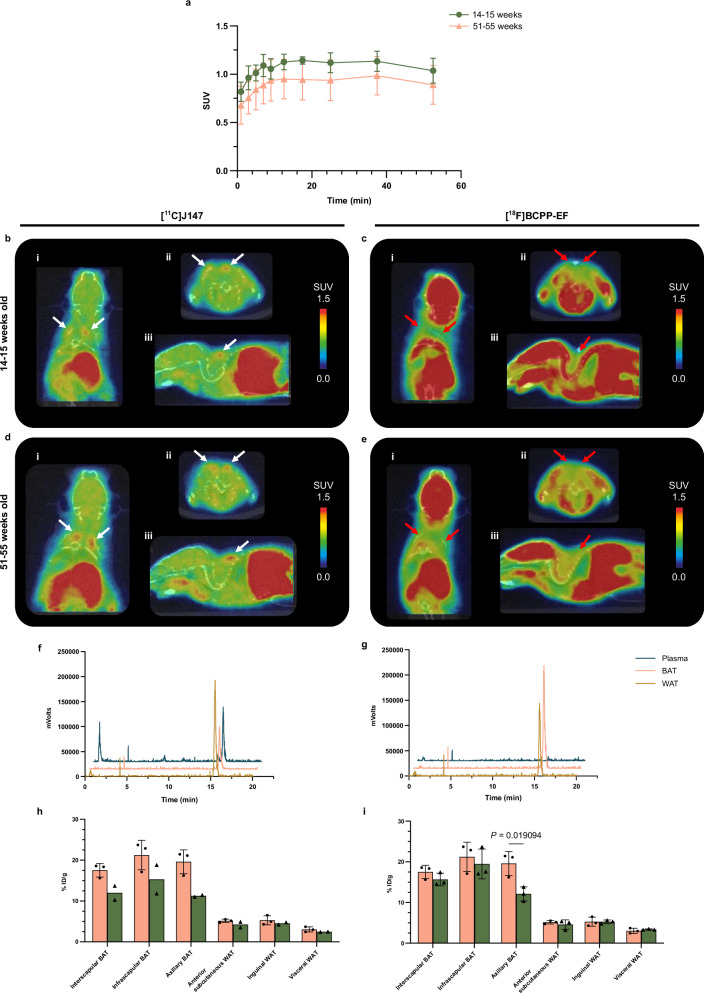
Comparison of [^11^C]J147 and [^1^^8^F]BCPP-EF imaging of BAT in young and old mice. **a** Time activity curve (TAC) of [^11^C]J147 in interscapular BAT in 14–15 weeks old (*n* = 3) and 51–55 weeks old (*n* = 3) C57BL/6;C3H mice. **b**–**e** Representative averaged images (time frame 0–60 min) of interscapular BAT (white arrows or red arrows if not visible) imaged with [^11^C]J147 or [^18^F]BCPP-EF depicted in coronal (i), transverse (ii), and sagittal plane (iii) of C57BL/6;C3H mice. **f**, **g** Chromatogram of the column switch radioHPLC displaying the radiometabolite results. Artefact produced around 4 min as result of column switching. Analysis of ^11^C-labelled species in plasma, WAT and BAT as indicated at 5 min p.i. of [^11^C]J147 (**f**; metabolite 1: retention time (RT), 0.74 min; [^11^C]J147: RT, 15.5 min) and at 30 min p.i. of [^11^C]J147 (**g**). **h**, **i** Biodistribution of [^11^C]J147 in CL316,243-treated Balb/c mice (*n* = 3) reported as %ID/g (pink bars). For blocking conditions, animals were injected with J147 (**h**; i.v., 4 mg/kg, *n* = 2 mice, green bars) or CJ1-34 (**i**; i.v., 4 mg/kg, *n* = 3 mice, green bars) 3 min before [^11^C]J147 injection. Data for *n* = 3 are represented as mean ± s.d. Comparison between baseline and blocking by unpaired t-tests (two-tailed) with Welch correction and correction for multiple comparisons using Holm-Šídák method.

Encouraged by the in vitro findings, dynamic PET imaging studies were conducted by administration of [^11^C]J147 via tail vein injection into 14–15 and 51–55 weeks old C57BL/6;C3H mice and compared with mitochondrial complex I tracer [^18^F]BCPP-EF. The interscapular BAT was clearly visualised by [^11^C]J147 with prolonged tracer retention as depicted by the averaged PET images and the time activity curve (TAC). (Fig. 3a, b, d) The maximal accumulation of [^11^C]J147 was reached within 7 min post-injection (p.i.) with standardised uptake value (SUV) of 1.19 ± 0.07 (average ± SD, *n* = 3) in the BAT of the group of younger mice and an SUV of 0.99 ± 0.16 (average ± SD, *n* = 3) in BAT in the older mice. Clearance of radioactivity from the BAT was low compared to the brain during the PET scans. (Supplementary Fig. S6) On the other hand, [^18^F]BCPP-EF could not visualise interscapular BAT in mice aged 14–15 or 51–55 weeks, but it exhibited high uptake in the brain. (Fig. 3c, e) In short, we demonstrated that [^11^C]J147 clearly visualised interscapular BAT in healthy young and old mice, outperforming the mitochondrial complex I PET tracer [^18^F]BCPP-EF.

A radiometabolite study was conducted to determine whether the PET signal observed is due to the intact [^11^C]J147 or potentially radiometabolites. Tissue analysis at 5 min and 30 min p.i. by column switch radioHPLC displayed only intact [^11^C]J147 (RT = 15.5 min, Supplementary Fig. S7) in interscapular BAT and visceral WAT. (Fig. 3f, g) A polar radiometabolite (RT = 0.74 min) was detectable in the plasma as early as the 5 min p.i. (Fig. 3f) At 30 min p.i., only metabolised [^11^C]J147 was detectable in plasma (Fig. 3g), which aligns with the metabolism of J147 in mouse liver microsomes^42^. Tissue and plasma samples were in addition analysed via radioTLC (Supplementary Fig. S8), confirming that the radioactivity in BAT and WAT resulted mainly from intact [^11^C]J147.

To further investigate the specificity of [^11^C]J147 binding in vivo, competition binding studies were conducted with CJ1-34 (4 mg/kg) and J147 (4 mg/kg) using CL316,243-treated mice (13–15 weeks old). Chemical structures of CJ1-34 and J147 can be found in Supplementary Fig. S9. Mice were euthanised 70 min p.i. and the dissected adipose tissues were analysed in a gamma counter (Fig. 3h, i). Blocking with CJ1-34 (*n* = 3) resulted in a significant reduction of [^11^C]J147 accumulation in axillary BAT by 38% (*P* = 0.019). The reduction in interscapular and infrascapular BAT was not significant. Blocking with J147 (*n* = 2) indicated blocking to a similar extent. Notably, the PET time–activity curve under CJ1-34 blocking conditions (Supplementary Fig. S10) demonstrated a marked reduction in tracer uptake at early time points, followed by a gradual increase over the course of the PET scan. At later time points, tracer uptake approached the levels observed in the absence of the blocker. This pattern is consistent with competitive tracer binding during the early phase and reflects the lower binding potency of CJ1-34 relative to J147, resulting in reduced and less sustained target occupancy.

### [^11^C]J147 displays higher tracer uptake in interscapular BAT compared to [^18^F]FDG

**Fig. 4:**
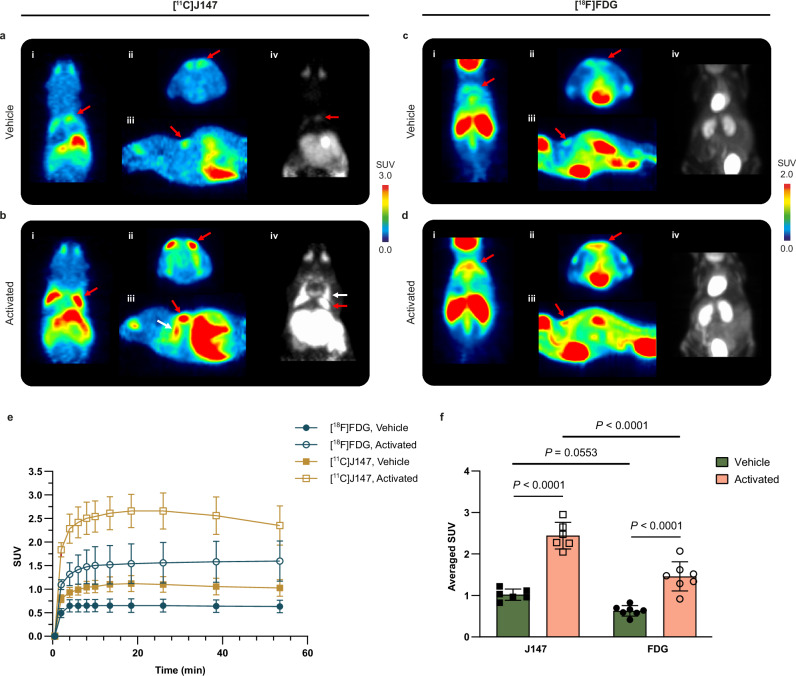
Comparison of [^11^C]J147 and [^18^F]FDG imaging of BAT. In vivo PET imaging study with [^11^C]J147 and [^18^F]FDG in vehicle-treated or CL316,243-treated mice. Representative images depicting coronal (i), transverse (ii), sagittal (iii) plane, and maximum intensity projection (iv). Interscapular BAT is indicated with red arrows and combined infrascapular and axillary BAT with white arrows. Representative averaged PET images (time frame: 0–60 min) of vehicle-treated mice (**a** [^11^C]J147; **c** [^18^F]FDG) and CL316,243-treated mice (**b** [^11^C]J147; **d** [^18^F]FDG). **e** Time activity curves of [^11^C]J147 (*n* = 6 mice per group) and [^18^F]FDG (*n* = 7 mice per group) in interscapular BAT in vehicle-treated and CL316,243-treated groups. **f** Averaged SUV of [^11^C]J147 (*n* = 6 per group) and [^18^F]FDG (*n* = 7 per group) in interscapular BAT of the vehicle-treated and CL316,243-treated groups. CL316,243 treatment significantly increased interscapular BAT SUV for [^11^C]J147 (*n* = 6, *p* < 0.0001) and [^18^F]FDG (*n* = 7, *p* < 0.0001). Interscapular BAT averaged SUV was significantly higher for [^11^C]J147 than for [^18^F]FDG in the CL316,243-treated groups (*p* < 0.0001). Data are represented as mean ± s.d. one-way ANOVA with Tukey’s post-hoc correction for multiple comparison.

In vivo PET imaging studies were conducted with vehicle-treated (vehicle group) or CL316,243-treated (activated group) Balb/c mice using [^11^C]J147 or the gold standard [^18^F]FDG. Both tracers demonstrated increased tracer uptake in interscapular BAT in the activated group compared to the vehicle group. (Fig. 4a–d) However, in the maximum intensity projection, interscapular BAT was only visible with [^11^C]J147 but not with [^18^F]FDG. (Fig. 4a–d; iv) Moreover, TACs indicated higher radioactivity in BAT with [^11^C]J147 than [^18^F]FDG in both vehicle- and CL316,243-treated mice in the entire examined period of 60 min. (Fig. 4e) Comparing the averaged SUV of [^18^F]FDG and [^11^C]J147 in vehicle and activated animals, [^11^C]J147 demonstrates a 1.6-fold increase in vehicle-treated animals (*P* = 0.055) and a significantly increased uptake in activated animals compared to [^18^F]FDG (*P* < 0.0001). (Fig. 4f) The in vivo dynamic [^11^C]J147 PET imaging for visualisation of interscapular BAT (mice treated with vehicle or CL316,243) at different time frames are presented in Supplementary Fig. S11 of the supplementary material. [^11^C]J147 and [^18^F]FDG TACs of interscapular BAT of male and female mice in separate groups are shown in Supplementary Fig. S12. No significant differences were found for [^11^C]J147 between male and female mice for imaging interscapular BAT. (two-way ANOVA with Šídák’s post-hoc correction for multiple comparison, *P* = 0.1682; Supplementary Fig. S13) No differences in appearance, weight and surface area of interscapular BAT were detected between vehicle- and CL316,243-treated mice (Supplementary Figs. S14 and S15).

### Additional BAT and WAT depots can be visualised with [^11^C]J147 but not with [^18^F]FDG

**Fig. 5:**
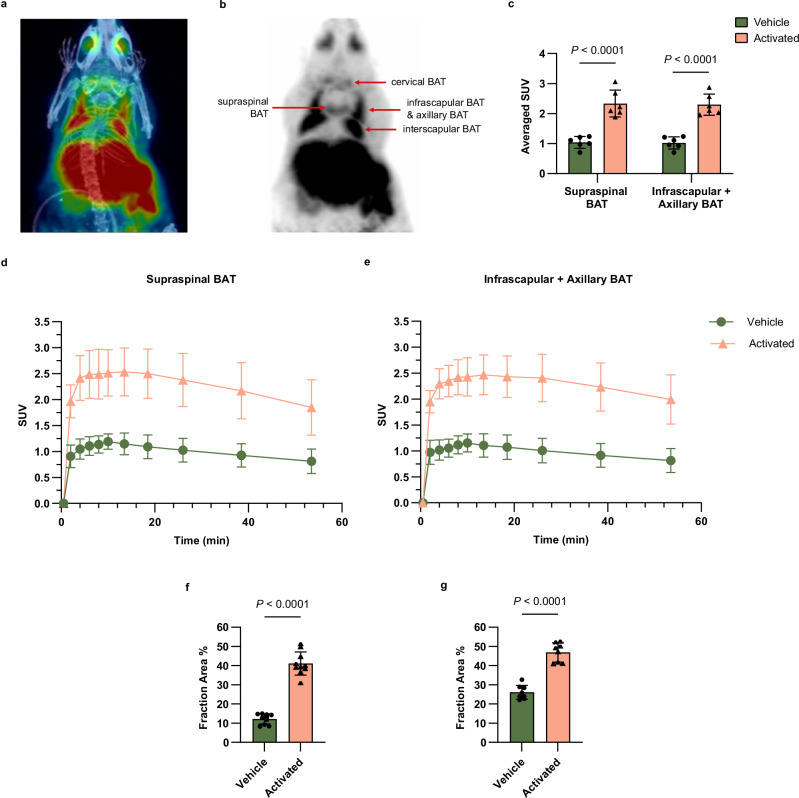
PET imaging of different brown adipose depots with [^11^C]J147. **a** Averaged PET image (time frame 0–60 min) of [^11^C]J147 in CL316,243-treated Balb/c mice, superimposed on CT (grey). **b** Maximum intensity projection (MIP) with the following BAT depots labelled: supraspinal BAT, cervical BAT, combined infrascapular and axillary BAT, and interscapular BAT. **c** Averaged SUV of the supraspinal BAT and combined infrascapular and axillary BAT in vehicle-treated or CL316,243-treated Balb/c mice (*n* = 6 per group). CL31,243 treatment significantly increased the averaged SUV in both supraspinal BAT and combined infrascapular and axillary BAT compared with vehicle controls (*p* < 0.0001 for both tissue regions). Data are represented as mean ± s.d., one-way ANOVA with Tukey’s post-hoc correction for multiple comparison. **d** Time activity curve of the supraspinal BAT (*n* = 6 per group). **e** Time activity curve of the combined infrascapular and axillary BAT (*n* = 6 per group). Semiquantification of the immunofluorescence images of ATP5A in infrascapular BAT (**f**, vehicle *n* = 10 and activated *n* = 10) and axillary BAT (**g**, vehicle *n* = 8 and activated *n* = 8) depicted in fraction area percentage. Activation significantly increased ATP5A-positive fraction area in both infrascapular (*p* < 0.0001) and axillary (*p* < 0.0001) BAT compared with vehicle controls. Data are represented as mean ± s.d. Two-tailed Student’s *t* test with Welch correction (**f**, **g**). Same mice as in Fig. 4.

In addition to higher tracer uptake of [^11^C]J147 in interscapular BAT compared to [^18^F]FDG, another major difference in BAT imaging was the observation of other BAT depots with [^11^C]J147 in CL316,243-treated mice. Visible radiotracer accumulation was detected in the supraspinal, cervical, axillary, and infrascapular BAT depots (Fig. 5a, b), with particularly elevated uptake observed in the supraspinal, axillary and infrascapular regions (Fig. 5c–e). The radioactive signal in axillary and infrascapular BAT were not separable in the PET images due to the limited spatial resolution and thus appear as one region of high intensity in the maximum intensity projection (Fig. 5a, b) and therefore were analysed together in the TAC. (Fig. 5c). In non-activated mice, we found that not only interscapular BAT but also inguinal WAT could be identified with [^11^C]J147, which was not the case with [^18^F]FDG. (Supplementary Figs. S16–18).

BAT activation with CL316,243 resulted in significantly increased [^11^C]J147 uptake in the supraspinal and axillary & infrascapular BATs (*P* < 0.0001, *n* = 6) (Fig. 5c). Moreover, the immunohistochemistry of ATP5A in the infrascapular and axillary BATs (*P* < 0.0001, *n* = 10 and 8, respectively) also demonstrated significantly increased ATP5A expression in the activated adipose tissues. (Fig. 5f, g and Supplementary Fig. S19)

### Differential biodistribution profile in [^18^F]FDG and [^11^C]J147

**Fig. 6:**
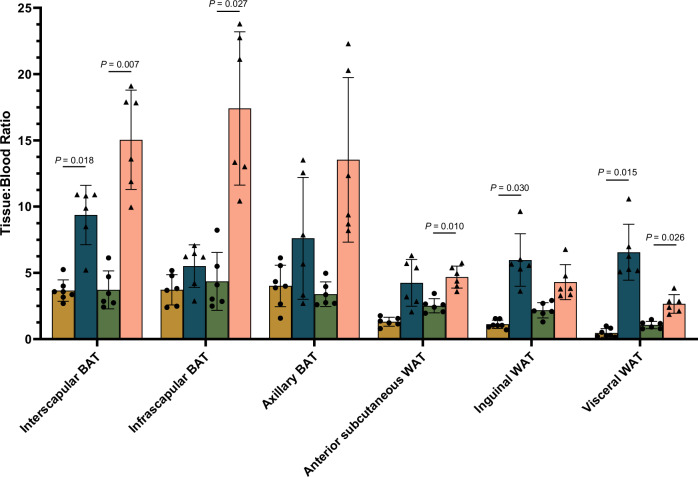
Biodistribution of [^11^C]J147 and [^18^F]FDG in CL316,243-treated Balb/c mice. Biodistribution of [^18^F]FDG in vehicle-treated (*n* = 7 mice) or CL316,243-treated Balb/c mice (*n* = 6 mice) and biodistribution of [^11^C]J147 in vehicle-treated (*n* = 6 mice) or CL316,243-treated Balb/c mice (*n* = 6 mice) reported as tissue-to-blood ratio (mean ± SD). Multiple unpaired t-tests (two-tailed) with Welch correction and correction for multiple comparisons using Holm-Šídák method.

To study the biodistribution of the tracers in more detail, the vehicle and CL316,243-treated mice from the PET experiments shown in Figs. 4 and 5 (*n* = 6 or 7 per group) were euthanised under anaesthesia after the PET scans at 70 min p.i. and tissues were dissected. For all the collected BAT depots, [^11^C]J147 demonstrated higher tissue/blood ratio than [^18^F]FDG (Fig. 6), consistent with the PET imaging results. In comparison with the vehicle-treated mice, the CL316,243-treated mice exhibited a significant increase in tracer tissue/blood ratio in interscapular BAT (*P* = 0.007) and infrascapular BAT (*P* = 0.027), whilst axillary BAT showed an apparent but non-significant increased tissue/blood ratio after activation (3.99-fold increase, *P* = 0.111). [^18^F]FDG demonstrated a modest increase in BAT depots following activation, with a notable enhancement observed in interscapular BAT (2.56-fold increase, *P* = 0.018). However, [^18^F]FDG did demonstrate a significant increase in both inguinal and visceral WAT (*P* = 0.030 and *P* = 0.015, respectively) after activation. Conversely, the increase of [^11^C]J147 in activated inguinal WAT was not significant (*P* = 0.111), although a trend of increased tracer tissue/blood ratio compared to nonactivated WAT was observed.

The potential influence from the anaesthetics used on tracer uptake was investigated by performing the similar biodistribution study in awake male animals. [^11^C]J147 or [¹⁸F]FDG was administrated in conscious mice (*n* = 3 for each group) which were euthanised under anaesthesia at 15 min p.i. to collect the adipose tissues. The results showed consistently higher uptake of [^11^C]J147 in all fat depots compared with [¹⁸F]FDG, aligning with the biodistribution patterns observed under anaesthesia (Supplementary Fig. S20).

### Evaluating the potential application of [^11^C]J147 in the STAM model and GLP-1 treatment

**Fig. 7:**
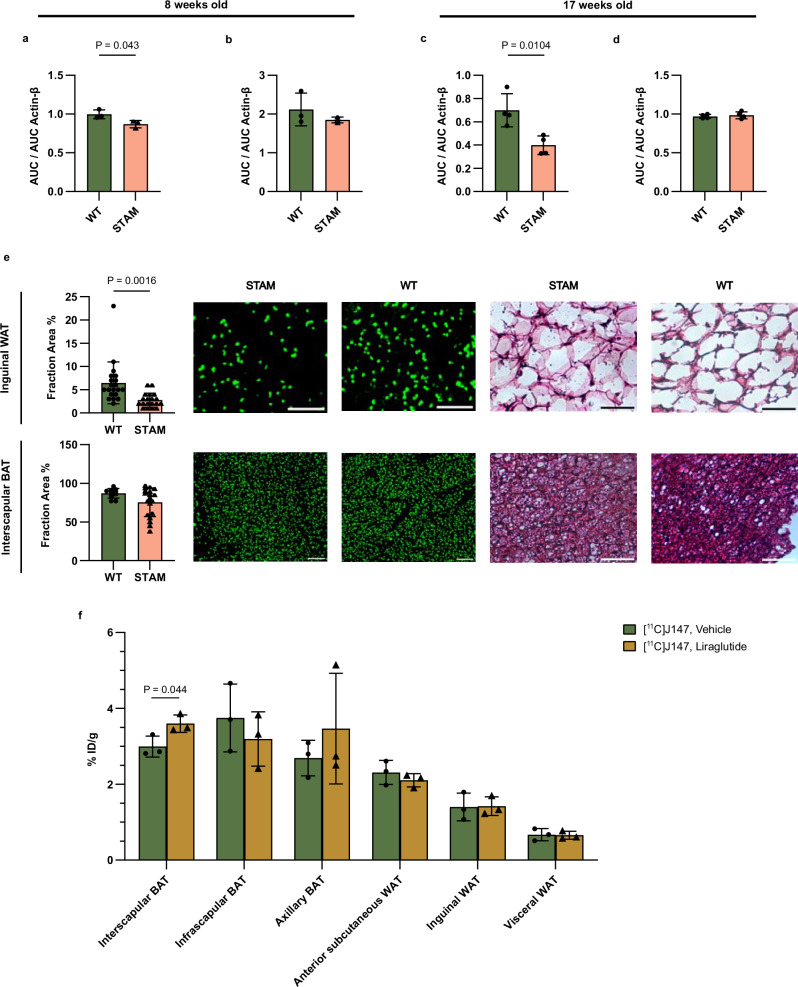
ATP5A expression in the STAM model and after GLP-1 treatment. Semiquantification of ATP5A expression via western blot in inguinal WAT (**a**, **c**) and interscapular BAT (**b**, **d**) in 8 weeks old (**a**, **b**; *n* = 3 mice per group) or 17 weeks old (**c**, **d**; *n* = 4 mice per group) STAM (pink) and control (green) mice. **e** Semiquantification of ATP5A via immunohistochemistry in inguinal WAT and interscapular BAT and H&E staining (scale bar 100 μm) in 17 weeks old STAM (*n* = 19 images from inguinal WAT and *n* = 20 images from interscapular BAT, pink) or control (*n* = 20 images from inguinal WAT and *n* = 10 images from interscapular BAT, green) mice. *n* = 2 mice per group. **f** Biodistribution of [^11^C]J147 in adipose tissues in vehicle-treated (*n* = 3, green) and liraglutide-treated Balb/c mice (*n* = 3, brown) reported as % injected dose/weight. Data are represented as mean ± s.d., two-tailed Student’s *t* test with Welch correction.

To further investigate the relevance of ATP synthase as a potential imaging target in metabolic disease, we studied the ATP5A expression in various adipose tissues of a mouse model of diabetes, namely the mouse model of non-alcohol steatohepatitis and hepatocellular carcinoma (STAM mice)^43^. ATP5A expression in inguinal WAT was significantly reduced compared with controls at 8 weeks (*P* = 0.043) and showed a further decrease at 17 weeks of age (*P* = 0.0104) by Western blotting studies (Fig. 7a, c; Supplementary Fig. S21). Similar results were found by immunohistochemistry at 17 weeks of age for the inguinal WAT (Fig. 7e, *P* = 0.0016). In contrast, ATP5A expression in interscapular BAT did not differ from age-matched control mice at either 8 or 17 weeks of age, as assessed by both Western blotting and immunohistochemistry (Fig. 7b, d, e).

We furthermore investigated whether ATP synthase has potential as an imaging target for therapy monitoring in metabolic disease. Therapeutic compounds that activate the glucagon-like peptide-1 (GLP-1) receptor, including liraglutide, are increasingly recognised for their benefits in diabetes and obesity management^44^. To assess how liraglutide influences ATP synthase expression under physiological conditions, we conducted biodistribution studies in liraglutide-treated (0.2 mg/kg, s.c., at 24 h and 4 h), 12-week-old female Balb/c mice. At 70 min p.i., [^11^C]J147 uptake was largely comparable to the control across most adipose tissues. However, interscapular BAT exhibited a significant increase in tracer accumulation following this short liraglutide exposure (*P* = 0.044; Fig. 7f). Moreover, significantly increased brain uptake of [^11^C]J147 (*P* = 0.0028; Supplementary Fig. S22) was observed in liraglutide treated mice compared to the controls.

## Discussion

Recent studies indicate that, in addition to classical thermogenesis mediated by UCP1-driven uncoupled respiration, certain thermogenic adipocytes can generate heat through ATP-dependent futile cycles^30,45,46^. This mechanism has been reported in both brown and white adipose tissues where beige adipocytes are recruited following cold exposure or β_3_-adrenergic activation. For example, ATP synthase levels were reported to increase in white adipose depots following a 7-day CL316,243 treatment^47^. Moreover, two independent studies identified populations of adipocytes in inguinal fat rely on futile cycle-dependent thermogenesis under cold exposure^31,48^. Notably, currently available PET imaging targets, including glucose metabolism, translocate protein and mitochondrial membrane potential among others, are not directly monitoring ATP synthase-related processes and thus are not well-suited for detecting adipocytes engaged in ATP-dependent futile cycles. This study investigated ATP synthase as a target for imaging adipose tissue using [^11^C]J147 as a PET tracer.

The potential of mitochondrial ATP synthase as a target for adipose tissue imaging was supported by our immunohistochemistry studies, where we observed significantly increased ATP5A expression after β_3_-adrenergic activation of the adipose tissues. This aligned well with the observed tracer accumulation of [^11^C]J147 in non-activated and activated interscapular BAT in the autoradiography studies. In vivo PET imaging revealed that [^11^C]J147 has higher uptake in younger mice than older mice, which correlates with the changes in BAT content in mice at different ages^49,50^. [^11^C]J147 accumulation in BAT significantly increased after β_3_-adrenergic activation, independent of whether the mice were under anaesthesia or awake. Furthermore, no significant effect was found on [^11^C]J147 uptake for gender (male vs female). These results lay the groundwork for the application of ATP synthase PET tracer [^11^C]J147 in metabolic diseases. Additionally, the in vivo competition binding studies with non-radioactive J147 and compound CJ1-34 supports the target-mediated binding of [^11^C]J147 towards adipose tissues. The relatively modest effects observed may be attributed to incomplete receptor occupancy, partial non-specific uptake of [¹¹C]J147, and the high abundance of ATP synthase, which may require higher doses and continuous infusion, rather than a single bolus injection, to achieve adequate target engagement.

The complex I PET tracer, [^18^F]BCPP-EF, was included in this study to compare it with [^11^C]J147. In contrast to the high uptake of [^11^C]J147 in interscapular BAT, the uptake of [^18^F]BCPP-EF was not visible in similarly aged mice. The low uptake of [^18^F]BCPP-EF may be attributable to the reduced content of BAT in older aged mice (14–15 and 51–55 weeks old) in comparison to the previously published studies conducted in 6–8 weeks cold mice^16^. Moreover, when comparing to the current golden standard [^18^F]FDG, we observed that [^11^C]J147 achieved higher tracer uptake in all BAT depots than [^18^F]FDG in both non-activated and activated mice. Infrascapular and axillary BAT had a significant increase in [^11^C]J147 uptake after activation, which was not observed for [^18^F]FDG. Particularly, the present study provides evidence that [¹¹C]J147 PET can visualise the non-activated inguinal WAT, a depot that has been shown to generate beige adipocytes. Previous studies have revealed that reduced mitochondrial function and content were observed in WATs of diabetic mice^51–53^, rats^54^ and humans^55–57^. In agreement with the previous observations, we found decreased ATP synthase expression in inguinal WAT at both the early and late stages of a mouse model of metabolic disease (STAM). Therefore, our findings support that mitochondrial impairment could be involved in metabolic disease and [¹¹C]J147 has the potential to pave the way to image beige and adipocyte differentiation, a critical process related to diabetic disease^58,59^.

The relatively low [^11^C]J147 tracer uptake in WAT adipose tissues in our biodistribution studies could be attributed to the relatively short duration of activation in our study. Previous studies demonstrated an increased abundance of ATP synthase subunits, including ATP5A, in beige fat mitochondria following prolonged activation (one week of cold exposure or 10 days of CL316,243 treatment)^47,60^. Moreover, the oxidative phosphorylation profile of beige fat mitochondria differs from classical brown fat, with ATP synthase being the dominant protein. Recently, the use of GLP-1 receptor agonists, such as liraglutide, have gained significant interest in diabetes and obesity^61^. Liraglutide demonstrated enhanced mitochondrial respiration and stimulation of mitochondrial biogenesis, suggesting that GLP-1 signalling can improve mitochondrial function in adipocytes^62,63^. The present study investigated the effect of liraglutide treatment on ATP synthase expression in healthy mice. Our results demonstrated a slight but significant increase of [^11^C]J147 uptake in interscapular BAT in biodistribution studies in mice with a short-term treatment regimen (0.2 mg/kg liraglutide, s.c., at 24 h and 4 h before [^11^C]J147 injection). This suggests that [^11^C]J147 could potentially be a useful PET tracer to monitor ATP synthase expression during mitochondrial biogenesis. Future studies are warranted to determine whether long-term GLP-1 treatment alters ATP synthase expression, given that the long-term effects of GLP-1 therapies on humans are not fully understood.

In conclusion, ATP synthase is a promising target for imaging adipose tissues. [^11^C]J147 represents a significant advancement as a potential futile cycle tracer that can image activated and non-activated adipose tissues and can detect ATP synthase density in several BAT depots (interscapular, supraspinal, axillary, and infrascapular BAT) in mice. The specificity of [^11^C]J147 towards ATP synthase was confirmed by in vitro autoradiography and cell knockdown studies and in vivo competition binding studies. Radiosignals in BAT and WAT resulted from intact [^11^C]J147 as shown by the radiometabolite studies. Moreover, both in vitro and in vivo studies displayed significantly increased [^11^C]J147 uptake in activated adipose tissues compared to non-activated adipose tissues. Particularly, [^11^C]J147 PET could visualise interscapular BAT and inguinal WAT without prior activation and has significantly higher uptake compared to [^18^F]FDG in interscapular BAT and other BAT depots. [^11^C]J147 outperformed [^18^F]BCPP-EF for detecting interscapular BAT in mice. These findings show promise for [^11^C]J147 as a tool for imaging adipose tissue and facilitating drug development for metabolic disease.

## Methods

### Ethical statement

The animal experiments were carried out in compliance with the ARRIVE guidelines and Swiss Animal Welfare legislation, according to Art.18 Tierschutzgesetz (TSchG), Art. 141 Tierschutzverordnung (TschV), Art. 30 Tierversuchsverordnung (TVV) (all Switzerland), and were approved by the cantonal Veterinary Office of Zurich, Switzerland, under the license 34491 and 36342 (National Number).

### Animals and tissue preparation

Female C57BL/6;C3H mice (*n* = 7, 14–15 weeks old, 19–27 g and *n* = 7, 51.0–55.4 weeks old, 22–35 g) were supplied by Jackson laboratory and female Balb/c (*n* = 18, 11–13 weeks old, 19–22 g) and male Balb/c (*n* = 14, 13 weeks old, 22–26 g) were supplied by Charles River (Sulzberg, Germany). All animals were housed under specific pathogen-free conditions at 23 °C, 48% humidity, with a 12 h light/dark cycle and ad libitum food (chow diet, 3437.PX.L15, Granovit AG, Switzerland) and water access. The animals were allowed to acclimatise for 1 week before the start of the experiments.

In vitro autoradiography. BAT and WAT tissues – either vehicle-treated or activated with β_3_-adrenergic receptor agonist CL316,243 (formulated in PBS, 1 mg/kg, i.p., 24 h and 1 h prior tissue collection) – were collected from 5-weeks-old C57BL/6; C3H mice. Tissues embedded in OCT medium were prepared as 10 µm thick sections on a cryostat (Cryo-Star HM 560 MV; Microm, Thermo Scientific, Wilmington, DE, USA). Tissue slices were adsorbed on SuperFrost Plus glass slides (Menzel, Braunschweig, Germany) and stored at −80 °C.

Haematoxylin and eosin staining and immunohistochemistry. BAT and WAT tissues – either vehicle-treated or activated with β_3_-adrenergic receptor agonist CL316,243 (formulated in PBS, 1 mg/kg, i.p., 24 h and 1 h prior tissue collection) – were collected from 11 to 13 weeks old Balb/c mice. Tissues were dissected and placed overnight at 4 °C in formaldehyde solution 4% (buffered, pH 6.9, Merck; Darmstadt, Germany). Following fixation, tissues were transferred to a 10% (w/v) sucrose (Thermo Scientific, Wilmington, DE, USA) solution in PBS (Gibco) for 24 h at 4 °C then transferred to a 30% (w/v) sucrose solution in PBS for an additional 24 h at 4 °C. Following cryoprotection, tissues were embedded in OCT medium and cut as 10 µm thick sections on a cryostat (Cryo-Star HM 560 MV; Microm, Thermo Scientific, Wilmington, DE, USA). Tissue slices were adsorbed on SuperFrost Plus glass slides (Menzel, Braunschweig, Germany) and stored at −80 °C. The detailed experimental process for haematoxylin and eosin staining and immunohistochemistry are presented in the Supplemental part.

STAM animals. NASH-HCC was induced in male C57BL6/NRj (Janvier Labs) mice by a single subcutaneous injection of 200 µg streptozotocin (S0130, Sigma) at 2 days after birth and feeding with high fat diet (SAFE U8978 Version 19, 58.6& Energy from lipids) ad libitum after 4 weeks of age. Male litter without any treatment were housed as control animals. 6 animals were used at 8 weeks of age, and 8 animals were used at 17–18 weeks of age. The number of mice per group per time point for biochemical and histological analyses was three to four.

Drugs. Activation of BAT was performed via i.p. injection of β_3_-adrenergic agonist CL316,243 (AdipoGen; San Diego, USA) in PBS (1 mg/kg dose) 24 h and 1 h prior to PET scan. The non-activated (vehicle-treated) mice were injected identical with PBS. The study protocol is depicted in Supplementary Fig. S23.

Liraglutide (Sigma-Aldrich, SML 3925) was dissolved in NaCl 0.9% solution (B. Braun Medical AG, Sempach) at a concentration of 0.200 mg/kg and injected s.c. at 24 h and 4 h before PET scan or tissues sampling.

CJ1-34 and J147 were dissolved in 5% DMSO (VWR Chemicals; Radnor, USA), 5% TWEEN 80 (P4780. Sigma Life Science; St. Louis, USA) and 25% PEG300 (Scharlau Chemie S.A.; Spain) in water for injection (GE HealthCare; Chicago, USA) and administered i.v. at a concentration of 4 mg/kg 3 min before radiotracer injection. A formulation mix (8% TWEEN 80, 40% PEG300 in water for injection) was prepared to facilitate solution preparation.

### Chemical synthesis

Compound J147 was synthesised following the procedure reported by Chen et al.^33^. CJ1-34 was synthesised following the procedure as reported in Jie et al.^40^. The synthesis of BCPP-EF was performed as reported by Harada et al.^64^.

### Radiosynthesis

[^11^C]J147. The radiosynthesis of [^11^C]J147 was accomplished in a one-step procedure via *O-*methylation of the J147 hydroxyl precursor using [^11^C]CH_3_OTf. The detailed radiolabeling process and quality control are presented in the Supplemental part.

[^18^F]BCPP-EF was radiosynthesis by aliphatic nucleophilic substitution using its corresponding tosylate precursor in analogy to a previously published procedure by Harada et al.^64^. The detailed process is included in the Supplemental part and Supplementary Figs. 24–26.

[^18^F]FDG was prepared by University Hospital Zurich from routine production for clinical use.

### In vitro autoradiography

Tissue slides were thawed on ice for 10–20 min, followed by 10 min incubation in incubation buffer (50 mM Tris, 5 mM MgCl_2_, 2.5 mM EDTA, 3% FCS, pH 7.4) and subsequently air-dried. Radiotracer or radiotracer (3–5 nM) with blocker solutions were added to the slides until well covered and (co-)incubated for 30 min in a humidified chamber at RT. The radiotracer or radiotracer with blocker solutions were decanted and the slides were washed 2 × 2 min in wash buffer (50 mM Tris, 5 mM MgCl_2_, 2.5 mM EDTA, 1% FCS, 5% EtOH, pH 7.4), followed by a 2 × 5 s wash in distilled H_2_O on ice. The slides were dried at RT and incubated with a phosphor film with a 30 min exposure time. The film was imaged with a BAS5000 phosphor imager (Fujifilm Life Science, Cambridge, USA).

Semiquantification was conducted using the AIDA Image Analysis Software (Elysia Raytest, Liège, Belgium) and based on the background-corrected intensity/area to obtain the intensity per pixel for comparison between differently sized tissues. The significance of blocking was calculated based on the relative signal to the baseline.

### Cell knockdown studies

The study workflow is depicted in Supplementary Fig. S27.

Cell culture. PC-3 cells were obtained from DSMZ (ACC 465). The cells were cultured in F12-K Nutrient Mixture (Ham) media with GLUTAMAX (Gibco, 31765-027) supplemented with 10% FBS and Pen/Strep (Gibco) antibiotics. PC-3 cells were cultured under standard conditions at 37 °C and 5% CO_2_ and detached with Trypsin-EDTA 0.25% (Gibco) for subculture.

siRNA Transfection. On transfection day PC-3 cells were trypsinised and reverse transfected with siRNA. 100 nmol of siRNA was mixed with 1.5% Lipofectamine RNAiMAX (13778150, Invitrogen) in Opti-MEM medium (31985062, Invitrogen) and added into the culture wells with 1 × 10^5^ PC-3 cells for qPCR and 1 × 10^6^ PC-3 cells for cell dishes. The Opti-MEM medium was changed to maintenance medium 24 h post transfection. The cells were either harvested after 96 h to determine knockdown efficiency or subjected to cell uptake experiments with [^11^C]J147. The mix of *ATP5A1* siRNAs: GGA CAG AUC UUC UUG GAA A; UUU CCA AGA AGA UCU GUC C; GGA GAG UAC UUU AGA GAC A; The mix of *Ctrl* siRNAs: UGG UUU ACA UGU CGA CUA ATT; UGG UUU ACA UGU UUU CUG ATT; UGG UUU ACA UGU UUU CCU ATT

qPCR. 96 h after transfection control and transfected cells, cultivated in a 24-well plate (TPP), were harvested and qPCR was performed to determine knockdown efficiency. Total RNA was extracted using RNeasy Mini Kit (74106, Qiagen). 0.5–1 μg of the total RNA was reverse transcribed to cDNA using a reverse transcription kit (# 4368814, Applied Biosystems). A Sybr Green (Thermofisher) based RT-qPCR was performed on a ViiA7 (Applied Biosystems) and the relative gene expression was calculated by ΔΔCt method taking *RPL13a* as housekeeping control. The primer sequences of the genes of interest are:

*ATP5A1-2*F: ACA CAG GCT GGT GAT GTG TC

*ATP5A1-2*R: ACA TTA ATG GCA GGG CGG AT

*hs_RPL13a* F: GGACCGTGCGAGGTATGCT

*hs_RPL13a* R: ATGCCGTCAAACACCTTGAGA

Cell uptake studies. The cellular uptake of [^11^C]J147 was determined using WT PC-3 and *ATP5A* siRNA PC-3 cells. After siRNA transfection, 1 × 10^6^ PC-3 cells were seeded in cell dishes (22.1 cm^2^, TPP) allowing adhesion and growth for 94 to 96 h. Triplicates of control and transfected cells were incubated with [^11^C]J147 (10 MBq/22.1 cm^3^) at 37 °C with constant shaking (0.1 × *g*, 20 min). After incubation, the media was removed, and cells were rinsed twice with 5 ml PBS. The cells were trypsinised and the radioactivity was measured in a γ-counter (Wizard, PerkinElmer). To correct for physical decay and to calculate uptake of radioactive compounds in each sample as a fraction of the administered dose, aliquots of the administered dose were counted simultaneously.

Isolation of functional mouse liver mitochondria. The procedure to isolate functional liver mitochondria was performed according to the protocol described in Serdiuk et al.^41^. Detailed procedure can be found in Methods in the Supplementary Information.

ATP hydrolysis assay in isolated mitochondria. The experimental procedure for assessing ATP hydrolysis activity of isolated mitochondria was identical to that reported by Serdiuk et al.^41^. A detailed scheme can be found in Supplementary Fig. S28. Detailed procedure can be found in Methods in the Supplementary Information.

Protein extraction and Western blot. A magnetic bead was added to tissue samples. Samples were lysed into a bead mill homogeniser (Tissuelyser II, Qiagen) in 500 µL RIPA buffer (RO278, Sigma-Aldrich), supplemented with protease inhibitor cocktail (Roche, 1 tablet/10 mL), at 30 Hz for 1 min. Samples are placed on a horizontal shaker for 30 min at 4 °C. Lysates were cleared by centrifugation at 15,000 *g* for 20 min at 4 °C. Protein concentration of the supernatants was determined by Pierce BCA protein assay (Thermo Scientific). Equal amounts of protein (40 µg) were separated on Mini-PROTEAN TGX Stain-free gel (Bio-Rad, USA), transferred to Trans-blot turbo transfer pack membrane (Bio-Rad, USA) and probed for ATP5A1 (MA5-32609, Thermo Fisher, 1:2000 dilution) and β-actin (ab8227, abcam, 1:1000). Signal of the HRP-conjugated secondary antibody, goat anti-rabbit IgG (G21234, Invitrogen, 1:2500) was visualised by the Image Quant system (GE Healthcare Life Sciences). ATP5A1 band signal was quantified using ImageJ and normalised to the relative actin band intensity.

### In vivo PET imaging

Mice were anaesthetised by inhalation of 3–5% isoflurane in oxygen/air (1:1). Respiration rate and body temperature were maintained at ~60 min^−1^ and 37 °C by adjusting isoflurane dose and warm air stream, respectively. Sterile radiotracer solution of [^11^C]J147 (6–14 MBq, 0.68–8.8 nmol/kg) was administered via tail-vein injection and dynamic PET scan was run from 1 to 61 min post-injection with subsequent CT scan recorded on a Super Argus PET/CT scanner (Sedecal S.A., Madrid, Spain). The dynamic PET scan lasted 60 min.

PET data were reconstructed with 2D ordered-subsets expectation maximisation (2D-OSEM), applying random scatter correction and decay correction but no correction for attenuation and analyzed with PMOD software (PMOD Technologies, Zurich, Switzerland; version 4.5) with user-defined time frames with a voxel size of 0.3875 × 0.3875 × 0.755 mm^3^. PET/CT images were generated with PMOD software. Time activity curves (TACs) were calculated by PMOD with manually defined regions of BAT. The brain regions of interest (ROIs) were defined on an MRI T2 (W. Schiffer) template provided by PMOD. Results are presented as mean standardised uptake value (SUV), indicating the decay-corrected radioactivity per cm^3^ divided by the injected radioactivity per gram of body weight – assuming a tissue density of 1.0 g/cm^3^.

Additional representative videos of PET imaging of vehicle-treated and activated Balb/c mice can be found in Supplementary information (Supplementary Movie 1 and 2).

### Radiometabolite study

After the i.v. injection of [^11^C]J147 (20-26 MBq activity injected) in 11 weeks old female Balb/c mice (21–22 g), mice were sacrificed at 5 min (*n* = 1) and 30 min (*n* = 1) post-injection. Blood, interscapular BAT and visceral WAT depots were immediately extracted. After decapitation, blood was rapidly collected in a blood collection tube (BD Vacutainer, LH Lithium heparin) and slowly mixed. The blood samples were pipetted in a 2 mL Eppendorf and centrifuged at 5000 x g (Sigma, 3K30, Sartorius) for 3 min at 4 °C to separate the plasma. The supernatant was collected in a 1.5 mL Eppendorf and an equal volume of cold acetonitrile was added. The mixture was vortexed for 10 s and centrifuged at 5000 × *g* for 3 min at 4 °C for deproteinisation. The supernatant was aspirated with a syringe and filtered with a 0.45 μm filter unit (Whatman, SPARTAN 13/0.45 RC) into an HPLC vial. The mouse WAT and BAT depots were homogenised using polytron (PT 2100) in a cold acetonitrile/PBS (1/1, v/v) solution. The homogenate was centrifuged at 5000 × g for 5 min at 4 °C. The supernatant was collected with a syringe and filtered through a 0.45 μm filter unit (Whatman, SPARTAN 13/0.45 RC) into an HPLC vial.

Column switch. Supernatants (0.3–0.7 mL, max. 50% aqueous CH_3_CN, 24-97 kBq) were loaded onto a 2 mL HPLC injector loop using a 1 mL syringe equipped with a square-cut needle. The sample was transferred onto a precolumn (ReproSil-Pur, 120 ODS-3, 10μ, 20 ×4.6 mm, Dr. A. Maisch HPLC GmbH, Germany) using 1% CH_3_CN in H_2_O (1 mL/min) for 4 min and subsequently back flushed onto a column (Luna 5 u C18 250 ×4.6 mm, Phenomenex Inc., Germany) for 1.5 min using 75% CH_3_CN in 0.1% aq. H_3_PO_4_ (1 mL/min). The column was eluted using a gradient of 75–90% CH_3_CN in 0.1% aq. H_3_PO_4_ over the course of 20 min (1 mL/min). Both column effluents were monitored through a UV detector (220 nm, diode array detector L-2450, Hitachi High-Technologies, Japan) and a radio-flow detector (FlowStar, LB 513, Berthold Technologies GmbH & Co.KG, Germany). All radioactivity data were corrected for physical decay and integrated. Standard of [^11^C]J147 injected into the column switch had a retention time of 15.5 min. (Supplementary Fig. S7) The results were analysed using the EZ Chrome Elite Software Package (Version 3.3.1, Agilent Technologies Inc., United States). Methods for the radioTLC can be found in the Supplementary information.

### Biodistribution study

Twelve female and twelve male Balb/c mice (6 vehicle-treated and 6 activated with β_3_-adrenergic agonist CL316,243) were administered [^11^C]J147 (6–14 MBq, 0.68–8.8 nmol/kg) or [^18^F]FDG (8–12 MBq) via tail-vein injection and euthanised by decapitation under isoflurane anaesthesia at 70 min post-injection. Organs were dissected and weighed, and radioactivity was measured in a γ-counter (Wizard, PerkinElmer). BAT and WAT depots sampling is explained and shown in Supplementary Figs. S29 and S30. To correct for physical decay and to calculate uptake of radioactive compounds in each sample as a fraction of the injected dose, aliquots of the injected dose were counted simultaneously. The activity in the interested organs was quantified as the percentage injected dose per gram tissue (% ID/g) followed by further correction as tissue-to-blood ratios (Fig. 6; Supplementary Table S1 and Table S2; also for awake animals Supplementary Tables S3 and S4).

### Statistics

All statistics were calculated with Graphpad Prism. (Multiple) unpaired Student’s *t* tests with Welch correction and correction for multiple comparisons using Holm-Šídák method, if applicable, were performed to compare only two groups or one-way ANOVA with post-hoc correction (Tukey) to compare more than two groups. Two-way ANOVA was used when comparing not only vehicle versus activated groups but also male versus female. *P* < 0.05 were considered statistically significant.

### Reporting summary

Further information on research design is available in the Nature Portfolio Reporting Summary linked to this article.

## Supplementary information

Supplementary Information

Description Of Additional Supplementary File

Supplementary Movie 1

Supplementary Movie 2

Reporting Summary

Transparent Peer Review file

## Source data

Source data

## Data Availability

All data supporting the results of this study can be found in the article, Supplementary Information, and Source Data files. Source data are provided as a Source Data file. Source data are provided with this paper.
