# Supplementary material for: ATP synthase is a promising target for identifying activated and non-activated adipose tissues
Source: Nat Commun. 2026 Apr 15;17:5233. doi: 10.1038/s41467-026-71343-w (PMC13260929; doi:10.1038/s41467-026-71343-w)
Supplement: Supplementary file 1 — Supplementary Information [file 41467_2026_71343_MOESM1_ESM.pdf]

Supplementary Information

ATP Synthase is a Promising Target for Identifying Activated and Non-activated Adipose Tissues

Caitlin V.M.L. Jie[1*], Aro Delparente[1*], Tongtong Wang[2], Lisa Reichert[1], Petra Krajnovic[1], Marc Schläppi[1], Lukas Reininger[1], Laura T. L. Brandt[3], Claudia Keller[1], Julien Orts[4,5], Roland Riek[4], Stefanie D. Krämer[1], Markus Stoffel[3], Christian Wolfrum[2], Roger Schibli[1,6], Linjing Mu[1ǂ]

[1] Institute of Pharmaceutical Sciences, Department of Chemistry and Applied Biosciences, ETH Zürich, CH-8093 Zürich, Switzerland

[2] Laboratory of Translational Nutrition Biology, Department of Health Sciences and Technology, ETH Zürich, 8603 Schwerzenbach, Switzerland.

[3] Institute for Molecular Health Sciences, Department of Biology, ETH Zürich, 8093 Zürich, Switzerland

[4] Institute for Molecular Physical Sciences, Department of Chemistry and Applied Biosciences, ETH Zürich, 8093 Zürich, Switzerland.

[5] Department of Pharmaceutical Sciences, University of Vienna, Josef-Holaubek-Platz 2, 1090 Vienna, Austria

[6] Center for Radiopharmaceutical Sciences, PSI Center for Life Sciences, 5232, Villigen-PSI, Switzerland

*These two authors contributed equally

ǂCorresponding author: linjing.mu@pharma.ethz.ch

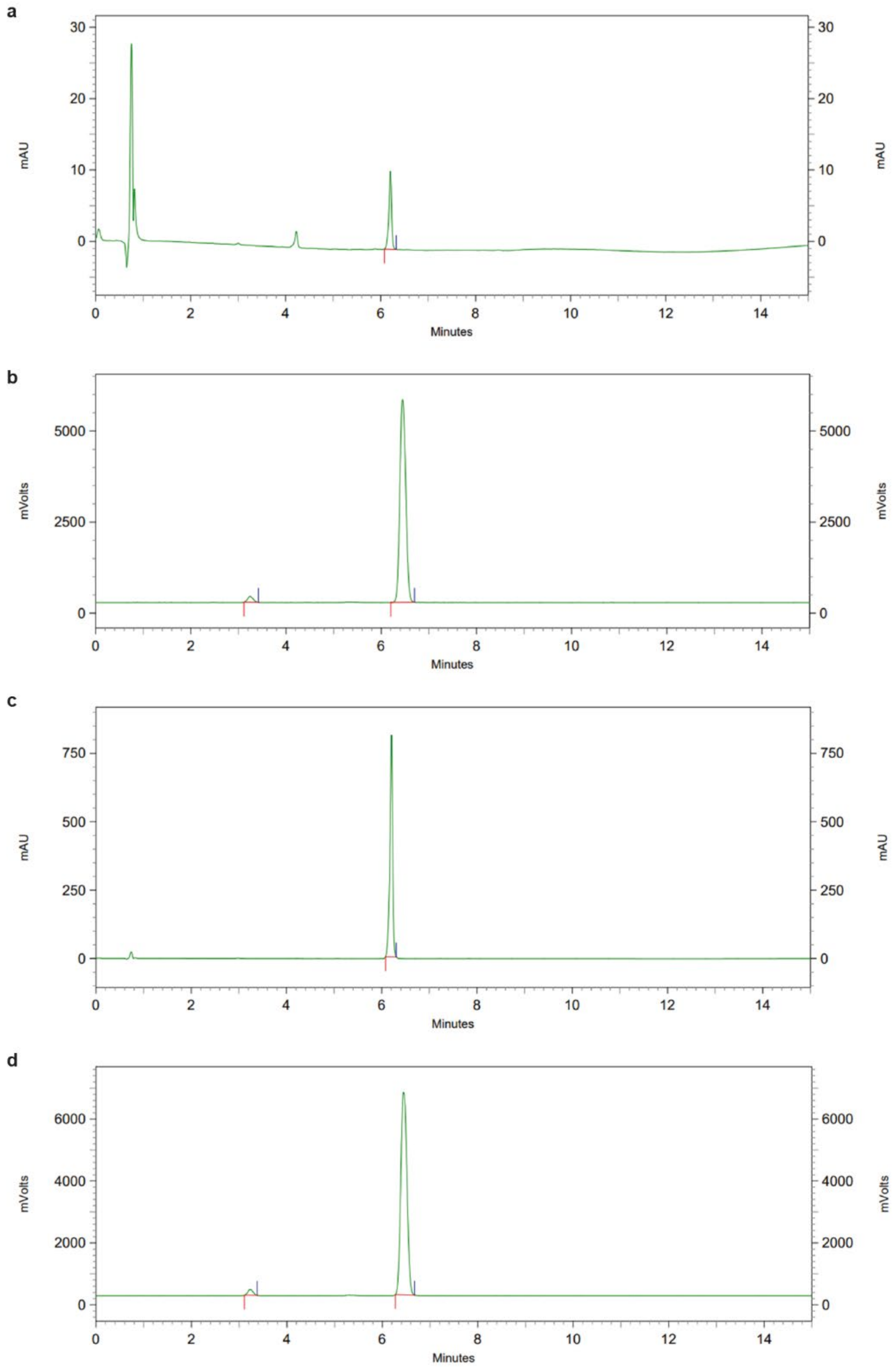

Fig. S1. HPLC chromatograms of [^{11}C]J147: UV in mAU at 254 nm (**a**) and radioactivity in mVolts (**b**). Co-injection of J147 with [^{11}C]J147: UV in mAU at 254 nm (**c**) and radioactivity in mVolts (**d**).

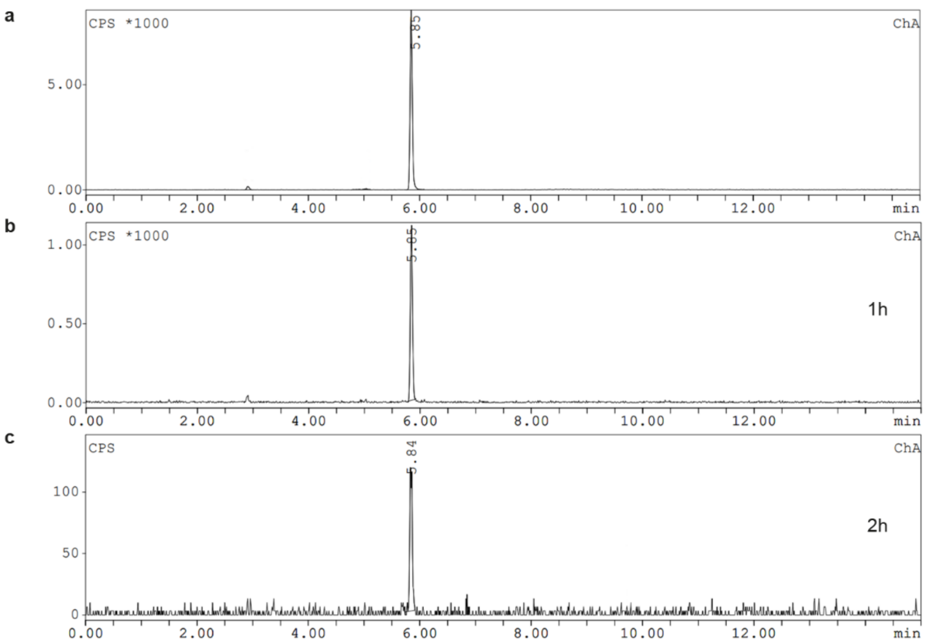

Fig. S2. Radiotracer stability of [^{11}C]J147 (RT: 5.8 min) in formulation (5% EtOH, 25% PEG300, 70% PBS, pH 7.4) immediately after production (**a**), after 1 hour (**b**), and after 2 hours (**c**). Radioactivity is depicted in counts per seconds (CPS).

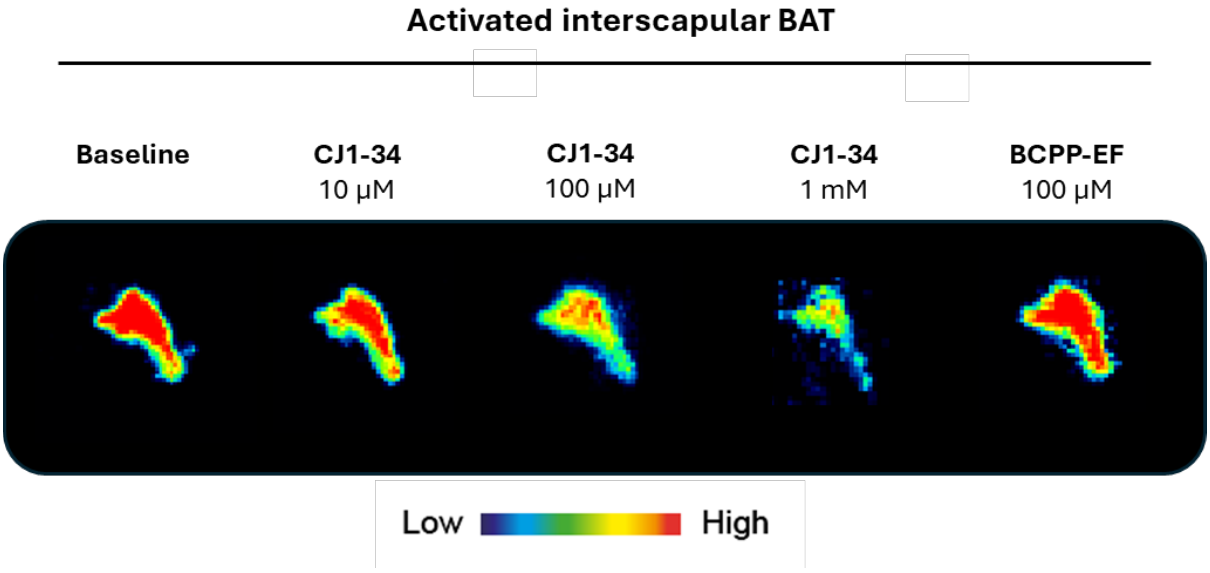

Fig. S3. Representative *in vitro* autoradiograms of [^{11}C]J147 (3-5 nM) obtained from CL316,243-treated Balb/c mice interscapular BAT sections under baseline condition, after addition of different concentration of CJ1-34 (10 µM, n = 6; 100 µM, n = 6 and 1 mM, n = 4, a compound targeting the ATP synthase) and after addition of 100 µM BCPP-EF (n =6, a compound targeting specifically the mitochondrial complex I).

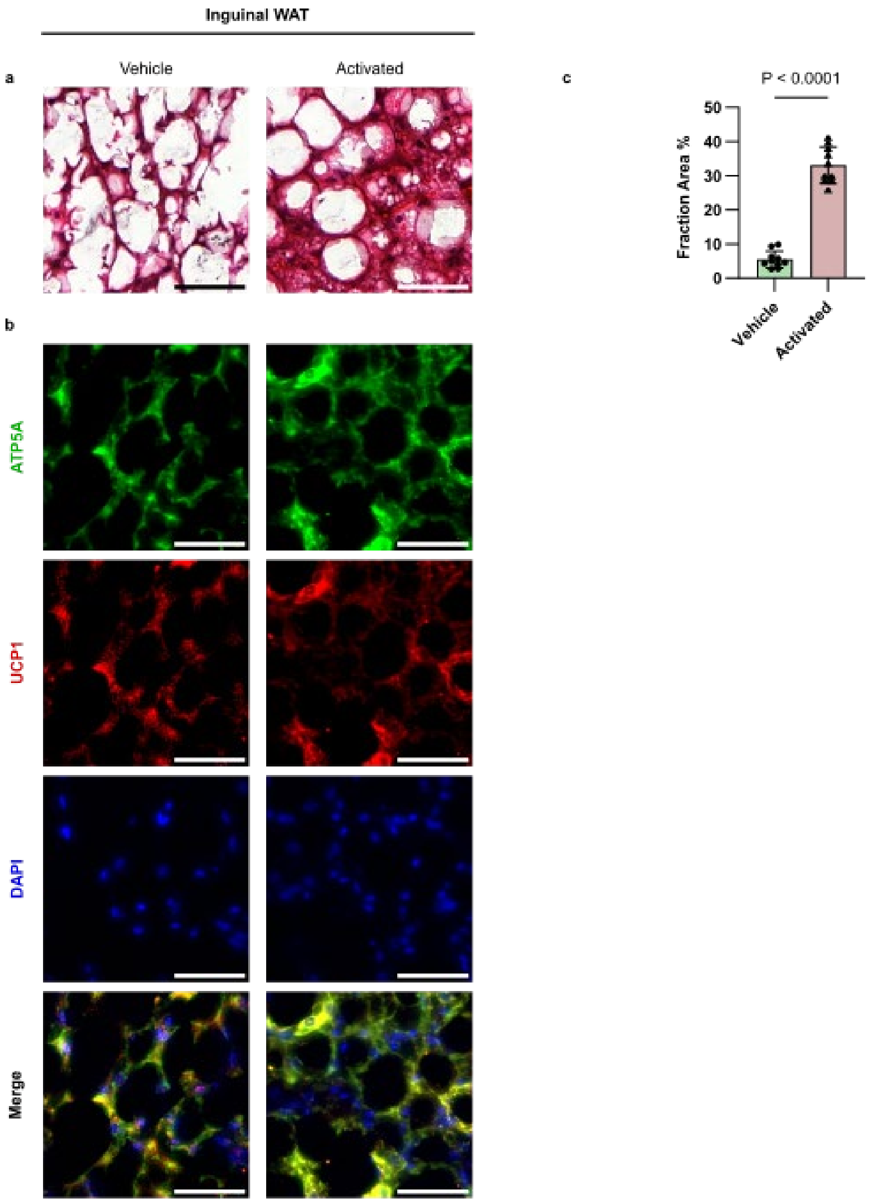

Fig. S4. **a,** H&E staining (scale bar 100 µm) of inguinal WAT, either vehicle-treated or activated with CL316,243 (1 mg/kg, i.p., 24 h and 1 h). **b,** Immunofluorescence staining of ATP5A (green), UCP1 (red), DAPI (blue) and merged (yellow) in vehicle-treated or CL316,243-treated Balb/c mice. Scale bar, 100 µm. **c**, Semiquantification of the immunofluorescence images of ATP5A in vehicle-treated or activated with CL316,243 inguinal WAT (n = 10 images per condition, $p < 0.0001$) depicted in fraction area percentage. n = 2 mice per group. Data are represented as mean ± s.d. Two-tailed Student's *t*-test with Welch correction (**c**).

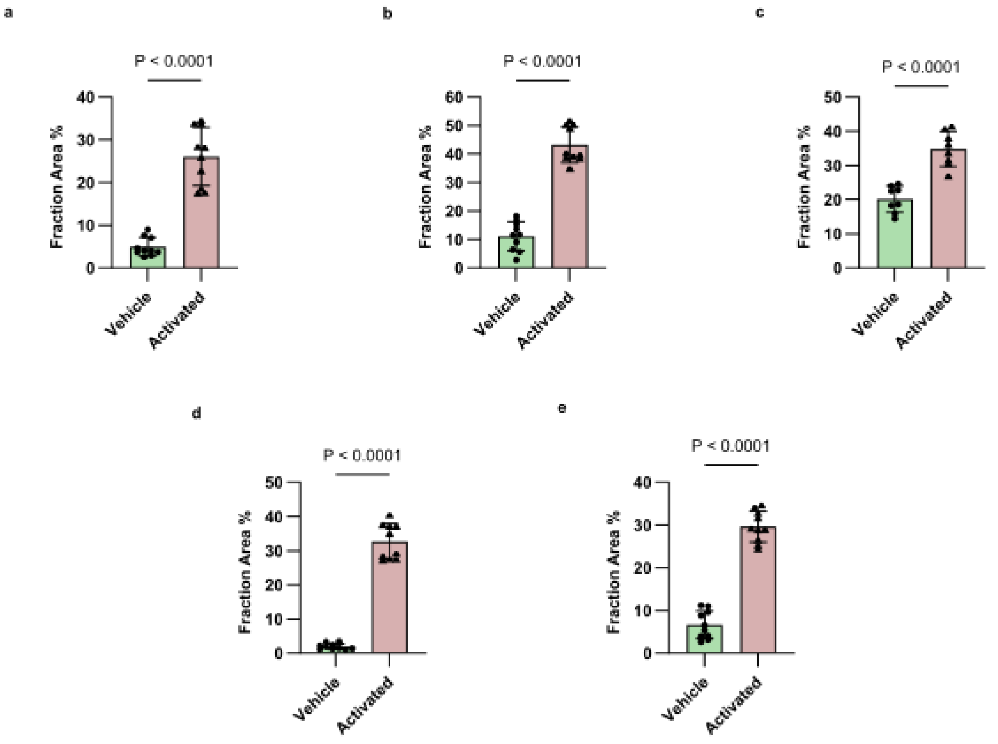

Fig. S5. **a-e**, Semiquantification of the immunofluorescence images of UCP1, either vehicle-treated (green) or activated with CL316,243 (red, 1 mg/kg, i.p., 24 h and 1 h) in interscapular BAT (**a,** n = 10 images for both groups, $p < 0.0001$), infrascapular BAT (**b,** n = 10 images for both groups, $p < 0.0001$), axillary BAT (**c,** n = 8 images for both groups, $p < 0.0001$), visceral WAT (**d,** n = 10 images for both groups, $p < 0.0001$), and inguinal WAT (**e,** n = 10 images for both groups, $p < 0.0001$) depicted in fraction area percentage. n = 2 mice per group. Data are represented as mean ± s.d. Two-tailed Student's *t*-test with Welch correction.

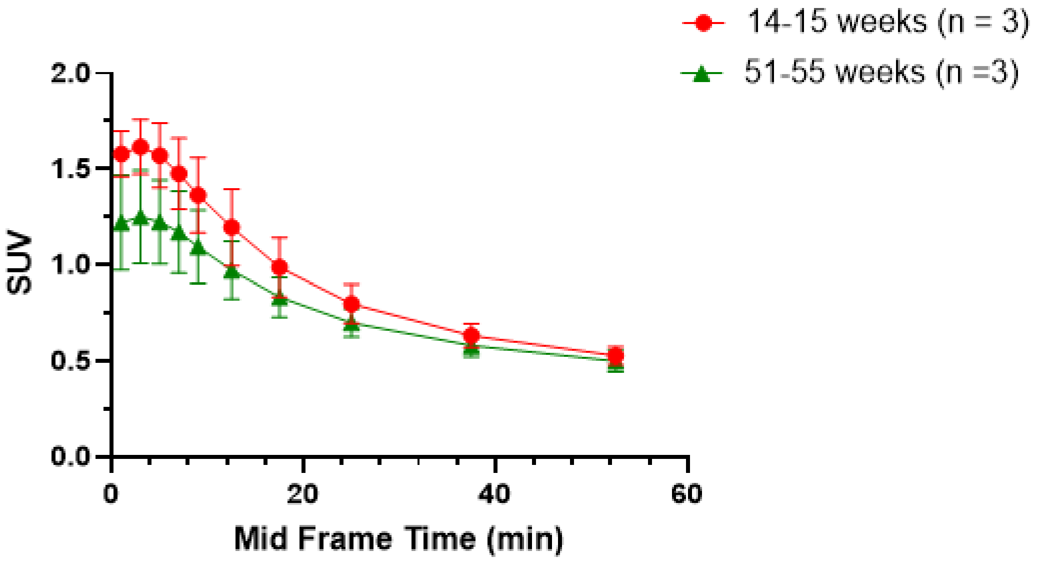

Fig. S6. Time activity curves (TACs) of [^{11}C]J147 in the whole brain in 14-15 weeks old (n=3 mice) and 51-55 weeks old (n=3 mice) C57BL/6;C3H mice.

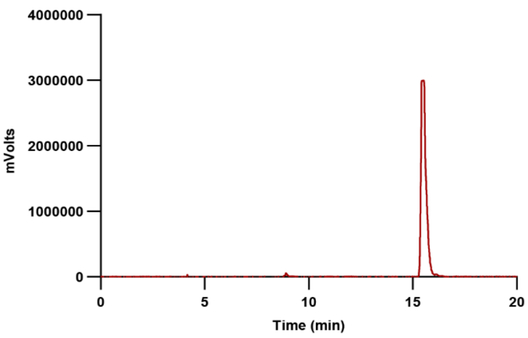

Fig. S7. Standard of [^{11}C]J147 injected into the column switch. RT: 15.5 min.

Radiometabolites study – RadioTLC. The obtained supernatants were analyzed by means of radio thin layer chromatography (radioTLC). Aliquots of the samples (2-5 µL) were deposited on TLC silica gel (60 F_{254} plates, Merck, Italy) and placed in a chamber with 40% acetone in hexanes mobile phase. After elution, the plate was dried and analysed using a bio-imaging analyser (Fujifilm BAS-2500, Raytek Scientific Ltd., United Kingdom). The results were analysed using the Aida Image Analyzer software package (version 450, Elysia-raytest GmbH, Germany).

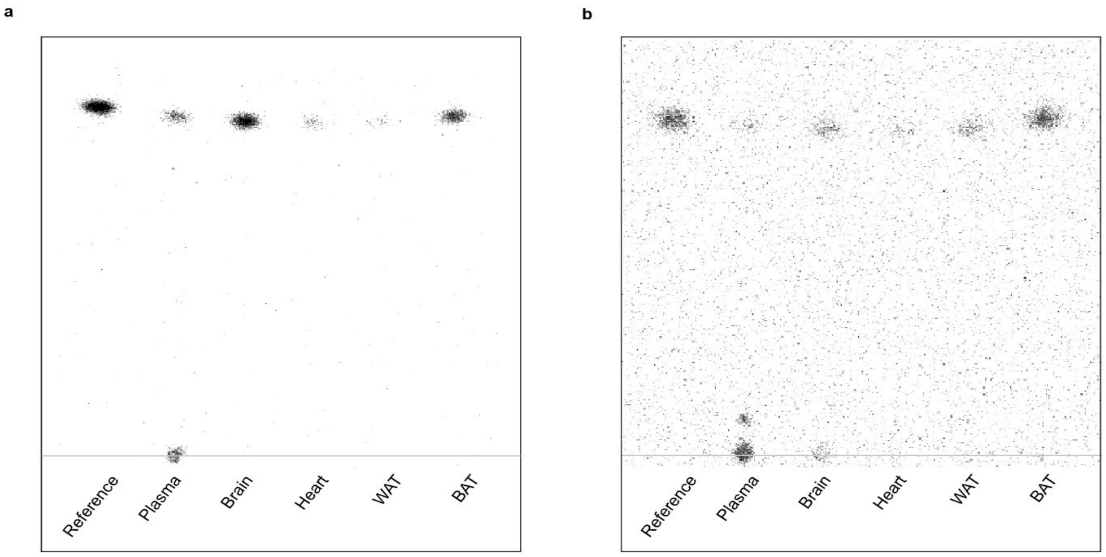

Fig. S8. RadioTLC of the same samples as utilised in the column switch. **a,** 5 min post injection time point. **b**, 30 min post injection time point.

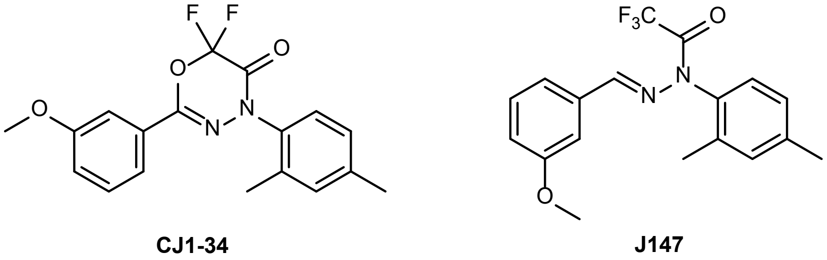

Fig. S9. Chemical structures of CJ1-34 and J147.

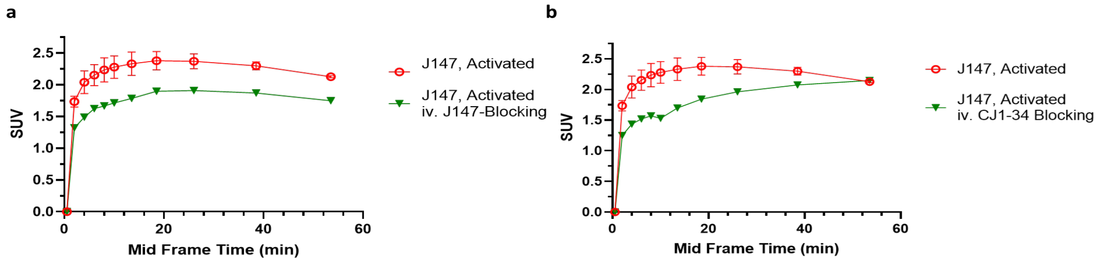

Fig. S10. Time-activity curves (TACs) of interscapular BAT from PET studies in mice. TACs represent accumulation of [^{11}C]J147 in infrascapular BAT as a function of time for baseline (red, n=3 mice) and blocking scan with J147 (green, n=1 mice, iv. 4 mg/kg) (a) or CJ1-34 (green, n=1 mice, iv. 4 mg/kg) (b) pretreatment 3 min before radiotracer injection.

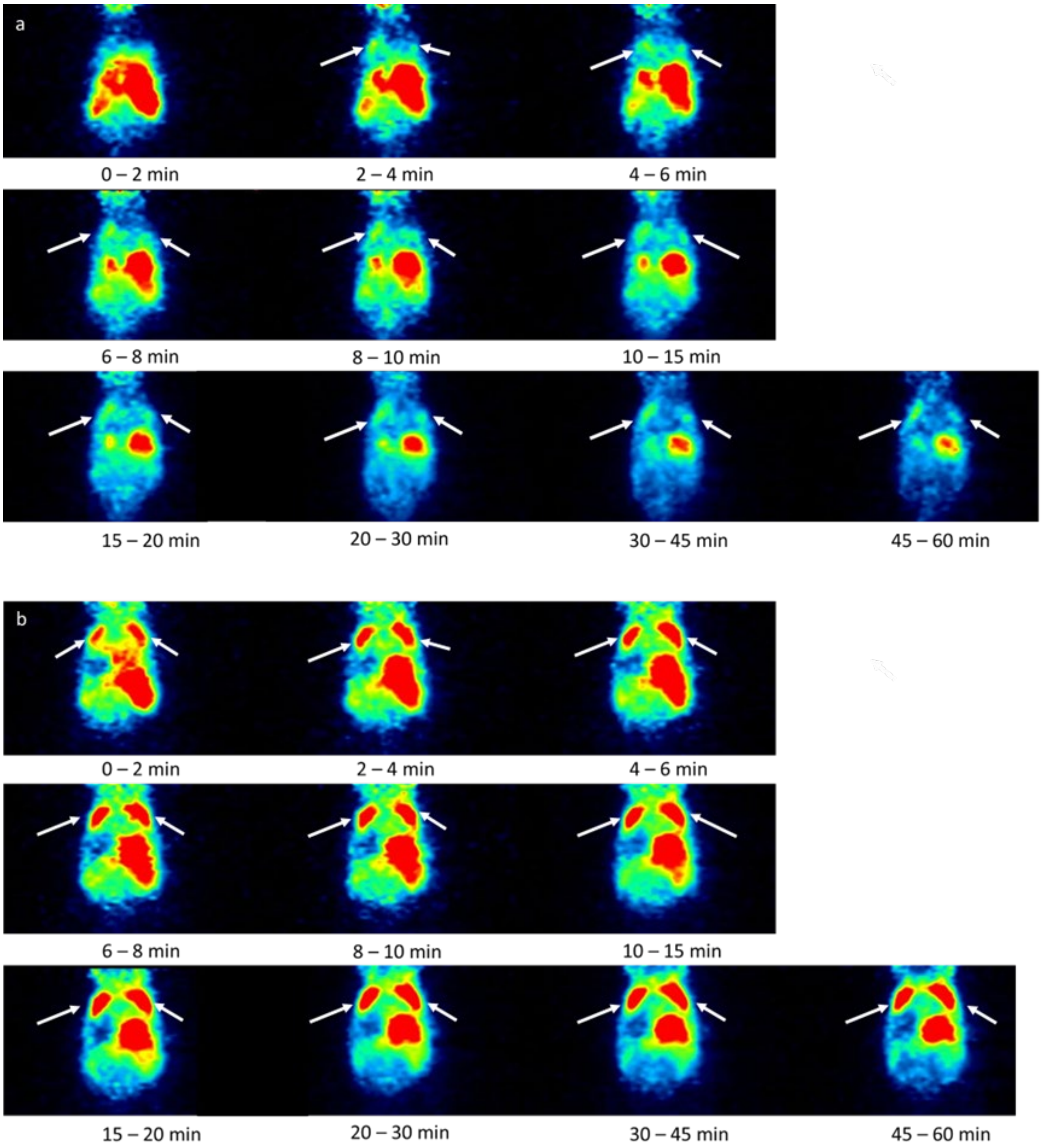

Figure S11. Representative PET images of sagittal mouse body sections at different time frames after intravenous injection of [^{11}C]J147 in vehicle-treated mice (**a**) and [^{11}C]J147 in CL316,243-treated mice (**b**). White arrows indicate interscapular BAT depots over time. SUV: 0-2.5.

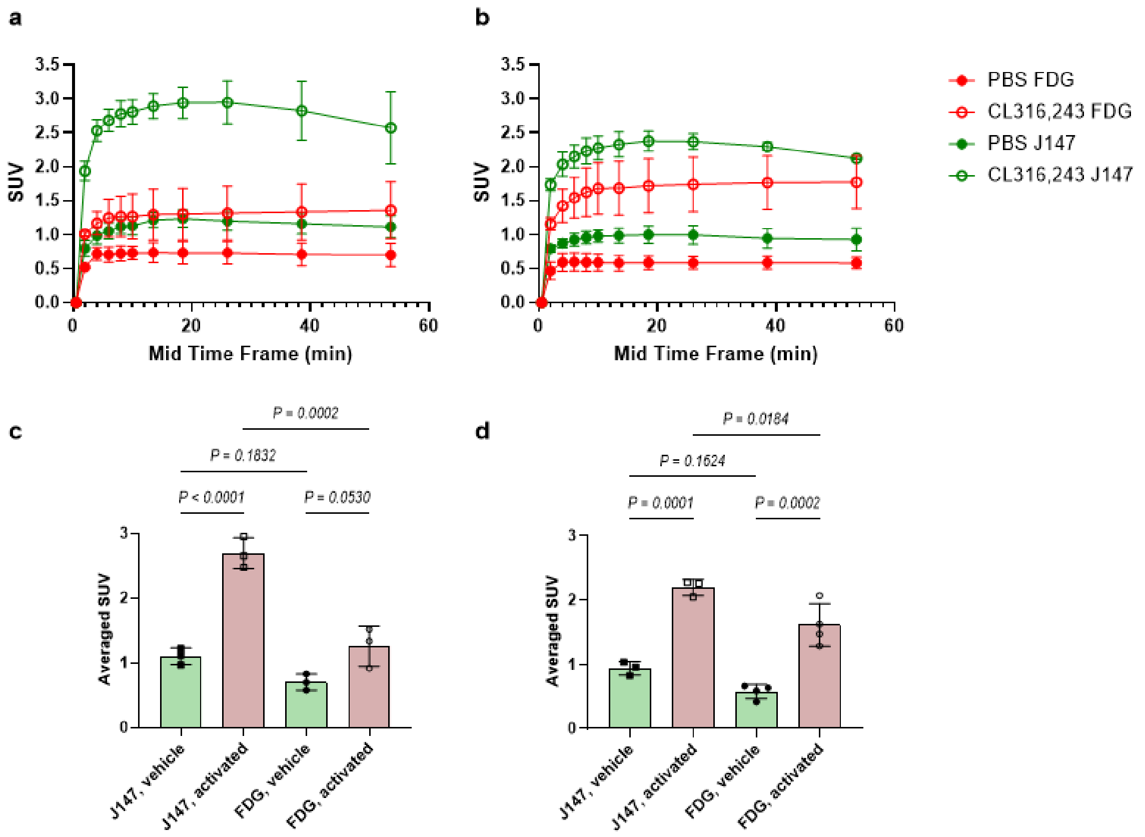

Fig. S12. *In vivo* PET imaging study with [11C]J147 and [18F]FDG in vehicle (also named PBS)-treated or CL316,243-treated mice. Time activity curves of [11C]J147 (n=3 mice) and [18F]FDG (n=3 mice) in interscapular BAT in vehicle-treated and CL316,243-treated females (**a**). Time activity curves of [11C]J147 (n=3 mice) and [18F]FDG (n=4 mice) in interscapular BAT in vehicle-treated and CL316,243-treated males (**b**). Averaged SUVs of the vehicle-treated and CL316,243-treated female (**c**). CL316,243 treatment significantly increased interscapular BAT SUV for [11C]J147 (n = 3, $p < 0.0001$). Interscapular BAT averaged SUV was significantly higher for [11C]J147 than for [18F]FDG in the CL316,243-treated groups ($p = 0.0002$). Averaged SUVs of the vehicle-treated and CL316,243-treated male (**d**). CL316,243 treatment significantly increased interscapular BAT SUV for [11C]J147 (n = 3, $p = 0.0001$) and [18F]FDG (n = 4, $p = 0.0002$). Interscapular BAT averaged SUV was significantly higher for [11C]J147 than for [18F]FDG in the CL316,243-treated groups ($p = 0.0184$). Data are represented as mean ± s.d. One-way ANOVA with Turkey's post-hoc correction for multiple comparisons.

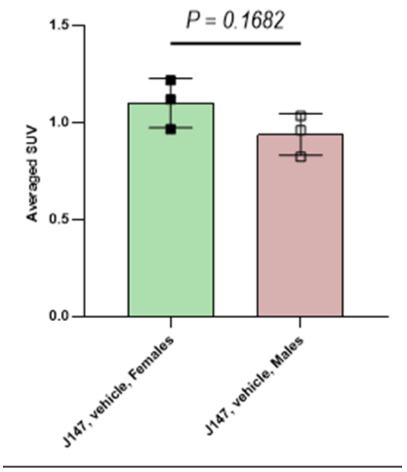

Figure S13. Averaged SUVs of [11C]J147 for the interscapular BAT of the vehicle-treated female (n = 3 mice) and male (n = 3) groups. Data are represented as mean ± s.d. Two-tailed Student's *t*-tests with Welch correction.

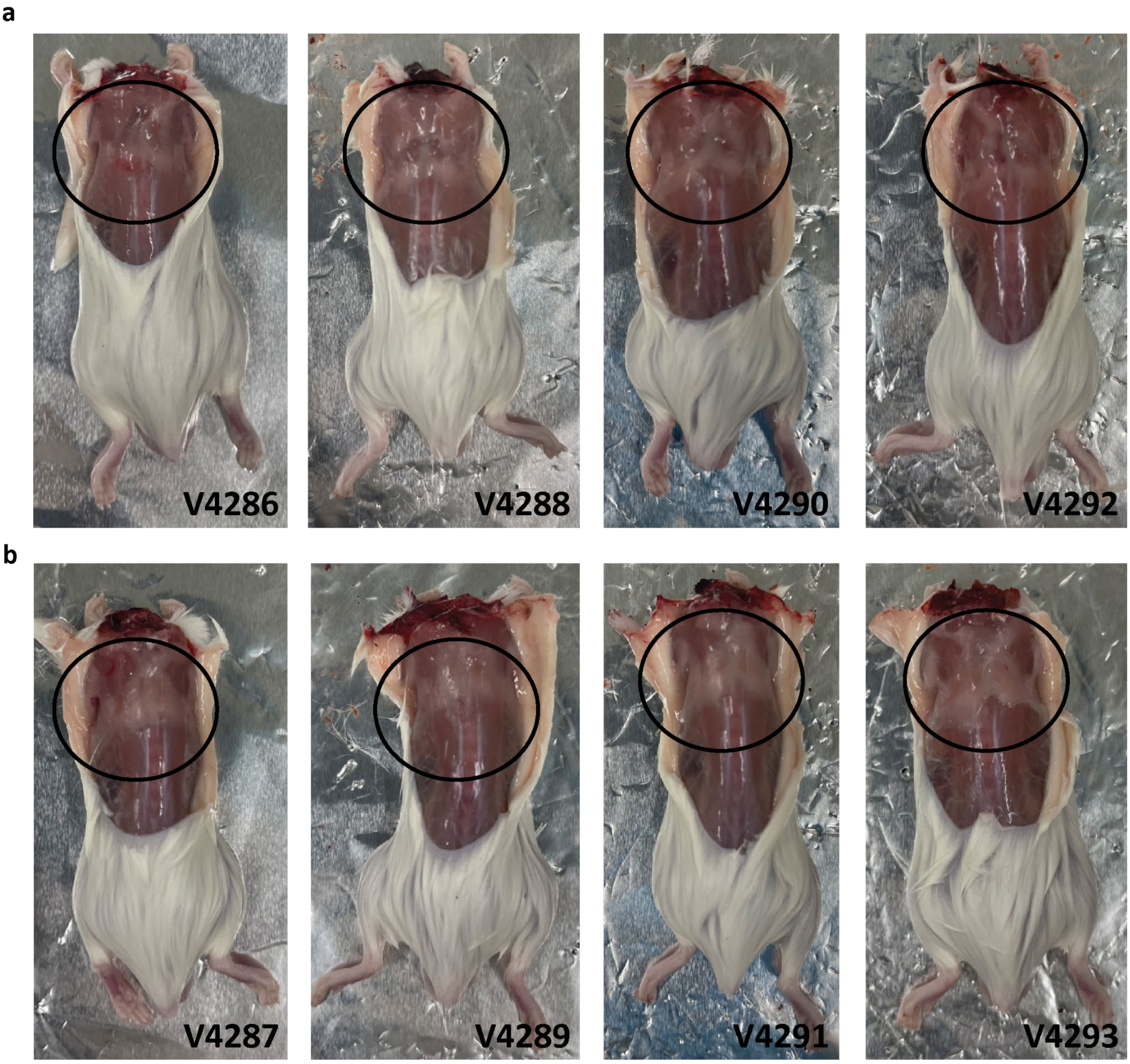

Fig. S14. Pictures show the location and size of the classical (interscapular) brown adipose tissues (indicated by the black circle) located on the back of the animals. (**a**) Panel shows Balb/c mice injected with CL316,243 after decapitation, skin is cut and removed to expose the adipose tissues below. (**b**) Panel show Balb/c mice injected with vehicle (PBS). No visible differences were observed.

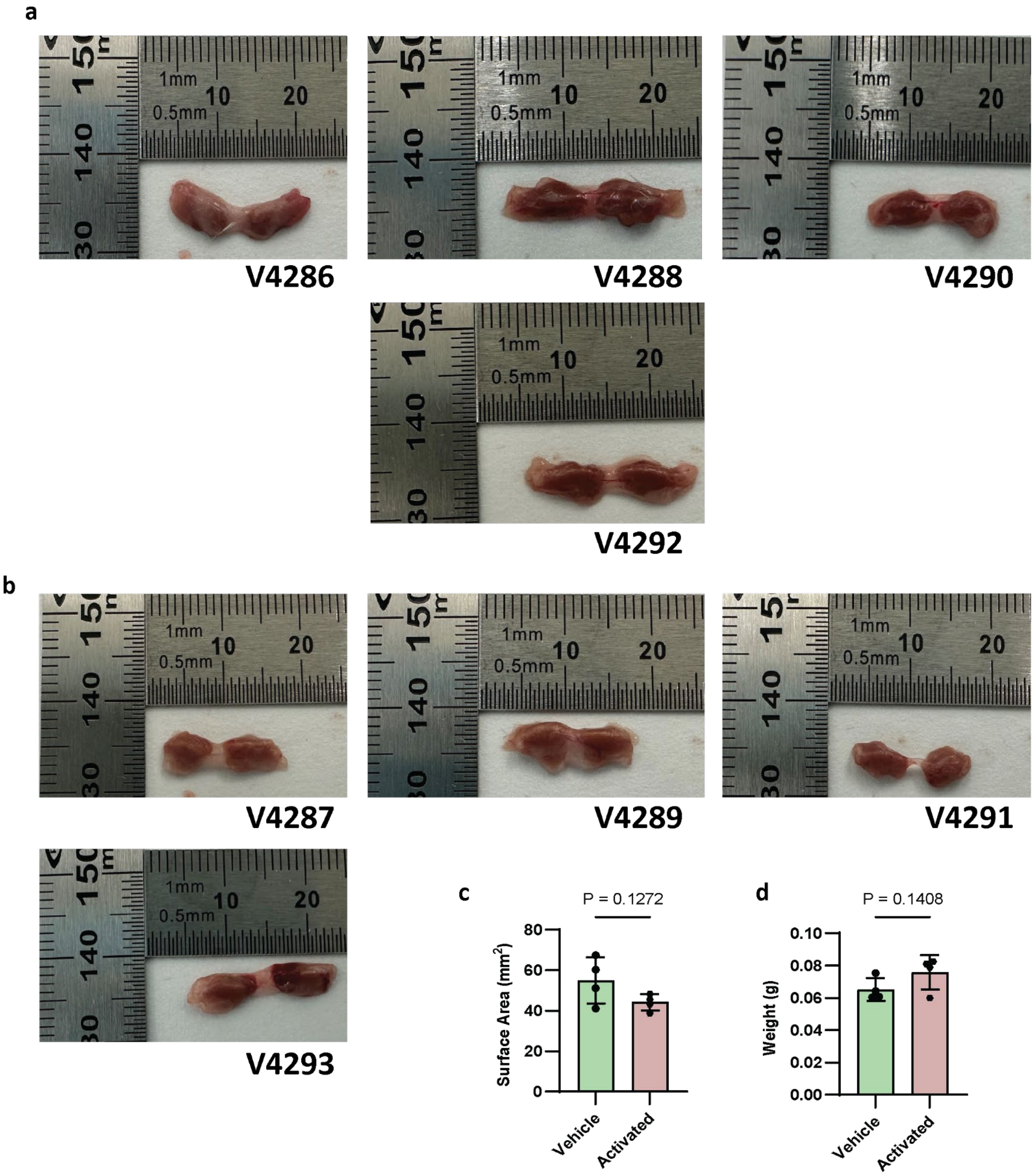

Fig. S15. Classical brown fat depots from mice injected with CL316,243 (**a**) and vehicle (**b**) were dissected and measured. (**c**) No significant difference in classical brown fat adipose tissue surface area was detected between treatment groups. (**d**) No significant difference in classical brown adipose depot weight was observed between treatment groups. Data are presented as mean ± s.d. Two-tailed Student's *t*-test with Welch correction (**c**,**d**).

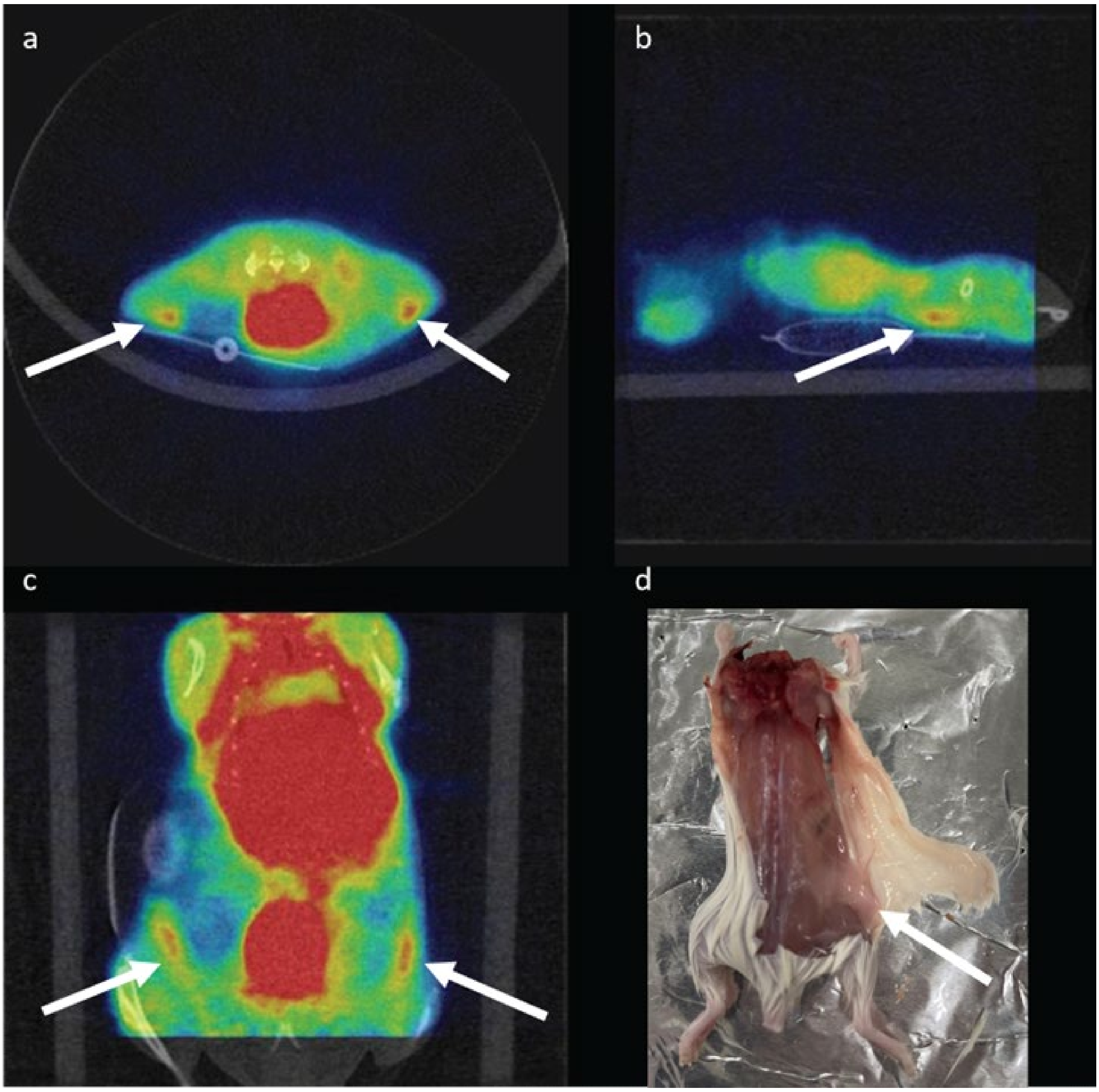

Figure S16. *In vivo* PET imaging study with [^{11}C]J147 in vehicle-treated mice. Representative averaged PET/CT images (time frame: 0-60 min) with [^{11}C]J147 of vehicle-treated mice. Representative images depicting transverse (**a**), sagittal (**b**) and coronal (**c**) planes where inguinal WAT is indicated with red arrows. The image (**d**) shows the anatomical location of the inguinal WAT of mice during dissection, which are visibly located in the lower abdominal region, overlying the thigh region.

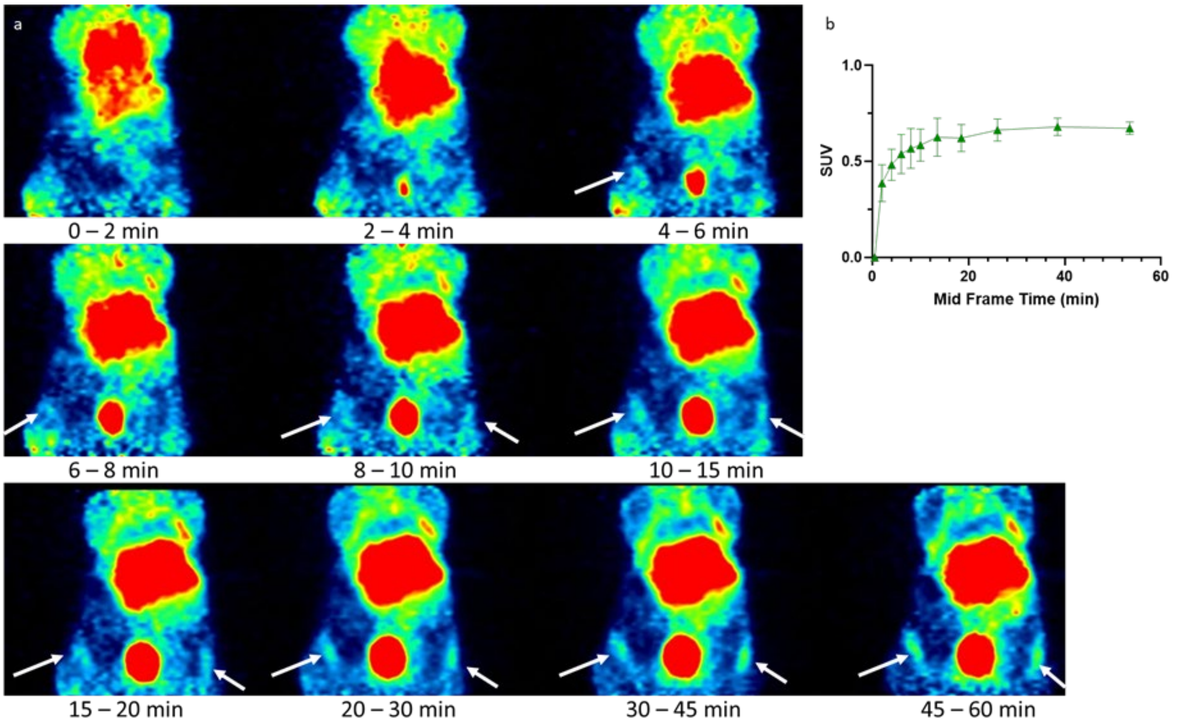

Figure S17. *In vivo* uptake of [^{11}C]J147 in vehicle-treated mice. White arrows indicate inguinal WAT in the sequential frames of coronal PET images (**a**). Time activity curve of [^{11}C]J147 of left inguinal WAT from vehicle-treated mice (n=3 mice) (**b**). Data are represented as mean ± s.d. Signal quantification was performed exclusively on the left side of the body for the left inguinal WAT, as the right side was affected in some cases by signal spillover from the intestine or the bladder.

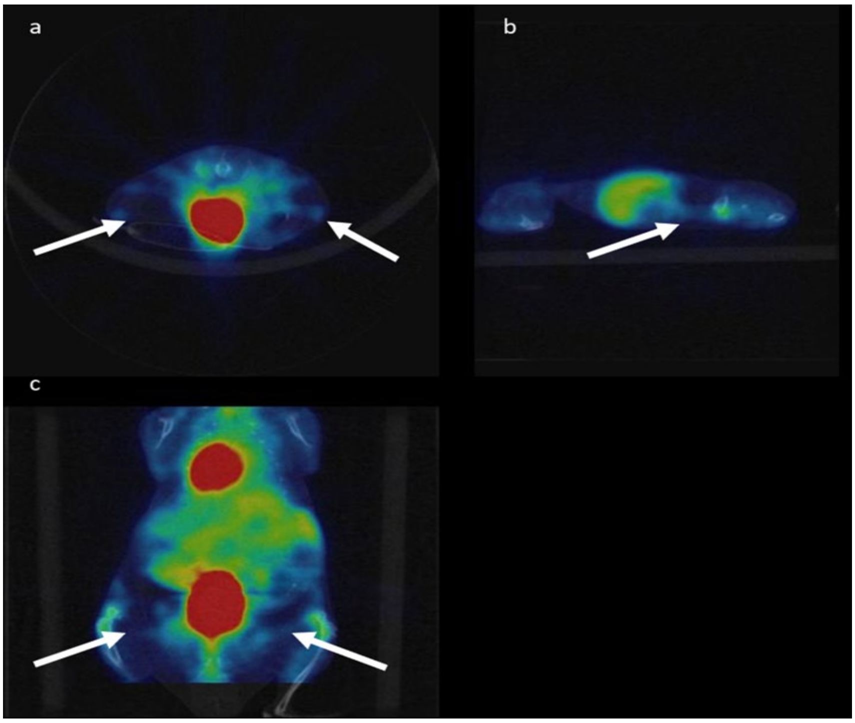

Figure S18. *In vivo* PET imaging study with [^{18}F]FDG in vehicle-treated mice. Representative averaged PET/CT images (time frame: 0-60 min) with [^{18}F]FDG of vehicle-treated mice. Representative images depicting transverse (**a**), sagittal (**b**) and coronal (**c**) planes where inguinal WAT is not visible.

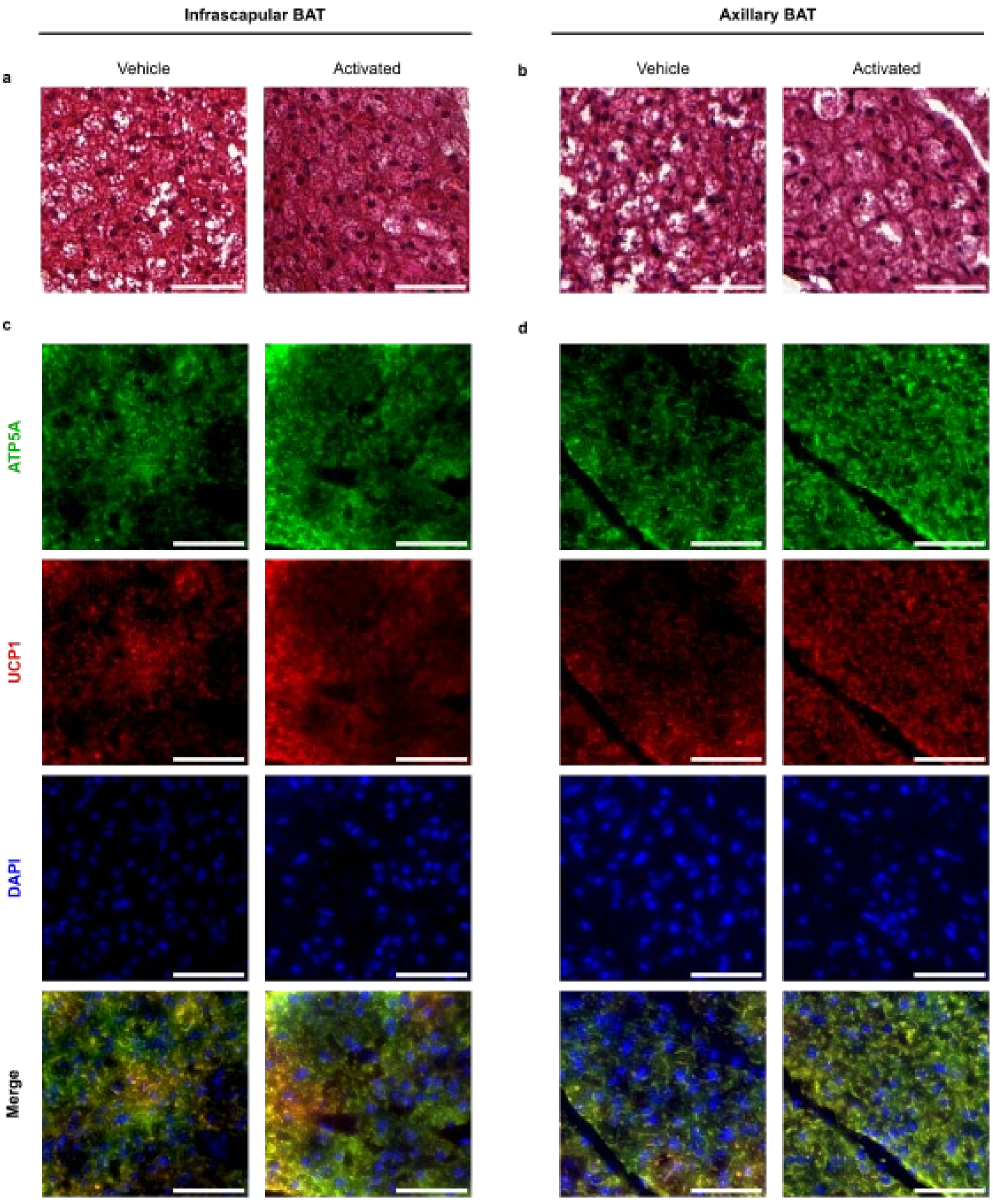

Figure S19. **a,b** H&E staining (scale bar 100 µm) of (**a**) infrascapular BAT, (**b**) axillary BAT, either vehicle-treated or activated with CL316,243 (1 mg/kg, i.p., 24 h and 1 h). **c,d,** Immunofluorescence staining of ATP5A (green), UCP1 (red), DAPI (blue) and merged (yellow) in vehicle-treated or CL316,243-treated (**c**) infrascapular BAT and (**d**) axillary BAT of Balb/c mice. Scale bar, 100 µm.

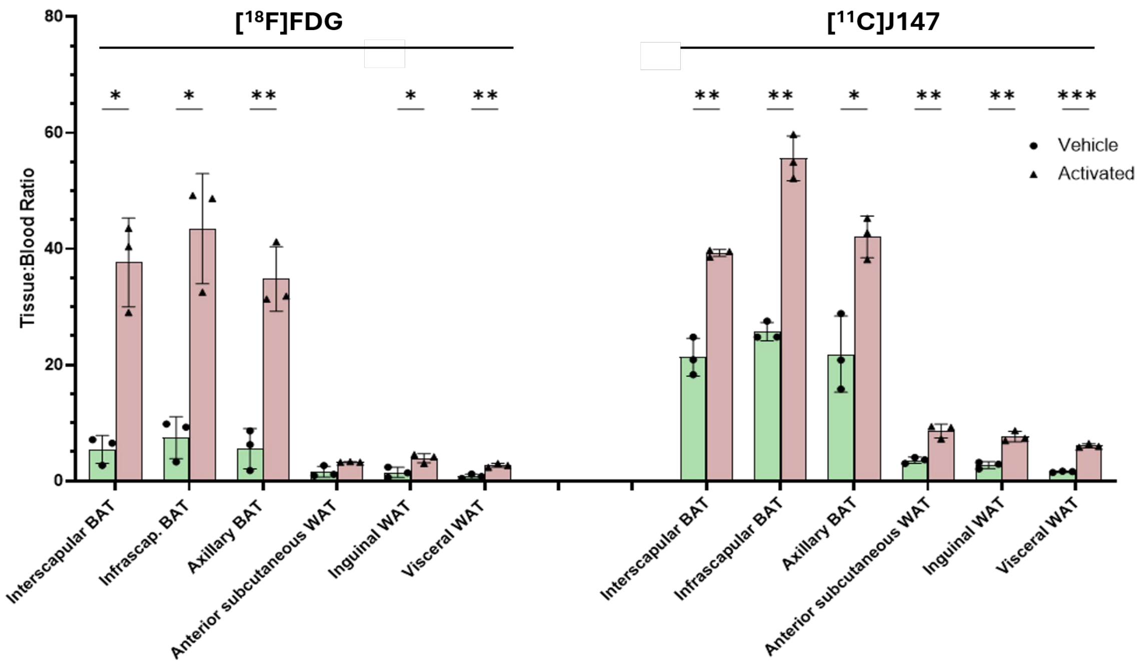

Figure S20. Biodistribution of [^{18}F]FDG in vehicle-treated (n = 3 mice) or CL316,243-treated Balb/c mice (n = 3 mice) and biodistribution of [^{11}C]J147 in vehicle-treated (n = 3 mice) or CL316,243-treated Balb/c mice (n = 3 mice) reported as tissue-to-blood ratio (mean ± SD). Animals were injected awake for both tracers and sacrificed at 15 min after iv injection. Multiple unpaired t-tests with Welch correction and correction for multiple comparisons using Holm-Šídák method.

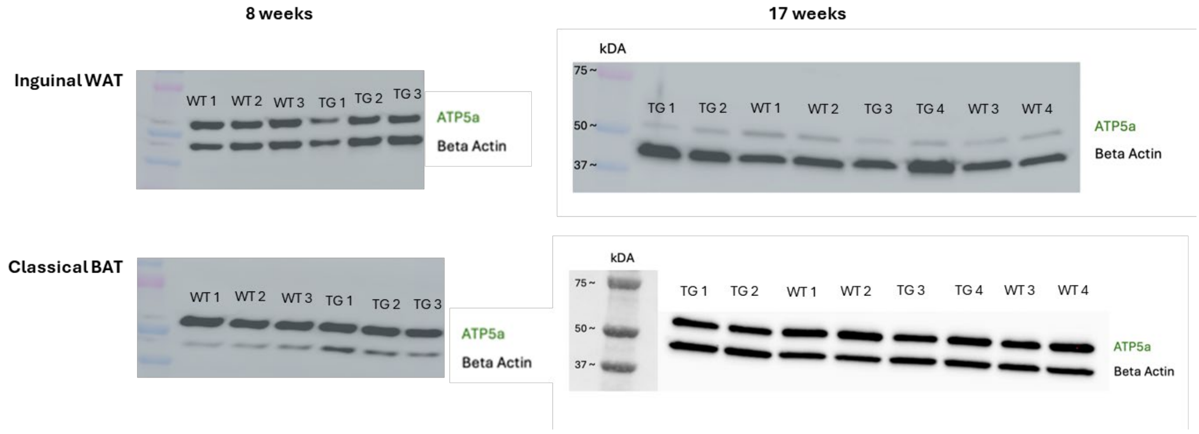

Fig. S21. Western Blot of ATP5A expression in inguinal WAT and interscapular BAT in 8 weeks old (n = 3 mice per group) and 17 weeks old (n = 4 mice per group) STAM and control mice.

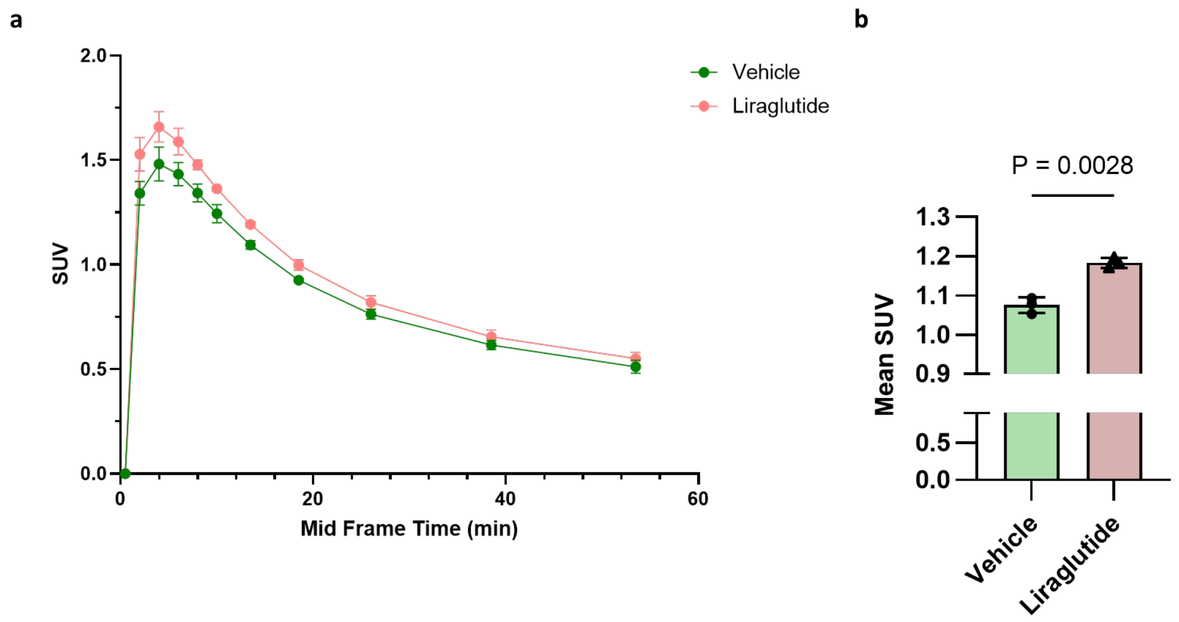

Figure S22. Time activity curve of [11C]J147 in vehicle-treated (n = 3) or liraglutide-treated Balb/c mice (n = 3) for the brain (**a**). Averaged SUVs of [11C]J147 for the brain of the vehicle-treated and liraglutide-treated mice groups (n =3 per group, p = 0.0028) (**b**). Data are represented as mean ± s.d. Two-tailed Student's t-tests with Welch correction.

Table S1. Biodistribution of [^{11}C]J147 with vehicle-treated or CL316,243-treated Balb/c mice.

Organ	Vehicle-treated group (%ID/g, n=6)		CL316,243-treated group (%ID/g, n=6)		*P* value[c]
	Average	SD	Average	SD	
Spleen	1.70	0.20	1.91	0.23	0.130
Liver	11.06	0.92	16.23	2.02	0.0002
Kidney	3.01	0.26	4.10	0.87	0.015
Lung	1.87	0.36	1.79	0.43	0.724
Bone	1,56	0.35	1.44	0.27	0.527
Heart	1.76	0.28	1.90	0.41	0.480
Whole brain	1.59	0.38	1.22	0.22	0.061
Gall bladder	50.18	12.95	30.39	13.03	0.025
Blood	1.60	0.20	1.21	0.23	0.011
Stomach[a]	2.05	0.21	1.57	0.09	0.001
Small intestine[b]	11.44	1.95	8.20	1.25	0.007
Urine	30.82	17.20	15.69	11.44	0.103
Muscle	1.18	0.32	1.36	0.37	0.386
Pancreas	4.08	0.72	4.82	0.50	0.068
Skin	1.46	0.31	2.04	0.39	0.017
Adrenal gland	4.57	1.26	9.81	0.51	0.0001
Interscapular BAT	5.93	2.33	17.56	2.16	0.00001
Infrascapular BAT	6.97	3.43	10.02	3.41	0.00001
Axillary BAT	5.38	1.37	15.52	4.95	0.0001
Anterior subcutaneous WAT	4.00	0.79	5.61	1.26	0.024
Inguinal WAT	3.43	0.78	5.03	0.92	0.009
Visceral WAT	1.69	0.46	3.18	0.45	0.0003

Ratio	Vehicle-treated group (Tissue:Blood Ratio, n=6)		CL316,243-treated group (Tissue:Blood Ratio, n=6)		*P* value[d]
Interscapular BAT/Blood	3.71	1.43	15.04	3.75	0.007
Infrascapular BAT/Blood	4.36	2.19	17.41	5.79	0.027
Axillary BAT/Blood	3.39	0.93	13.53	6.21	0.111
Anterior subcutaneous WAT/Blood	2.52	0.53	4.68	0.83	0.010
Inguinal WAT/Blood	2.18	0.57	4.30	1.32	0.111
Visceral WAT/Blood	1.06	0.27	2.66	0.70	0.026

WAT: white adipose tissue, BAT: brown adipose tissue, SD: standard deviation. [a] without stomach content, [b] with content. [c] Unpaired t-tests, two-sided, not corrected for multiple comparisons. [d] Multiple unpaired t-tests, two-sided with Welch correction and correction for multiple comparison using Holm-Šídák method.

Table S2. Biodistribution of [^{18}F]FDG with vehicle-treated or CL316,243-treated Balb/c mice.

Organ	Vehicle-treated group (%ID/g, n=7)		CL316,243-treated group (%ID/g, n=6)		*P* value[c]
	Average	SD	Average	SD	
Spleen	6.76	0.94	8.05	1.04	0.039
Liver	1.97	0.42	1.59	0.18	0.068
Kidney	19.939	3.01	17.23	3.91	0.284
Lung	7.59	0.74	7.69	1.67	0.896
Bone	2.75	0.58	3.27	0.67	0.165
Heart	99.84	24.24	80.18	34.91	0.257
Whole brain	5.98	0.65	5.35	0.98	0.196
Gall bladder	2.19	0.48	2.32	0.37	0.606
Blood	1.03	0.18	1.13	0.17	0.342
Stomach[a]	3.80	1.14	2.82	0.64	0.090
Small intestine[b]	7.35	2.06	5.18	0.81	0.035
Urine	132.11	47.72	151.51	60.26	0.530
Muscle	0.79	0.18	1.00	0.22	0.089
Pancreas	3.07	0.27	6.71	1.75	0.00020
Skin	1.33	0.37	1.78	0.52	0.091
Adrenal gland	8.82	5.16	17.80	6.13	0.015
Interscapular BAT	3.69	0.54	10.41	2.47	0.000021
Infrascapular BAT	3.64	0.65	6.03	1.36	0.003
Axillary BAT	4.11	1.68	8.54	4.89	0.045
Anterior subcutaneous WAT	1.35	0.52	4.78	2.22	0.004
Inguinal WAT	1.17	0.43	6.51	1.35	0.00000075
Visceral WAT	0.50	0.38	7.21	1.56	0.00000027

Ratio	Vehicle-treated group (Tissue:Blood Ratio, n≥6)		CL316,243-treated group (Tissue:Blood Ratio, n=6)		*P* value[d]
Interscapular BAT/Blood	3.66	0.81	9.37	2.24	0.018
Infrascapular BAT/Blood	3.72	1.14	5.50	1.61	0.486
Axillary BAT/Blood	4.01	1.57	7.61	4.60	0.747
Anterior subcutaneous WAT/Blood	1.31	0.34	4.24	1.76	0.132
Inguinal WAT/Blood	1.12	0.32	5.97	1.99	0.003
Visceral WAT/Blood	0.46	0.33	6.56	2.11	0.015

WAT: white adipose tissue, BAT: brown adipose tissue, SD: standard deviation. [a] without stomach content, [b] with content. [c] Unpaired t-tests, two-sided, not corrected for multiple comparisons. [d] Multiple unpaired t-tests, two-sided with Welch correction and correction for multiple comparison using Holm-Šídák method.

Table S3. Biodistribution of [^{11}C]J147 with vehicle-treated or CL316,243-treated Balb/c mice in awake animals at 15 min post-injection.

Organ	Vehicle-treated group (%ID/g, n=3)		CL316,243-treated group (%ID/g, n=3)		*P* value[c]
	Average	SD	Average	SD	
Spleen	2.51	0.45	2.76	0.63	0.596
Liver	12.78	3.05	21.51	14.72	0.371
Kidney	5.57	0.51	10.85	4.91	0.138
Lung	3.75	1.17	3.90	1.28	0.886
Bone	1,13	0.12	0.90	0.52	0.493
Heart	2.74	0.26	8.02	3.03	0.039
Whole brain	2.96	0.54	3.12	0.96	0.822
Gall bladder	45.26	24.77	21.95	22.51	0.294
Blood	1.26	0.20	0.80	0.08	0.020
Stomach[a]	3.03	0.94	2.20	0.24	0.214
Small intestine[b]	13.03	13.38	3.03	0.13	0.265
Urine	78.91	81.75	35.77	12.05	0.417
Muscle	1.85	0.07	2.31	0.52	0.200
Pancreas	4.72	0.59	4.81	0.38	0.823
Skin	1.87	0.39	1.97	0.37	0.775
Adrenal gland	13.40	0.74	14.98	2.03	0.273
Interscapular BAT	26.51	2.94	31.61	3.44	0.123
Infrascapular BAT	32.15	3.31	44.86	7.12	0.049
Axillary BAT	26.81	5.30	33.62	0.25	0.090
Anterior subcutaneous WAT	4.41	0.30	6.91	1.26	0.029
Inguinal WAT	3.36	0.71	6.14	0.90	0.014
Visceral WAT	1.96	0.29	4.85	0.38	0.0005

Ratio	Vehicle-treated group (Tissue:Blood Ratio, n=3)		CL316,243-treated group (Tissue:Blood Ratio, n=3)		*P* value[e]
Interscapular BAT/Blood	21.32	3.26	39.27	0.60	0.009
Infrascapular BAT/Blood	25.70	1.58	55.60	3.85	0.002
Axillary BAT/Blood	21.83	6.58	42.05	3.62	0.017
Anterior subcutaneous WAT/Blood	2.70	0.59	7.64	0.90	0.008
Inguinal WAT/Blood	1.56	0.11	6.04	0.33	0.002
Visceral WAT/Blood	10.77	1.21	18.58	0.88	0.001

WAT: white adipose tissue, BAT: brown adipose tissue, SD: standard deviation. [a] without stomach content, [b] with content. [c] Unpaired t-tests, two-sided, not corrected for multiple comparisons. [e] Multiple unpaired t-tests, two-sided with Welch correction.

Table S4. Biodistribution of [^{18}F]FDG with vehicle-treated or CL316,243-treated Balb/c mice in awake animals at 15 min post-injection. .

Organ	Vehicle-treated group (%ID/g, n=3)		CL316,243-treated group (%ID/g, n=3)		*P* value[c]
	Average	SD	Average	SD	
Spleen	2.41	0.98	1.99	0.14	0.503
Liver	2.61	1.32	1.39	0.14	0.186
Kidney	3.65	2.05	2.91	0.26	0.569
Lung	2.97	0.80	2.65	0.28	0.549
Bone	1.03	0.09	1.06	0.08	0.692
Heart	15.12	8.59	8.43	1.72	0.256
Whole brain	7.36	0.38	6.80	0.63	0.258
Gall bladder	1.97	0.49	1.57	0.56	0.405
Blood	2.37	1.16	1.64	0.18	0.344
Stomach[a]	3.68	0.03	2.20	0.22	0.0003
Small intestine[b]	2.92	1.55	1.97	0.22	0.347
Urine	146.86	63.41	106.02	69.79	0.603
Muscle	2.60	1.03	1.11	0.27	0.073
Pancreas	2.74	1.05	5.57	0.56	0.015
Skin	1.72	0.69	2.33	0.86	0.388
Adrenal gland	7.60	0.19	14.06	2.40	0.0097
Interscapular BAT	11.06	2.55	62.67	18.60	0.009
Infrascapular BAT	14.83	3.18	72.39	22.22	0.011
Axillary BAT	10.43	3.41	57.28	12.34	0.003
Anterior subcutaneous WAT	3.13	0.83	5.30	0.59	0.0211
Inguinal WAT	2.76	0.62	6.33	1.19	0.010
Visceral WAT	1.55	0.13	4.35	0.65	0.002

Ratio	Vehicle-treated group (Tissue:Blood Ratio, n=3)		CL316,243-treated group (Tissue:Blood Ratio, n=3)		*P* value[e]
Interscapular BAT/Blood	5.41	2.41	37.65	7.65	0.012
Infrascapular BAT/Blood	7.44	3.63	43.46	9.49	0.013
Axillary BAT/Blood	5.53	3.47	34.79	5.56	0.003
Anterior subcutaneous WAT/Blood	1.44	0.88	3.89	0.78	0.091
Inguinal WAT/Blood	0.77	0.38	2.66	0.34	0.023
Visceral WAT/Blood	3.73	1.66	8.54	0.64	0.003

WAT: white adipose tissue, BAT: brown adipose tissue, SD: standard deviation. [a] without stomach content, [b] with content. [c] Unpaired t-tests, two-sided, not corrected for multiple comparisons. [e] Multiple unpaired t-tests, two-sided with Welch correction.

Methods

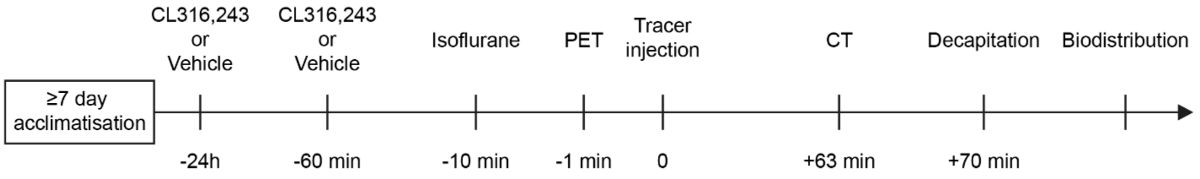

Figure S23. Study protocol. All mice (n=3 for each group) were allowed to acclimatise 1 week before the start of the study. Mice were injected with CL316,243 or vehicle (24h and 1h) prior [11C]J147 administration. Subsequently, PET acquisition followed by CT acquisition was performed. In the case of biodistribution studies, mice were decapitated after PET/CT acquisition and biodistribution was conducted 70 min post tracer injection.

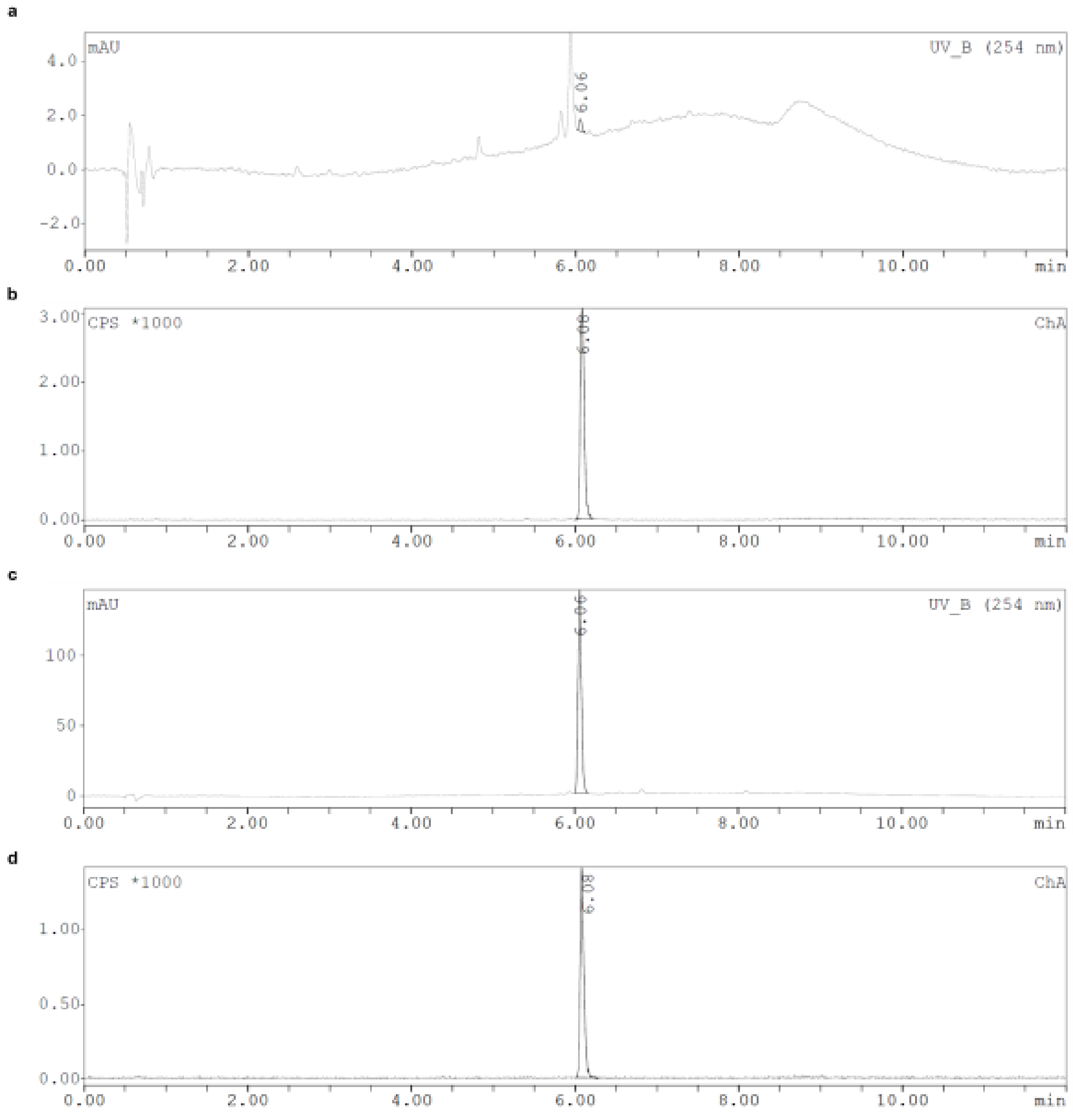

Fig. S24. HPLC chromatograms of [^{18}F]BCPP-EF: UV in mAU at 254 nm (RT: 6.06 min) (**a**) and radioactivity in counts per second (CPS) (**b**). Co-injection of BCPP-EF with [^{18}F]BCPP-EF: UV in mAU at 254 nm (**c**) and radioactivity in CPS (**d**).

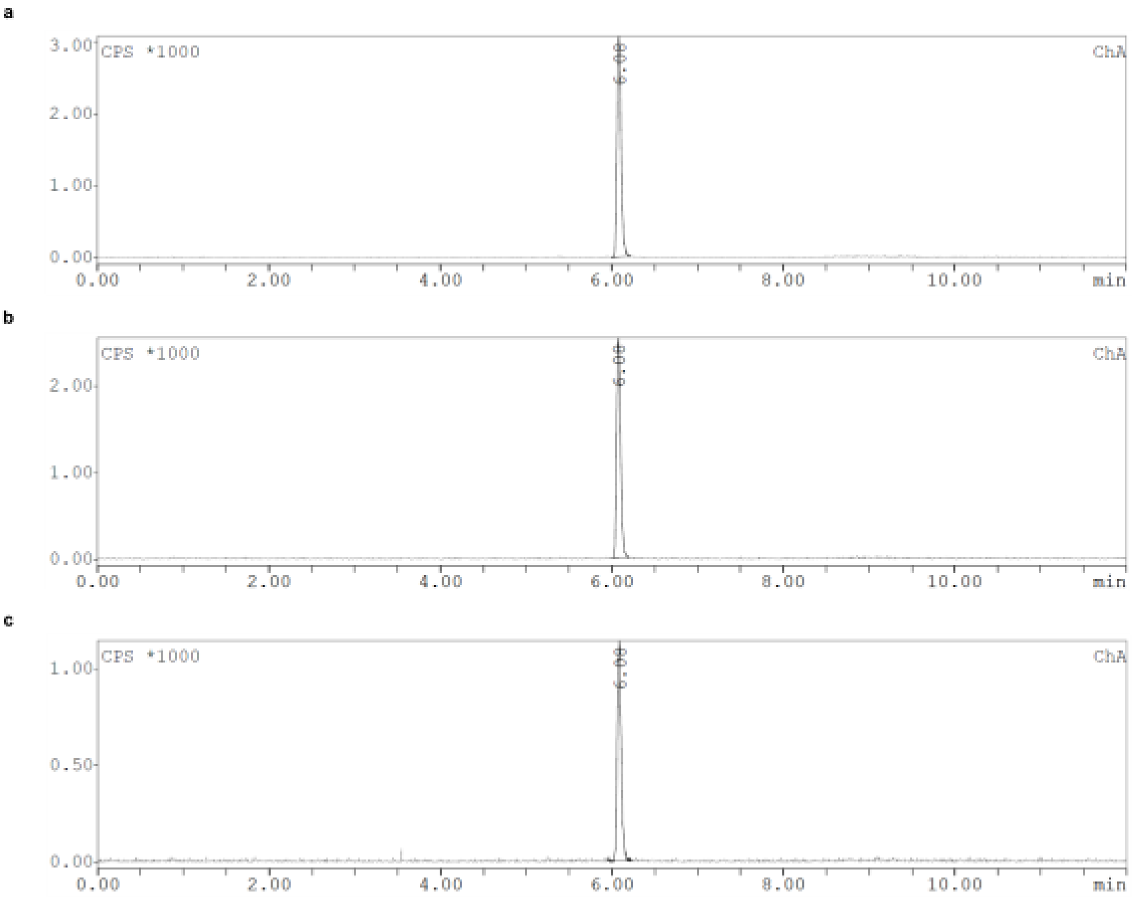

Fig. S25. Radiotracer stability of [^{18}F]BCPP-EF in formulation (0.1% Tween-80, 1% sodium ascorbate in saline) immediately after production (**a**), after 1 hour (**b**), and after 3 hours (**c**). Radioactivity is depicted in counts per seconds (CPS).

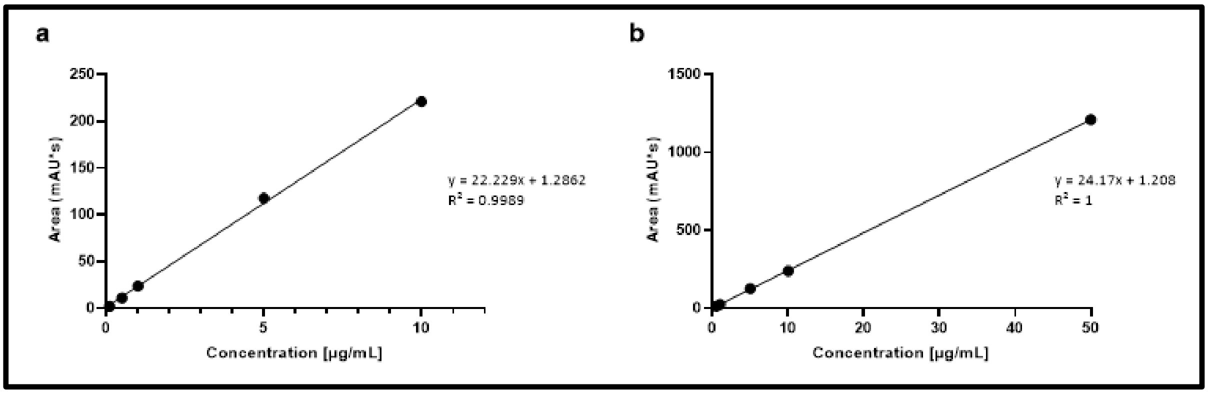

Fig. S26. Calibration curve of [^{11}C]J147 (a) and [^{18}F]BCPP-EF (b) for determining their molar activity.

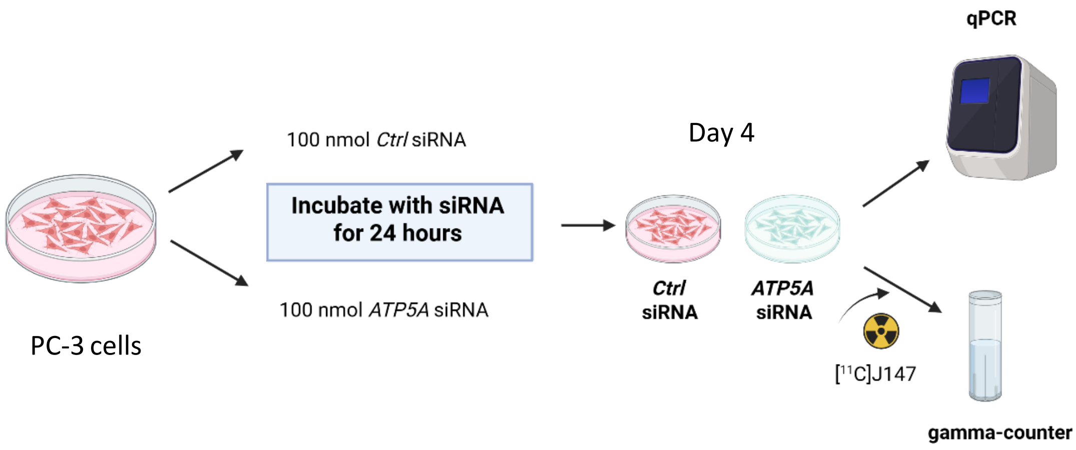

Figure S27. Experiment workflow. Cell knockdown studies using Ctrl or *ATP5A* siRNA and subsequent qPCR analysis or cell uptake studies with [^{11}C]J147. Figure created in BioRender. Schibli, R. (2026) http://BioRender.com/xsxg5cc.

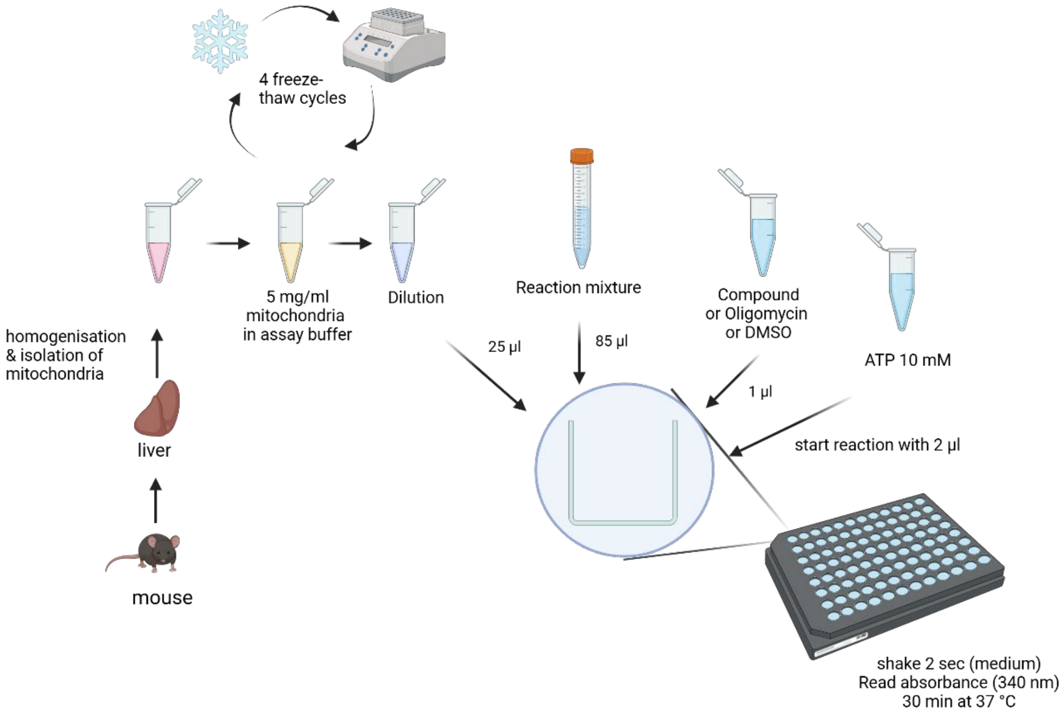

Figure S28. Schematic illustration of the ATP hydrolysis assay procedure. Mitochondria were isolated from C57BL/6N mouse liver. The mitochondrial functionality was disrupted by applying four freeze-thaw cycles in liquid nitrogen. Mitochondria, reaction mix and samples or controls were added to each well and started with the addition of ATP. The plate was immediately placed in the preheated plate reader, where it was shaken for 2 seconds and the decrease in absorbance was monitored at 340 nm for 30 minutes at 37 °C. Figure created in BioRender. Schibli, R. (2026) http://BioRender.com/ozw884r.

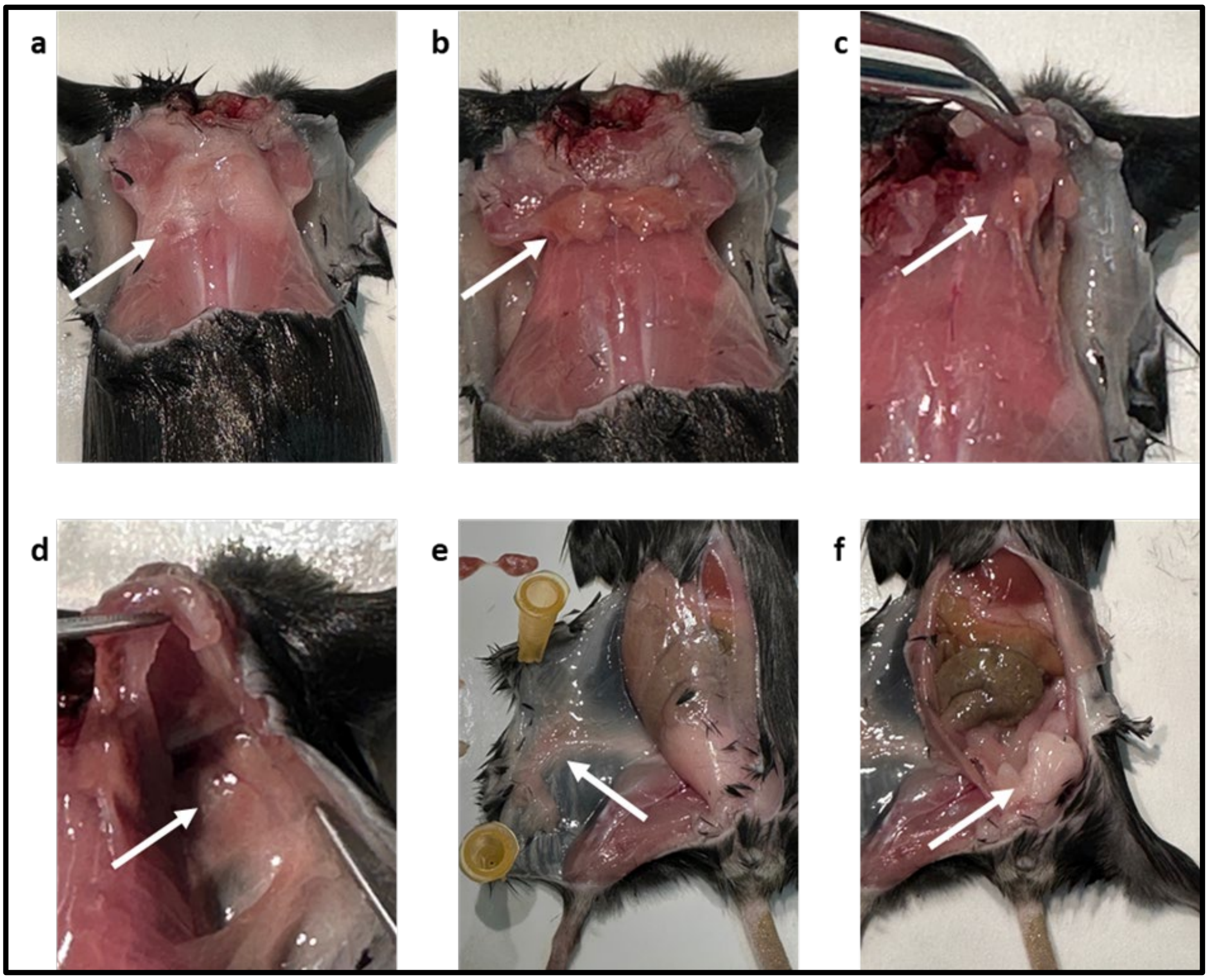

Figure S29. Dissection of adipose tissue depots in mice. To isolate adipose tissue depots, mice are first euthanized by decapitation following approval and ethical guidelines. The animal is placed in a prone position, and the skin is incised along the midline from the middle back region to the neck to expose the interscapular BAT surrounded by anterior subcutaneous WAT (**a**, white arrow). The anterior subcutaneous WAT is carefully excised, avoiding contamination with adjacent muscle or fat. The interscapular BAT (white arrow) can be visualized (**b**) as a distinct tissue located between the shoulder blades, overlaying the dorsal surface of the upper thoracic spine. The infrascapular BAT can be found under the shoulder plate, it appears as a dark-reddish-brown tissue. Shoulder plate is lifted and the infrascapular BAT, indicated with a white arrow, is sampled (**c**). The axillary BAT depots are located lateral to the upper thoracic spine and close to the junction of the forelimb and thorax. These depots appear as small, darker brown tissues compared to surrounding white and fat and muscle, and are positioned medially to the forelimb, indicated by a white arrow (**d**). The animal is then placed in a supine position, and the skin is incised along the midline from the lower abdomen to the neck. Skin is removed from internal organs and fixed to the dissection surface. Inguinal WAT (**e**, indicated by a white arrow) is located subcutaneously along the lower abdomen flanks, lateral to the hindlimbs, and is carefully separated from the skin tissue using forceps and scissors. Visceral WAT (**f**) is found within the peritoneal cavity adjacent to the testes, in males, and is dissected by gently lifting them and isolating the surrounding fat. In the picture is indicated by a white arrow.

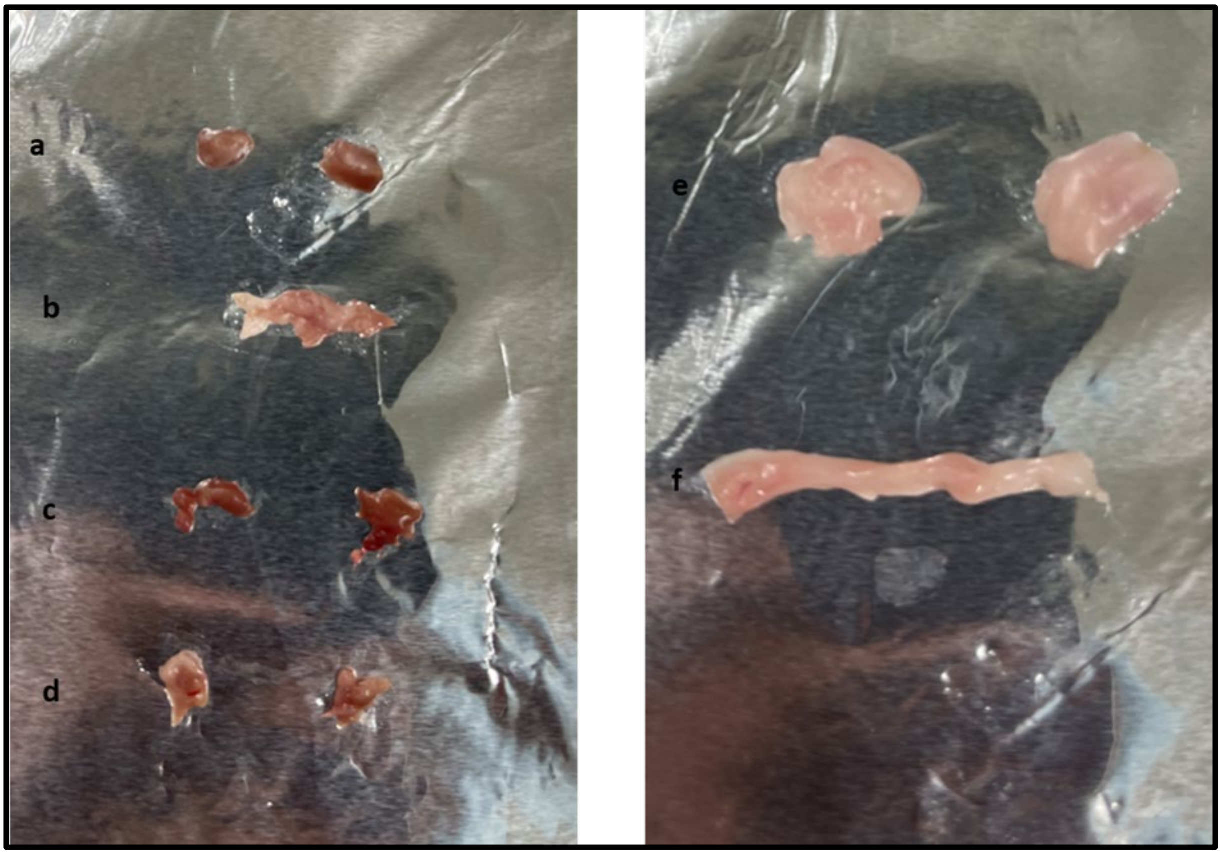

Figure S30. Dissection of adipose tissue depots in mice. The different fat depots dissected in this study are shown in this figure. Interscapular BAT, also known as the classical BAT (**a**). Anterior subcutaneous WAT (**b**). Infrascapular BAT (**c**). Axillary BAT (**d**). Visceral WAT (**e**). Inguinal WAT from one side of the body (**f**).

Synthesis

All chemicals, unless otherwise stated, were purchased from Sigma-Aldrich GmbH (Buchs, Switzerland), abcr GmbH (Karlsruhe, Germany), Fluorochem Ltd. (Hadfield, UK), Tokyo Chemical Industry Co. Ltd (Tokyo, Japan), Apollo Scientific Ltd (Cheshire, UK), Acros Organics (Geel, Belgium), Combi-Blocks, Inc. (San Diego, USA), Fisher Scientific International Inc. (Hampton, USA) and were used without further purification. Solvents for thin layer chromatography (TLC), column chromatography, and liquid-liquid extractions were purchased as commercial grade.

Reactions were monitored by TLC using silica gel 60 plates (Merck) under UV light (254 nm). Nuclear magnetic resonance (NMR) spectra (^{1}H, ^{13}C, and ^{19}F if applicable) of intermediates and final compounds were recorded on a Bruker 400 MHz spectrometer at room temperature (298 K). Chemical shifts (δ) are reported in parts per million (ppm) relative to tetramethylsilane (TMS, 0 ppm). Values of the coupling constant, *J*, are given in hertz (Hz). Multiplicities in the ^{1}H NMR spectra are described as: singlet (s), doublet (d), triplet (t), quartet (q), multiplet (m), doublet of doublet (dd), triplet of doublet (td), and broad peak (br). The chemical shifts of complex multiplets are given as the range of their occurrence. Liquid chromatography mass spectrometry (LC-MS) and purity was measured on a Waters Acquity SQ Detector 2 equipped with an HPLC using an analytical reverse phase column (BEH C18, 1.7 µm; 2.1 mm x 50 mm) with H_2O/MeCN (both containing 0.1% TFA) as mobile phase. Method: gradient from 5% to 90% MeCN in 6.5 min. High resolution mass spectrometry (HRMS) was performed by the Molecular and Biomolecular Analysis Service (MoBiAS) using a Bruker MaXis with ESI ion source

and Q-TOF analyser (ESI-Qq-TOF-MS) in positive mode and processed by the software Compass 1.5, HyStar 3.2.44, DataAnalysis 4.1 and BioTools 3.3. HRMS data are reported in m/z.

(*E*)-*N*-(2,4-dimethylphenyl)-2,2,2-trifluoro-*N'*-(3-methoxybenzylidene)acetohydrazide (J147)

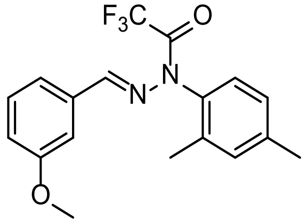

To a suspension of 2,4-dimethylphenylhydrazine hydrochloride (341 mg, 1.98 mmol, 1.5 eq) and sodium acetate (162 mg, 1.98 mmol, 1.5 eq) in water (5 mL), 3-methoxybenzaldehyde (161 µL, 1.32 mmol, 1.0 eq) in EtOH (3 mL) was added. The reaction mixture was heated to 100 ^{o}C and stirred vigorously for 30 min under N_2 atmosphere. The reaction mixture was cooled to 0 ^{o}C and an orange sticky oil accumulated at the bottom of the flask. The yellow solution was carefully decanted, and the remaining oil was washed with cold water. The remaining oil was dissolved in CH_2Cl_2 (20 mL), followed by the addition of water (25 mL), and then extracted with CH_2Cl_2 (2 x 20 mL). The combined CH_2Cl_2 layers were dried over $MgSO_4$, filtered, and concentrated *in vacuo* to afford the hydrazone intermediate. This compound was unstable when exposed to atmospheric oxygen for a prolonged time and therefore used immediately for the following step.
The hydrazone intermediate (330 mg, 1.30 mmol, 1.0 eq) and triethylamine (217 µL, 1.56 mmol, 1.2 eq) were dissolved in CH_2Cl_2 (5 mL). Trifluoroacetic anhydride (217 µL, 1.56 mmol, 1.2 eq) was added dropwise to the solution at 0 ^{o}C under N_2 atmosphere. The reaction mixture was stirred at 0 ^{o}C for 1.5 h under N_2 atmosphere. The reaction mixture was concentrated under reduced pressure and the residue was purified by column chromatography (acetone:hexane = 1:10) to afford the title compound as a yellow oil (263 mg, 0.750 mmol, 57%).
^{1}H NMR (400 MHz, $CDCl_3$): δ 7.30 (d, *J* = 7.9 Hz, 1H), 7.27 – 7.25 (m, 3H), 7.22 (d, *J* = 7.9 Hz, 1H), 7.13 (d, *J* = 7.6 Hz, 1H), 7.06 (d, *J* = 7.9 Hz, 1H), 6.96 (ddd, *J* = 8.3, 2.6, 1.0 Hz, 1H), 3.84 (s, 3H), 2.43 (s, 3H), 2.10 (s, 3H); **^{13}C NMR** (101 MHz, $CDCl_3$): δ 160.1, 144.0, 141.1, 136.4, 134.8, 132.7, 129.9, 129.7, 128.9, 128.7, 128.6, 121.1, 117.0 (q, *J* = 286.3 Hz), 117.3, 111.8, 55.4, 21.4, 17.1; **^{19}F NMR** (376 MHz, $CDCl_3$) δ -69.0; **LC-MS (ESI+):** m/z found 389.1 $[M+K]^+$, calculated for $C_{18}H_{17}F_3N_2O_2K$ 389.1; **Purity by LC-MS**: >96%; **HRMS (ESI+):** calculated for $C_{18}H_{18}F_3N_2O_2$ $[M+H]^+$ 351.1315, found 351.1319, Δ1.1 ppm.

(*E*)-*N'*-(3-(benzyloxy)benzylidene)-*N*-(2,4-dimethylphenyl)-2,2,2-trifluoroacetohydrazide (CJ9-02)

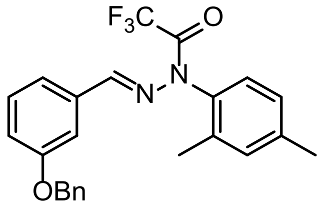

To a suspension of 2,4-dimethylphenylhydrazine hydrochloride (447 mg, 2.59 mmol, 1.5 eq) and sodium acetate (213 mg, 2.59 mmol, 1.5 eq) in water (5 mL) was added 3-benzyloxybenzaldehyde (366 mg, 1.73 mmol, 1.0 eq) in EtOH (3 mL). The reaction mixture was heated to 75 ^{o}C and stirred vigorously for 20 min. The reaction mixture was cooled to 0 ^{o}C and an orange sticky oil accumulated at the bottom of the flask. The yellow solution was carefully decanted, and the remaining oil was washed with cold water, followed by hexane to afford the hydrazone intermediate. This compound was unstable when exposed to atmospheric oxygen for a prolonged time and therefore used immediately for the following step.

The hydrazone intermediate (562 mg, 1.70 mmol, 1.0 eq) and trimethylamine (285 µL, 2.04 mmol, 1.2 eq) were dissolved in CH_2Cl_2 (8 mL). Trifluoroacetic anhydride (284 µL, 2.04 mmol, 1.2 eq) was added dropwise to the solution at 0 ^oC under N_2 atmosphere. The reaction mixture was stirred at 0 ^oC for 2 h under N_2 atmosphere. The reaction mixture was concentrated under reduced pressure and the residue was purified by column chromatography (acetone:hexane = 1:10) to afford the title compound as a light-yellow solid (651 mg, 1.53 mmol, 88%).

^{1}H NMR (400 MHz, $CDCl_3$): δ 7.49 – 7.26 (m, 9H), 7.23 (dd, *J* = 8.2, 1.9 Hz, 1H), 7.15 (d, *J* = 7.5 Hz, 1H), 7.09 – 7.03 (m, 2H), 5.12 (s, 2H), 2.45 (s, 3H), 2.12 (s, 3H); **^{13}C NMR** (101 MHz, $CDCl_3$) δ 159.2, 157.4 (d, *J* = 36.4 Hz), 144.0, 141.1, 136.7, 136.3, 134.8, 132.7, 129.9, 129.7, 128.9, 128.7, 128.6, 128.2, 127.6, 121.5, 117.0 (q, *J* = 287.0 Hz), 118.2, 112.7, 70.1, 21.4, 17.1; **^{19}F NMR** (376 MHz, $CDCl_3$) δ -68.8; **LC-MS (ESI+):** m/z found 426.0 [M], calculated for $C_{24}H_{21}F_3N_2O_2$ 426.4; **Purity by LC-MS**: >95%; **HRMS (ESI+):** calculated for $C_{24}H_{21}F_3N_2O_2Na$ $[M+Na]^+$ 449.1447, found 449.1448, Δ -0.1 ppm.

(*E*)-*N*-(2,4-dimethylphenyl)-2,2,2-trifluoro-*N'*-(3-hydroxybenzylidene)acetohydrazide (J147 precursor)

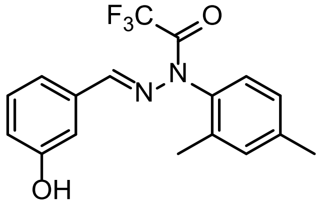

To a stirred solution of CJ9-02 (181 mg, 0.424 mmol, 1.0 eq) in Me_2S (3 mL) and CH_2Cl_2 (3 mL), $BF_3{\cdot}Et_2O$ (266 µL, 2.12 mmol, 5.0 eq) was added dropwise at room temperature. The reaction mixture was stirred at room temperature for 8 h. The reaction mixture was poured into ice water and extracted with EtOAc (2 × 25 mL). The combined organic layers were washed with brine, dried over Na_2SO_4, filtered, and concentrated *in vacuo*. The residue was purified by column chromatography (acetone/hexane = 1:7 to 1:5) to afford the title compound as a light-yellow solid (117 mg, 0.348 mmol, 82%).

^{1}H NMR (400 MHz, $CDCl_3$) δ 7.26 – 7.16 (m, 5H), 7.09 (dt, *J* = 7.6, 1.3 Hz, 1H), 7.05 (d, *J* = 7.9 Hz, 1H), 6.86 (ddd, *J* = 8.1, 2.6, 1.1 Hz, 1H), 5.30 (s, 1H), 2.41 (s, 3H), 2.08 (s, 3H); **^{13}C NMR** (101 MHz, $CDCl_3$) δ 156.1, 144.0, 141.1, 136.2, 134.8, 132.6, 130.0, 129.5, 128.8, 128.5, 121.2, 118.1, 117.3 (q, *J* = 288.8 Hz), 113.5, 21.3, 17.0; **^{19}F NMR** (376 MHz, $CDCl_3$) δ -68.9; **LC-MS (ESI+):** m/z found 336.0 [M], calculated for $C_{17}H_{15}F_3N_2O_2$ 336.3; **Purity by LC-MS**: >99%; **HRMS (ESI+):** calculated for $C_{17}H_{15}F_3N_2O_2Na$ $[M+Na]^+$ 359.0978, found 359.0978, Δ1.1 ppm.

Radiosynthesis

[^{11}C]J147. [^{11}C]CO_2 was produced via the ^{14}N(p,a)^{11}C nuclear reaction by proton bombardment of nitrogen gas fortified with 0.5% oxygen in a Cyclone 18/9 cyclotron (17-MeV; IBA, Ottignies-Louvain-la-Neuve, Belgium). [^{11}C]CO_2 was reduced to [^{11}C]CH_4 via a nickel-based catalytic reduction which consequently was converted to [^{11}C]CH_3I by gas phase iodination. Further conversion of [^{11}C]CH_3I into [^{11}C]CH_3OTf was achieved by passing it through a pre-heated column (180 °C) containing silver triflate on graphitised carbon spheres.

The radiosynthesis of [^{11}C]J147 was accomplished in a one-step procedure via *O*-methylation of the J147 hydroxyl precursor using [^{11}C]CH_3OTf. The [^{11}C]CH_3OTf was bubbled into a solution of precursor (0.5-1.0 mg) in anhydrous CH_3CN (0.5 mL) and NaOH (5 M, 2.5 µL). The mixture was left at room temperature (RT) for 60 seconds, followed by dilution with water containing 0.1% H_3PO_4 (1.6 mL).

Subsequently, this was injected into a semi-preparative high-performance liquid chromatography (Hitachi LaChrom Elite, Tokyo, Japan) equipped with a reverse phase column (Waters, Symmetry C18 Prep Column, 100 A, 5 µm, 7.8 mm × 50 mm) using a gradient system of acetonitrile (solvent A) and 0.1% H_3PO_4 in milliQ water (solvent B): 0-8 min, 35% A; 8-12 min, 35-60% A; 12-18 min, 60-75% A; 18-21 min, 75-95% A; 21-25 min, 95-35% A; flow rate, 4 mL/min; wavelength 254 nm. The radiotracer was collected into water (10 mL) and passed through a Sep-Pak C18 Plus Light cartridge (Waters, Massachusetts, USA), which was washed with water (6 mL) and eluted with EtOH (0.5 mL) into a sterile vial. The radiotracer was formulated with sterile-filtered PEG300 (2.5 mL) and sterile PBS, pH 7.4 (7 mL), resulting in a total volume of 10 mL.

Quality control was performed by injecting 20 µL of formulated tracer solution into a high-performance liquid chromatography (HPLC) using an analytical Agilent 1100 series HPLC system (Agilent Technologies, California, USA) equipped with UV multi-wavelength detector, GabiStar radiodetector (Elysia Raytest, Liège, Belgium), and a reverse phase column (ACE 3 C18-AR 50 × 4.6 mm, 3 µm) with a gradient system of CH_3CN (solvent A) and 0.1% TFA in milliQ water (solvent B): 0-5 min, 30% A; 5-7 min, 30-95% A; 7-10 min, 95-30% A; 10-12 min, 30% A; flow rate, 1 mL/min; wavelength 254 nm. The identity of [^{11}C]J147 was confirmed by co-injection of non-radiolabelled J147 (Supplementary Fig. S1).

[^{18}F]BCPP-EF. [^{18}F]Fluoride produced by $^{18}F^0(p,n)^{18}F$ nuclear reaction was transferred from a Cyclone 18/9 cyclotron (18-MeV; IBA, Belgium) to a shielded hotcell, and trapped on a pre-conditioned anion-exchange cartridge (Waters SepPak Accell QMA cartridge carbonate). A solution of Kryptofix® 2.2.2 (10 mg), K_2CO_3 (2.5 mg) in H_2O:CH_3CN (3:7, 1 mL) was used to elute the radioactivity to the reaction vial. Subsequently, the solvent was removed by heating at 110 °C under reduced pressure and gentle nitrogen flow. Anhydrous CH_3CN (3 x 1 mL) was used for azeotropic drying before adding the tosylate precursor (2 mg of precursor in 1 mL CH_3CN). The reaction mixture was stirred for 10 min at 80 °C and subsequently diluted with H_2O:CH_3CN (7:3, 2.5 mL) before the crude product was injected onto a semipreparative HPLC column (µBondapak C18 7.8 x 300 mm Waters) and eluted with CH_3CN/0.1% H_3PO_4, mH_2O (gradient starting from 10% CH_3CN to 90%). The radioactive peak at around 12 min was collected, diluted with 5 mL H_2O and passed through a C18 light cartridge (pre-conditioned with 5 mL of EtOH and 10 mL of H_2O). After washing the cartridge with 5 mL H_2O, [^{18}F]BCPP-EF was eluted with 0.5 mL EtOH and formulated using 0.1% Tween-80, 1% sodium ascorbate in saline. The molar activities of [^{18}F]BCPP-EF were 103.27 ± 68.29 GBq/µmol (n=8) at the end of synthesis.

Quality control was performed by injecting 20 µL of formulated tracer solution into a high-performance liquid chromatography (HPLC) using an analytical Agilent 1100 series HPLC system (Agilent Technologies, California, USA) equipped with UV multi-wavelength detector, GabiStar radiodetector (Elysia Raytest, Liège, Belgium), and a reverse phase column (ACE 3 C18-AR 50 × 4.6 mm) with a gradient system of CH_3CN (Solvent A) and 0.1% TFA in milliQ water (Solvent B): 0 min, 10% A; 0-5 min 50% A; 5-7 min 80% A; 7-10 min 10% A; flow rate, 1 mL/min; wavelength 254 nm. The radiotracer identity and stability in the formulation over a period of 3 hours were confirmed in the Supplementary Fig S5 and S6, respectively.

Molar activities were calculated based on UV intensity of the formulated tracer product and calibration curves of the corresponding non-radioactive standards. The product was confirmed by comparing the retention time of the injected radiotracer with the standard reference (BCPP-EF). (Supplementary Fig. S23) Stability of [^{18}F]BCPP-EF is depicted in Supplementary Fig. S24. The molar activity was calculated from the linear regression of a UV-intensity-based calibration curve of the non-radioactive reference. (Supplementary Fig. S25)

Haematoxylin and Eosin staining

Slides were rinsed with tap water for 1.5 min, submerged in Haematoxylin solution modified acc. to Gill III (Merck) for 4 min, rinsed twice in tap water and subsequently with 0.1% HCl solution (2 × 2 sec). Slides were washed under running tap water for 2 min, submerged in Eosin Y-solution 0.5% aqueous (Merck) for 10 min, and submerged once in tap water. Slides were submerged in 100% ethanol (2 × 30 sec) followed by xylene (1 × 2 min) and finally mounted with Eukitt© (Sigma-Aldrich, Missouri, USA).

Image acquisition for H&E staining was performed with a Pannoramic 250 digital slide scanner (3DHistech, Budapest, Hungary) and converted to 40x-magnified images using CaseViewer software (Version 2.4, 3DHistech).

Immunohistochemical staining

Sections were fixed in acetone (−20 °C) for 2 min and rehydrated in methanol (4 °C) for 5 min, followed by 1h blocking (10% donkey serum) at RT. Sections were incubated with primary antibody anti-UCP-1 polyclonal antibody, (PA-124894, Invitrogen, 1:200 dilution) in blocking solution for 12h at 4 °C. After washing with 3X PBST, secondary antibody (donkey anti-rabbit Alexa Fluor 568, A10042, Invitrogen, 1:1000 for UCP-1) was added for 1h at RT. After washing with 3X PBST, Sections were incubated with primary antibody anti-ATP5A1 monoclonal antibody (MA5-32609, XE358225A, Thermo Fisher, 1:200 dilution) in blocking solution for 12h at 4 °C. After washing with 3X PBST, secondary antibody (donkey anti-rabbit Alexa Fluor 647, 711-605-152, jacksonimmuno, 1:500 for ATP5A) and DAPI (422801, BioLegend, 1:1000) was added 1 hour min at RT mounting with ProLong™ Diamond Antifade Mountant (P36961 invitrogen).

Image acquisition for immunofluorescence staining was performed with a Pannoramic 250 digital slide scanner (3DHistech, Budapest, Hungary) and converted to 40x-magnified images using CaseViewer software (Version 2.4, 3DHistech). The area fraction of BAT and WAT was evaluated using immunofluorescence signal analysis of the entire tissue slice image. The area fraction compares the percentage of the stained area representing ATP5A or UCP1 expression to the total area being analysed. Fiji is Just ImageJ (version 2.3.0/1.53q) was used for visualisation and quantification. Results are displayed as an area fraction of expression on pictures obtained with the slide scanner.

Isolation of functional mouse liver mitochondria

Liver mitochondria were isolated from 5-month-old male C57BL/6NCrl mice (Charles River, n=4). Sex was not considered a critical variable, as mitochondrial yield and integrity are comparable between male and female mice. The liver was rapidly dissected using a pre-cooled scalpel (4 °C), washed in ice-cold isolation buffer (100 mL:5 mL 0.2 M Tris-MOPS, 1 mL 0.1 M EGTA/Tris, 20 mL 1 M sucrose, 74 mL distilled water), and minced on ice. Tissue was homogenized in pre-cooled Potter homogenizer with 10 ml isolation buffer (5 strokes at 1200 rpm). The homogenate was centrifuged at 600 × g for 10 min at 4 °C; the supernatant was then centrifuged at 7000 × g for 10 min. Final pellets were carefully resuspended in ~200 µL isolation buffer and centrifuged once more at 7000 × g for 10 min. The mitochondrial pellet was resuspended. Protein concentration was determined using a BCA assay (Pierce™, Cat No. 23225), and mitochondria were stored in aliquots at −80 °C.

ATP hydrolysis assay in isolated mitochondria

Assay buffer (25 mM HEPES (pH 7.8), 25 mM KCl, 2 mM $MgCl_2 \cdot 6H_2O$), 1 M Tris-HCl buffer (pH 8.1), 200 mM KCl and 1M $MgCl_2$ anhydrous were prepared in nanopure water, adjusted to the required Ph using KOH, and stored in aliquots at -20 °C. On the day of the experiment, assay and Tris-HCl buffers were

thawed at room temperature. Working solutions were freshly prepared: 10mM ATP and 40 mM PEP in Tris buffer and 12 mM NADH in nanopure water and kept on ice. Isolated liver mitochondria were diluted to 5 mg/ml in assay buffer, frozen in liquid nitrogen for 3 min, and thawed at 37 °C in a thermomixer; this freeze-thaw cycle was repeated four times. The suspension was then diluted to 1.32 mg/mL and kept on ice. The reaction mix was prepared on ice and contained: 2.84 mM KCl, 1.42 mM $MgCl_2$, 1.42 U/mL pyruvate kinase, 1.95 U/mL lactate dehydrogenase, 0.85 µM FCCP, 0.26 µM antimycin A, 1.13 mM PEP and 638.12 µM NADH. Compounds were diluted in PBS. In a 96-well clear tissue culture plate (TPP®, Cat No.: 92096), each well received 85 µl of reaction mix, 25 µl of mitochondrial suspension, and 1 µl of diluted compound sample (in triplicate). The reaction was initiated by adding 2 µl ATP, followed by a 2-second shake, and absorbance at 340 nm was measured at 37 °C for 30 min using a preheated plate reader (Agilent, BioTek Synergy HT).

Blood glucose measurements.

Blood sugar was measured with a Contour XT Blood Glucose Meter (Contour 84188795) and Contour Next Sensor Strips (Contour 84191451).
