# Supplementary material for: ATP synthase is a promising target for identifying activated and non-activated adipose tissues
Source: Nat Commun. 2026 Apr 15;17:5233. doi: 10.1038/s41467-026-71343-w (PMC13260929; doi:10.1038/s41467-026-71343-w)
Supplement: Supplementary file 2 — Description Of Additional Supplementary File [file 41467_2026_71343_MOESM2_ESM.pdf]

Description of Additional supplementary files

Supplementary Movie 1. Representative total averaged PET/CT imaging (time frame 0-60 min) with [11C]J147 in vehicle-treated Balb/c mice. Normalised to standardised uptake value (SUV) 3.5.

Supplementary Movie 2. Representative total averaged PET/CT imaging (time frame 0-60 min) with [11C]J147 in CL316.243-treated Balb/c mice. Normalised to standardised uptake value (SUV) 3.5.
