# Supplementary figures and images for: ATP synthase is a promising target for identifying activated and non-activated adipose tissues
Source: Nat Commun. 2026 Apr 15;17:5233. doi: 10.1038/s41467-026-71343-w (PMC13260929; doi:10.1038/s41467-026-71343-w)

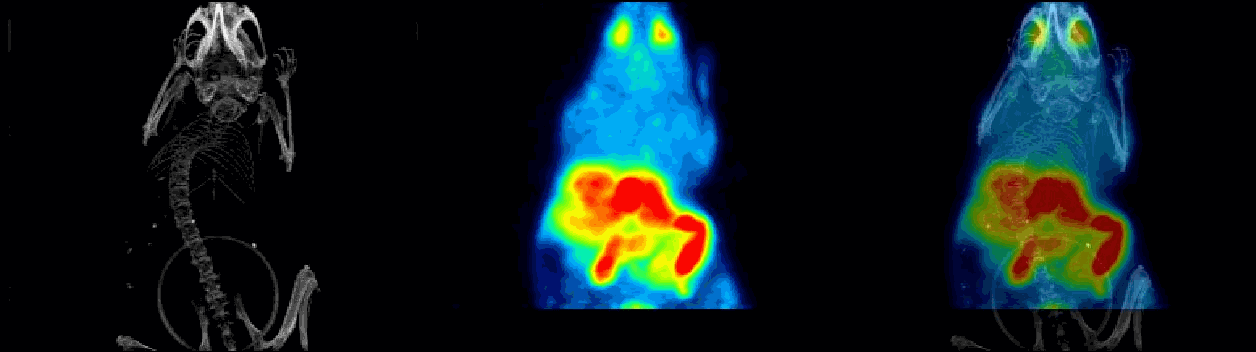

Supplement: Supplementary file 3 — Supplementary Movie 1 [file 41467_2026_71343_MOESM3_ESM.gif]

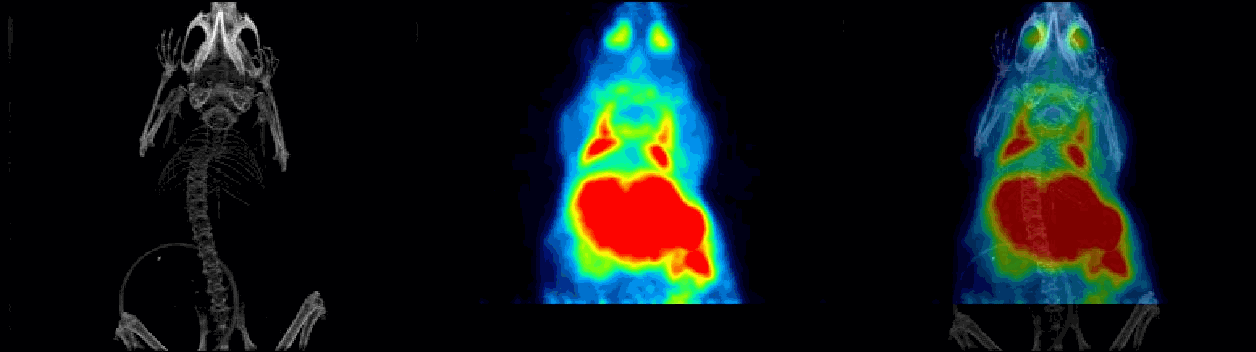

Supplement: Supplementary file 4 — Supplementary Movie 2 [file 41467_2026_71343_MOESM4_ESM.gif]
