# Supplementary material for: ATP synthase is a promising target for identifying activated and non-activated adipose tissues
Source: Nat Commun. 2026 Apr 15;17:5233. doi: 10.1038/s41467-026-71343-w (PMC13260929; doi:10.1038/s41467-026-71343-w)
Supplement: Supplementary file 5 — Reporting Summary [file 41467_2026_71343_MOESM5_ESM.pdf]

nature portfolio

Corresponding author(s): Caitlin V.M.L. Jie, Aro Delparente, Tongtong Wang, Lisa Reichert, Petra Krajnovic, Marc Schläppi, Lukas Reiniger, Laura T. L. Brandt, Claudia Keller, Julien Orts, Roland Riek, Stefanie D. Krämer, Markus Stoffel, Christian Wolfrum, Roger Schibli, Linjing Mu

Last updated by author(s): 20/02/26

Reporting Summary

Nature Portfolio wishes to improve the reproducibility of the work that we publish. This form provides structure for consistency and transparency in reporting. For further information on Nature Portfolio policies, see our Editorial Policies and the Editorial Policy Checklist.

Statistics

For all statistical analyses, confirm that the following items are present in the figure legend, table legend, main text, or Methods section.

n/a	Confirmed	
☐	☒	The exact sample size (*n*) for each experimental group/condition, given as a discrete number and unit of measurement
☐	☒	A statement on whether measurements were taken from distinct samples or whether the same sample was measured repeatedly
☐	☒	The statistical test(s) used AND whether they are one- or two-sided<br>*Only common tests should be described solely by name; describe more complex techniques in the Methods section.*
☒	☐	A description of all covariates tested
☒	☐	A description of any assumptions or corrections, such as tests of normality and adjustment for multiple comparisons
☐	☒	A full description of the statistical parameters including central tendency (e.g. means) or other basic estimates (e.g. regression coefficient) AND variation (e.g. standard deviation) or associated estimates of uncertainty (e.g. confidence intervals)
☐	☒	For null hypothesis testing, the test statistic (e.g. *F*, *t*, *r*) with confidence intervals, effect sizes, degrees of freedom and *P* value noted<br>*Give P values as exact values whenever suitable.*
☒	☐	For Bayesian analysis, information on the choice of priors and Markov chain Monte Carlo settings
☒	☐	For hierarchical and complex designs, identification of the appropriate level for tests and full reporting of outcomes
☒	☐	Estimates of effect sizes (e.g. Cohen's *d*, Pearson's *r*), indicating how they were calculated

Our web collection on statistics for biologists contains articles on many of the points above.

Software and code

Policy information about availability of computer code

Data collection	Bruker MaXis with ESI ion source and Q-TOF analyser (ESI-Qq-TOF-MS); Agilent 1100 series HPLC system; CaseViewer software (Version 2.4, 3DHistech); Fiji is Just ImageJ (version 2.3.0/1.53q); Agilent, BioTek Synergy H, Genesys 10 UV spectrometer (Thermospectronics, USA); BCA assay (Pierce™, Cat No. 23225); Image Quant system (GE Healthcare Life Sciences); Super Argus PET/CT scanner (Sedecal S.A., Madrid, Spain); γ-counter (Wizard, PerkinElmer).
Data analysis	All our data were analyzed using published softwares including Graphpad Prism and PMOD software.

For manuscripts utilizing custom algorithms or software that are central to the research but not yet described in published literature, software must be made available to editors and reviewers. We strongly encourage code deposition in a community repository (e.g. GitHub). See the Nature Portfolio guidelines for submitting code & software for further information.

Data

Policy information about availability of data

All manuscripts must include a data availability statement. This statement should provide the following information, where applicable:

- Accession codes, unique identifiers, or web links for publicly available datasets
- A description of any restrictions on data availability
- For clinical datasets or third party data, please ensure that the statement adheres to our policy

All data supporting the results of this study can be found in the article, Supplementary Information, and Source Data files. Source data are provided with this paper.

Research involving human participants, their data, or biological material

Policy information about studies with human participants or human data. See also policy information about sex, gender (identity/presentation), and sexual orientation and race, ethnicity and racism.

Reporting on sex and gender	No human participates were included.
Reporting on race, ethnicity, or other socially relevant groupings	No human participates were included.
Population characteristics	No human participates were included.
Recruitment	No human participates were included.
Ethics oversight	No human participates were included.

Note that full information on the approval of the study protocol must also be provided in the manuscript.

Field-specific reporting

Please select the one below that is the best fit for your research. If you are not sure, read the appropriate sections before making your selection.

[x] Life sciences [ ] Behavioural & social sciences [ ] Ecological, evolutionary & environmental sciences

For a reference copy of the document with all sections, see nature.com/documents/nr-reporting-summary-flat.pdf

Life sciences study design

All studies must disclose on these points even when the disclosure is negative.

Sample size	Based on the animal license guidelines (3Rs) and our experience with PET, we typically use 3-4 animals per study. For this study, we used n = 3 for each of the two sexes, giving a total group size of n = 6. For the microscopy studies n = 2 was used because of the availability of STAM mice, and to have enough tissues to perform the Western Blot experiments. In vitro experiments were performed on n ≥ 3 samples.
Data exclusions	Data were not excluded.
Replication	PET imaging was performed on different days with reproducible results. For example, experiments with females and males were performed at different times of the day and month. In vitro and autoradiography experiments were performed on different days with different biological replicates.
Randomization	Allocation was random.
Blinding	The investigators were blinded to group allocation during data collection, organ sampling and data analysis.

Reporting for specific materials, systems and methods

We require information from authors about some types of materials, experimental systems and methods used in many studies. Here, indicate whether each material, system or method listed is relevant to your study. If you are not sure if a list item applies to your research, read the appropriate section before selecting a response.

Materials & experimental systems

n/a	Involved in the study
☐	☒ Antibodies
☐	☒ Eukaryotic cell lines
☒	☐ Palaeontology and archaeology
☐	☒ Animals and other organisms
☒	☐ Clinical data
☒	☐ Dual use research of concern
☒	☐ Plants

Methods

n/a	Involved in the study
☒	☐ ChIP-seq
☒	☐ Flow cytometry
☒	☐ MRI-based neuroimaging

Antibodies

Antibodies used	anti-ATP5A1 monoclonal antibody (MA5-32609, Thermo Fisher); anti-UCP-1 polyclonal antibody (PA-124894, Invitrogen); anti-β-actin (ab8227, abcam); donkey anti-rabbit Alexa Fluor 568 (A10042, Invitrogen); donkey anti-rabbit Alexa Fluor 647 (711-605-152, jacksonimmuno)
Validation	anti-ATP5A1 monoclonal antibody (MA5-32609, Thermo Fisher) is reactive on Human, mouse and rat as described in the Manifacturer's product details. anti-UCP-1 polyclonal antibody, (PA-124894, Invitrogen) has been used in C. elegans, human, mouse, non-human primates, rat and rhesus moneky tissues in different publications as stated on the manifacturer's product details. anti-β-actin (ab8227, abcam) the manifacturer provides a list of the reactivity data on https://www.abcam.com/en-us/products/primary-antibodies/beta-actin-antibody-loading-control-ab8227

Eukaryotic cell lines

Policy information about cell lines and Sex and Gender in Research

Cell line source(s)	PC3 cells, male human patient
Authentication	Authentication not available. This cell line has been often used in our lab for different studies over many years and results have been confirmed its identity.
Mycoplasma contamination	Cell-line is routinely tested in-house for mycoplasma contaminations and results were negative.
Commonly misidentified lines (See ICLAC register)	No commonly misidentified cell lines were used in this study.

Animals and other research organisms

Policy information about studies involving animals; ARRIVE guidelines recommended for reporting animal research, and Sex and Gender in Research

Laboratory animals	Both female and male BALB/c mice were supplied by Charles River (Sulzberg, Germany). The female C57BL/6;C3H mice were supplied by the Jackson Laboratory in New York, USA. Male C57BL6/NRj for the STAM mice model were supplied by the Janvier Labs in France. All animals were housed under specific pathogen-free conditions (23 °C, 48% humidity, a 12-hour light/dark cycle and ad libitum access to food and water). The animals were allowed to acclimatise for one week prior to the experiments. All animal experiments were conducted in accordance with the ARRIVE guidelines and Swiss animal welfare legislation. They were approved by the Cantonal Veterinary Office of Zurich, Switzerland, and were carried out at ETH Zurich.
Wild animals	This study did not involve wild animals.
Reporting on sex	Both female and male mice were used in this study.
Field-collected samples	No field-collection was performed.
Ethics oversight	Experiments were approved by the cantonal Veterinary Office of Zurich, Switzerland, under the license 34491 and 36342 (National Number).

Note that full information on the approval of the study protocol must also be provided in the manuscript.

Plants

Seed stocks	No plants were included.
Novel plant genotypes	No plants were included.
Authentication	No plants were included.
