# Supplementary material for: ATP synthase is a promising target for identifying activated and non-activated adipose tissues
Source: Nat Commun. 2026 Apr 15;17:5233. doi: 10.1038/s41467-026-71343-w (PMC13260929; doi:10.1038/s41467-026-71343-w)
Supplement: Supplementary file 6 — Transparent Peer Review file [file 41467_2026_71343_MOESM6_ESM.pdf]

nature portfolio

Peer Review File

ATP Synthase: a Promising Target for Identifying Activated and Non-activated Adipose Tissues

Corresponding Author: Dr Linjing Mu

This file contains all reviewer reports in order by version, followed by all author rebuttals in order by version.

Version 0:

Reviewer comments:

Reviewer #1

(Remarks to the Author)
This paper by Jie et al. describes the evaluation of ATP synthase for imaging activated and non-activated adipose tissues using the radioligand [11C]J147. Although [11C]J147 is not a novel tracer, as its synthesis and potential application in Alzheimer's disease imaging have been previously reported (Bioorg. Med. Chem. Lett. 2013, 23(2):524-527. doi: 10.1016/j.bmcl.2012.11.031.), the authors are the first to apply it to BAT imaging. Such an approach could be valuable for PET-based studies on adipose tissue metabolism and drug development for metabolic disorders. However, the in vitro autoradiography studies and the in vivo PET evaluation of [11C]J147 remain incomplete. Specifically, further validation in pathological models of metabolic diseases and direct comparison with additional established BAT imaging tracers would strengthen the conclusions.
General Comments:
1. It would be helpful for the authors to provide more information on the selectivity and specific binding of [11C]J147. The in vitro autoradiography experiment only tested the self-blocking effect. What about the blocking effects of other selective ATP synthase inhibitors? Different blockers should also be used in the in vivo PET imaging to verify specific binding.
2. The authors should use literature or experimental data to explain the expression levels of BAT and WAT in mice of different ages, and provide an explanation for the selection of 14-15 week-old and 12-13 month-old mice for the PET imaging study.
3. What is the imaging effect of [18F]BCPP-EF and [11C]J147 in younger mice? Focusing on 12-13 month-old mice may limit the broader understanding of these tracers, and including data from younger mice could provide a more comprehensive comparison.
4. How is the position and volume of interscapular BAT in mice? It might be useful for the authors to explore ex vivo autoradiography and H&E staining to demonstrate that [11C]J147 uptake occurs throughout the entire BAT, rather than being limited to a localized region.
5. In line 256, authors should add statement on the attributes and limitations of these PET-ligands targeting glucose metabolism, translocate protein and mitochondrial membrane.
6. It would be helpful for the authors to provide more detailed information in the introduction about the relationship between ATP synthase and BAT, including how they are regulated, the potential metabolic pathways they are involved in, and their possible roles in metabolic diseases.
7. For [11C]J147, its application in animal models of metabolic diseases, such as obesity and diabetes, should be evaluated to assess its imaging effects under pathological conditions. Additionally, further investigation is required to explore its imaging performance and pharmacokinetic properties in non-human primates (NHPs).

Specific Comments:
1. In line 85 "The molar activities of [11C]J147 were 257 ± 154 GBq/µmol (n =19) at the end of synthesis." Please provide calibration curves for determining molar activity and provide the molar activities of [18F]BCPP-EF in manuscript.
2. In line 90, lack of blocking conditions (J147: 100 µM). The author uses such a high concentration because lower concentrations do not have a blocking effect?
3. In line 101, why was siRNA used to transfect PC-3 cells? Could the author provide a reason for not using a standard tool cell line?
4. In figure 1, scale for autoradiography can be listed with real-time numeric (instead of low and high).
5. In line 137, why are Balb/c mice used for immunofluorescence staining and C57BL6 mice used for PET imaging? Please explain the reason.

6. In figure 2b-d and 2g-2l, the author should provide PET-CT or PET-MRI fusion images to better help identify the locations of organs and BAT.
7. In section “[11C]J147 detects interscapular BAT in healthy mice in vivo”, the author could provide dynamic time-activity curves (TAC) of different brain regions in mice, given that [11C]J147 is used as an AD imaging tracer. Investigating the uptake and distribution in the brain is equally important, and this information could be included in the supplementary materials.
8. In figure 4, the author performed a 0-60 minute PET scan, and it can be seen that [11C]J147 has a relatively slow wash-out. Were PET scans done at 90 minutes or 120 minutes?
9. In line 313 “Female C57BL/6;C3H mice (n=3, 14-15 weeks old, 19-25 g and n=4, 51.0-52.9 weeks old, 22-28 g)were supplied by Jackson laboratory and female Balb/c (n=6, 14-15 weeks old, 19-22 g) were supplied by Charles River (Sulzberg, Germany)”. The author should clarify the reason for choosing female mice for the experimental design in the manuscript. Were male mice considered? and if so, what factors led to the selection of female mice? Additionally, is the BAT expression level consistent between male and female mice?
10. In line 384, is the radiotracer and blocker co-incubated for 30 minutes, or are they incubated sequentially for 30 minutes each?
11. In line 386, the dose of radiotracer should be clearly specified in autoradiography studies.

Reviewer #2

(Remarks to the Author)
I co-reviewed this manuscript with one of the reviewers who provided the listed reports. This is part of the Nature Communications initiative to facilitate training in peer review and to provide appropriate recognition for Early Career Researchers who co-review manuscripts.

Reviewer #3

(Remarks to the Author)
In this work the authors explore ATP synthase (ATP5A) as a potential imaging target for identifying activated and non-activated adipose tissue. They synthesized [11C]J147, a curcumin derivative, as the novel radiotracer for Positron Emission Tomography (PET) imaging of brown adipose tissue (BAT) and white adipose tissue (WAT). The radiotracer was tested under vehicle or CL316, 243 stimulation of the fat depots. In vivo the radiotracer was also evaluated in mice of young and old age. Uptake of the radiotracer was compared to [18F]FDG, current clinical standard for identification of activated fat, and [18F]BCPP-EF, a mitochondrial complex 1 marker. Efforts to validate imaging findings were immunohistochemistry for ATP5A and UCP1, autoradiography, siRNA knockdown of ATP5A, and biodistribution studies. Given that adipose tissue is a therapeutic target to combat obesity and other metabolic diseases, imaging biomarkers for drug screening are an important need.

The strengths of the study include, well-defined methodology including radiosynthesis and imaging validation attempts, evaluation of the specificity of the radiotracer, comparison to previously evaluated radiotracers, multi-time point imaging, and sufficient methodological detail to reproduce the studies. The major findings of the study are: (1) Higher uptake of [11C]J147 in activated BAT compared to [18F]FDG and [18F]BCPP-EF; (2) ATP synthase expression correlated with interscapular BAT activation and visceral WAT activation, validating it as a potential biomarker; and (3) Compared to [18F]FDG, superior visualization of other BAT fat depots, as identified in reference 11 using a lipid based imaging agent, including supraspinal, axillary, and infrascapular; and acknowledgement of limitations in the discussion.

There are major weaknesses and concerns that should be addressed before this radiotracer can considered for further evaluation as a potential novel imaging tool in humans:
(1) The experimental design lacks mechanistic studies to determine whether [11C]J147 directly binds ATP synthase or if other mitochondrial proteins contribute to the uptake;

(2) The sample sizes for PET imaging and biodistribution studies (n=3/group) are small which reduces the statistical power and increases the risk of type II errors. Limited ability to generalize findings, especially across BAT depots; The in vitro siRNA knockdown studies also had a small sample size (n=3) for qPCR and radiotracer uptake studies, but the effects sizes were large, reducing impact of variability;

(3) While in general the validations studies support the PET imaging there are concerns that should be addressed to strengthen the manuscript and the potential of ATP synthase as an imaging biomarker: (a) No direct enzyme activity assay was performed, correlation coefficients between ATP5A expression and [11C]J147 SUV were not provided, and there was no quantitative western blot validation of ATP5A expression; (b) In vitro autoradiography only measures binding, not enzyme activity, there was no comparison to another ATP synthase inhibitor or competitor to confirm selectivity and no ATP synthase knockout models were used to validate loss of tracer binding; (c) It remains unclear whether the results obtained in PC3 prostate cancer cells would translate to BAT function; (d) No complementary overexpression studies were performed to show increased uptake with higher ATP5A levels and western blot validation of ATP5A knockdown was missing; (e) In the biodistribution studies there is confirmation of PET imaging but lack of direct enzymatic activity validation. The missing enzyme activity assays in the validation methods would have provided direct evidence that ATP synthase function, not just expression, correlates with the PET tracer uptake. This could help distinguish between ATP synthase as an active metabolic regulator versus a passive biomarker and would confirm that [11C]J147 is tracking ATP production, not just ATP synthase protein levels.

(4) The authors primarily used Student's t-test (unpaired, two tailed) to compare tracer uptake between activated and non-activated BAT, Autoradiography and siRNA knockdown studies, and SUV differences between [11C]J147 and [18F]FDG. There was no multiple comparisons correction (acknowledged in Figure 1), no power analysis reported. ANOVA with post-hoc correction would have been more appropriate for multiple comparisons across fat depots;

(5) The impact of anesthesia used in the PET imaging is not optimally considered. The authors report using isoflurane anesthesia for all PET/CT imaging, giving internal control but its impact was not addressed. There is no discussion of the potential suppressive effects of isoflurane on BAT activity. It is known to blunt BAT activation by reducing norepinephrine signaling. Alternatives such as ketamine-xylazine that better preserve BAT function were not considered. While activated and non-activated groups were treated equally this does not rule out the possibility that overall tracer uptake was lower than it would be under awake conditions. This is particularly important given that [18F]FDG uptake is known to be sensitive to anesthesia and in light of the low SUVs reported in some of the figures (~3 or even less than 1 in some older mice). In clinical practice, it is quite possible that such low values would not allow the radiologist to confidently identify these depots above surrounding background tissue.

There are other minor issues that should be addressed. Reference 11 is not a mitochondrial potential targeting tracer as stated in the text. It is a CD36 substrate and marker of lipid oxidation but cannot be metabolized. Its uptake is related to the use of this pathway for fuel. There are multiple types of "SUV". The authors do not state which SUV they are reporting, e.g. mean, max, peak etc. The PMOD program is not reconstruction software as the authors imply on line 469. They should state how the images were reconstructed and subsequent analysis was performed using PMOD.

Version 1:

Reviewer comments:

Reviewer #1

(Remarks to the Author)
The authors have addressed most concerns. In the revised manuscript, the specificity of [11C]J147 has now been comprehensively validated through both in vitro autoradiography and in vivo PET imaging studies. Importantly, the imaging performance of [11C]J147 has been further evaluated in metabolic disease models, including STAM and GLP-1 treatment, which substantially strengthens the translational relevance of the study. Overall, the experimental design is now more comprehensive, and the data is more convincing.
I have only a few minor comments that should be addressed prior to publication:
1. To facilitate a clearer comparison between the radiotracer and its blocking agent, I recommend that the chemical structure of CJ1-34 be included in the Supplementary Materials. This would help readers better understand the structural relationship and differences between the tracer and the blocker.

2. In the PET imaging studies, after CJ1-34 blocking, tracer uptake appears to continue increasing and approaches baseline levels at later time points. The authors are encouraged to discuss possible explanations for this observation, including incomplete receptor occupancy or reversible binding kinetics, contributions from non-specific uptake, tracer metabolism or radiometabolite accumulation. More discussion of these potential mechanisms would improve the interpretation of the blocking results.

Reviewer #2

(Remarks to the Author)
I co-reviewed this manuscript with one of the reviewers who provided the listed reports. This is part of the Nature Communications initiative to facilitate training in peer review and to provide appropriate recognition for Early Career Researchers who co-review manuscripts.

Reviewer #3

(Remarks to the Author)
Thank the authors for making serious attempts to address the limitations posed in the review. My concerns have been adequately addressed within a reasonable way. The rather limited blocking of the [11C]J174 remains disappointing but the authors offer a plausible explanation, though other possibilities exist such as affinity mismatch between the blockers and their [11C]J147, some degree of non-specific uptake of [11C]J147, or levels of occupancy on the ATPase by endogenous ligands.

Open Access This Peer Review File is licensed under a Creative Commons Attribution 4.0 International License, which permits use, sharing, adaptation, distribution and reproduction in any medium or format, as long as you give appropriate credit to the original author(s) and the source, provide a link to the Creative Commons license, and indicate if changes were made.
In cases where reviewers are anonymous, credit should be given to 'Anonymous Referee' and the source.
The images or other third party material in this Peer Review File are included in the article's Creative Commons license, unless indicated otherwise in a credit line to the material. If material is not included in the article's Creative Commons license and your intended use is not permitted by statutory regulation or exceeds the permitted use, you will need to obtain permission directly from the copyright holder.
To view a copy of this license, visit https://creativecommons.org/licenses/by/4.0/

Dear Reviewers,

Thank you for your interest in our work, your positive feedback, your careful reading of the manuscript, and your helpful suggestions for improvement. Please see our point-by-point responses to the requests below:

REVIEWER COMMENTS

Reviewer #1 (Remarks to the Author):

This paper by Jie et al. describes the evaluation of ATP synthase for imaging activated and non-activated adipose tissues using the radioligand [11C]J147. Although [11C]J147 is not a novel tracer, as its synthesis and potential application in Alzheimer's disease imaging have been previously reported (Bioorg. Med. Chem. Lett. 2013, 23(2):524-527. doi: 10.1016/j.bmcl.2012.11.031.), the authors are the first to apply it to BAT imaging. Such an approach could be valuable for PET-based studies on adipose tissue metabolism and drug development for metabolic disorders. However, the in vitro autoradiography studies and the in vivo PET evaluation of [11C]J147 remain incomplete. Specifically, further validation in pathological models of metabolic diseases and direct comparison with additional established BAT imaging tracers would strengthen the conclusions.

We appreciate reviewer's encouraging comments and constructive suggestions. In the revised manuscript: 1) different blockers were included in in vitro autoradiography experiments; 2) Competition binding studies were performed using J147 and another ATP inhibitor CJ1-34 to investigate the in vivo specificity of [^{11}C]J147 and 3) the potential applications of the target and radiotracer in diagnosis and drug discovery were assessed.

General Comments:

1. It would be helpful for the authors to provide more information on the selectivity and specific binding of [11C]J147. The in vitro autoradiography experiment only tested the self-blocking effect. What about the blocking effects of other selective ATP synthase inhibitors? Different blockers should also be used in the in vivo PET imaging to verify specific binding.

Thank you for the insightful comment. We have added additional in vitro autoradiography blocking studies with ATP synthase inhibitor CJ1-34, which was newly synthesized in our research group, and mitochondrial complex I inhibitor BCPP-EF for evaluating its selectivity. Following results were included in the revised manuscript: "CJ1-34 competed with [^{11}C]J147 binding in a concentration-dependent manner, while no significant blocking was found for BCPP-EF (P = 0.973) (Fig. 1e). Representative radioautographs are shown in Extended Data Figure 1."

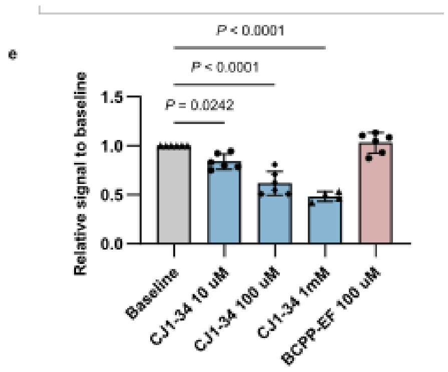

Fig. 1e, Semiquantification of autoradiographs from interscapular BAT slices of CL316,243-treated mice incubated with [^{11}C]J147 (3–5 nM; baseline condition) and co-incubated with indicated concentrations of CJ1-34 or BCPP-EF (100 µM, served as a negative control) (n = 4-6 for each).

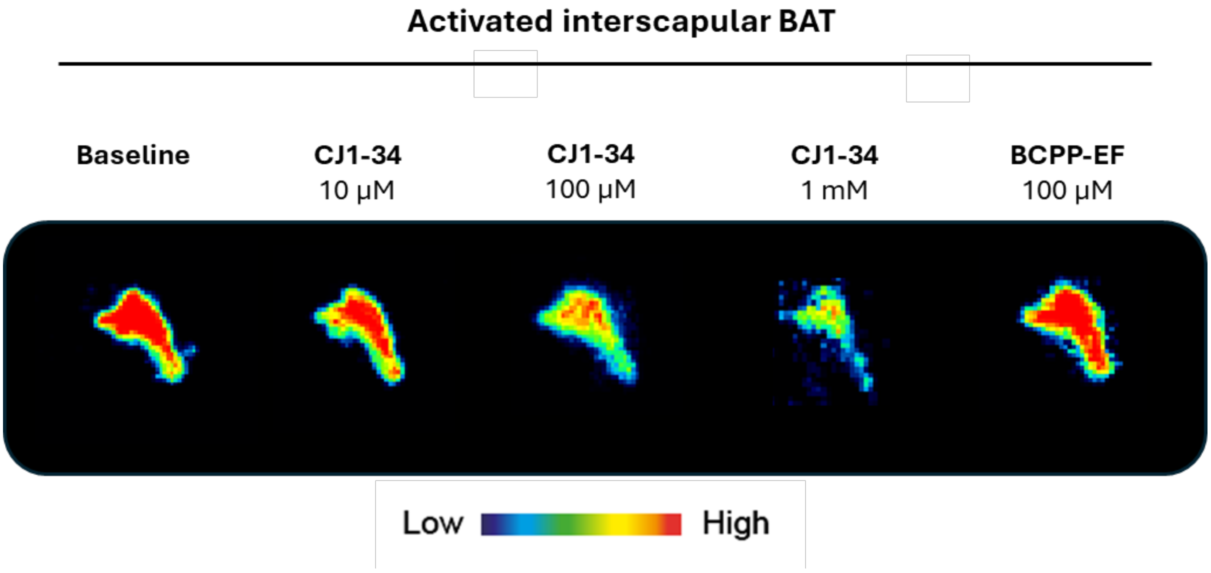

Extended Data Fig. 1. **Representative *in vitro* autoradiograms of** [^{11}C]J147 obtained from CL316,243-treated Balb/c mice interscapular BAT sections under baseline condition, after addition of different concentration of CJ1-34 (10 µM, 100 µM, and 1 mM, a compound targeting the ATP synthase) and after addition of 100 µM BCPP-EF (a compound targeting specifically the mitochondrial complex I).

To further assess in vivo specificity, competition (blocking) studies were conducted using excess non-radioactive J147 and another ATP synthase inhibitor compound CJ1-34. In both blocking conditions, a reduction in tracer uptake was observed in brown adipose tissues (BATs) at 70 min post-injection in the biodistribution studies (see Figure 3h-i), indicating target-mediated binding in vivo. The extent of the blocking effect was modest, which is likely related to the exceptionally high abundance of ATP synthase in BATs and the consequent difficulty in achieving substantial target occupancy with a single bolus administration of inhibitor. Under these conditions, higher doses or prolonged exposure (e.g., continuous infusion) would be expected to produce more pronounced displacement. Notably, PET time–activity curve under CJ1-34 blocking conditions (Fig. S6) shows a more pronounced reduction in tracer uptake at earlier time points (15-20 min post-injection), indicating that competitive inhibition is more

evident during the initial uptake phase and becomes less apparent at later time points used for ex vivo biodistribution (70 min). Together, these data support a specific, target-associated uptake in BATs, while also highlighting the limitations of bolus blocking strategies for highly abundant mitochondrial targets such as ATP synthase.

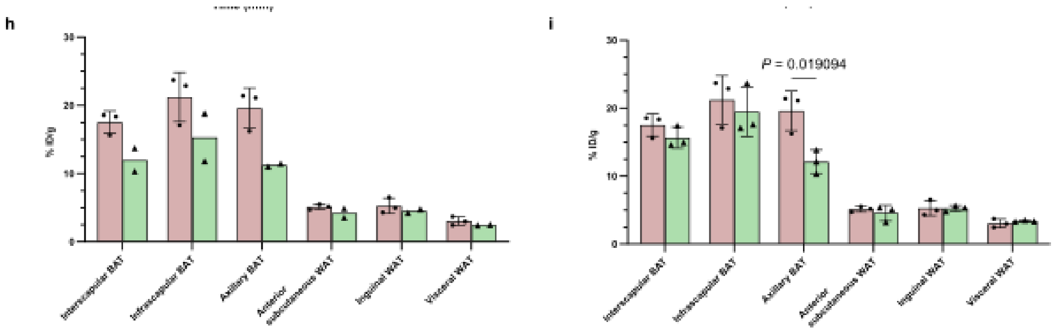

Fig. 3 h,i, Biodistribution of [11C]J147 in CL316,243-treated Balb/c mice (n=3) reported as %ID/g (red bars). For blocking conditions, animals were injected with J147 (**h**; i.v., 4 mg/kg, n=2, green bars) or CJ1-34 (**i**; i.v., 4 mg/kg, n=3, green bars) 3 minutes before [11C]J147 injection. Data for n = 3 are represented as mean ± s.d. Comparison between baseline and blocking by unpaired t-tests with Welch correction and correction for multiple comparisons using Holm-Šídák method.

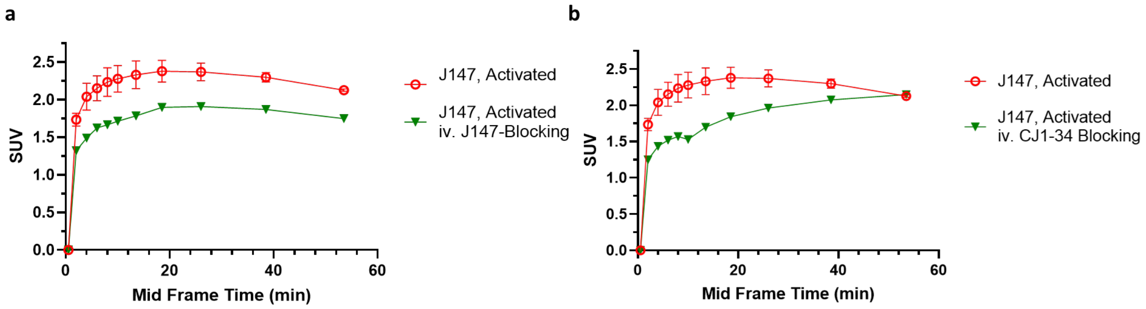

Fig. S6. Time-activity curves (TACs) of interscapular BAT from PET studies in mice. TACs represent accumulation of [11C]J147 in infrascapular BAT as a function of time for baseline (n=3) and blocking scan with J147 (n=1, iv. 4 mg/kg) (a) or CJ1-34 (n=1, iv. 4 mg/kg) (b) pretreatment 3 min before radiotracer injection.

2. The authors should use literature or experimental data to explain the expression levels of BAT and WAT in mice of different ages, and provide an explanation for the selection of 14-15 week-old and 12-13 month-old mice for the PET imaging study.

Literatures about the changes of BAT level at different ages were included in the revised manuscript (ref 49, 50). We performed additional experiments to investigate the uptake of [11C]J147 and [18F]BCPP-EF in mice at two different age groups (14-15 weeks old and 51-55 weeks old). In vivo PET imaging revealed that [11C]J147 has higher BAT uptake in younger mice than older mice, while [18F]BCPP-EF could not visualise BAT in mice aged 14–15 or 51–55 weeks. (Fig. 3b-e) Our findings suggest that ATP synthase is a more promising target for monitoring brown adipose tissue changes than mitochondrial complex I.

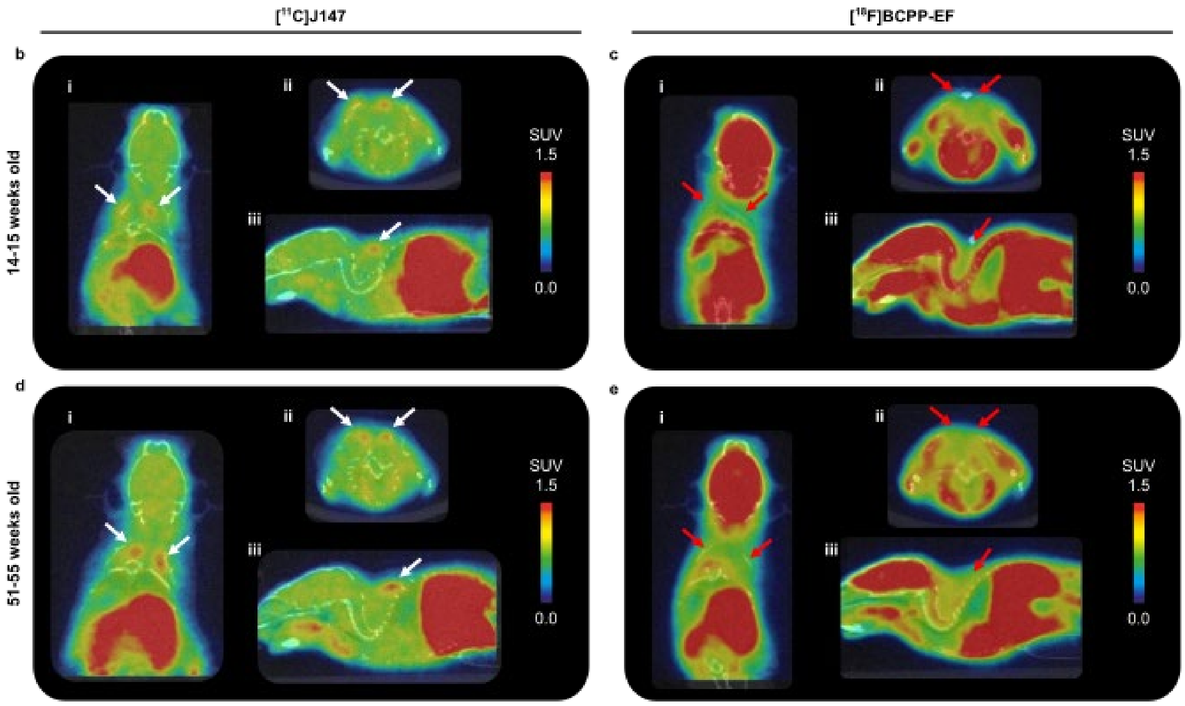

Fig 3. b-e, Representative averaged images (time frame 0-60 min) of interscapular BAT (white arrows or red arrows if not visible) imaged with [^{11}C]J147 or [^{18}F]BCPP-EF depicted in coronal (i), transverse (ii), and sagittal plane (iii) of C57BL/6;C3H mice.

3. What is the imaging effect of [18F]BCPP-EF and [11C]J147 in younger mice? Focusing on 12-13 month-old mice may limit the broader understanding of these tracers, and including data from younger mice could provide a more comprehensive comparison.

To address the reviewer's comment, we have added PET studies using [^{11}C]J147 and [^{18}F]BCPP-EF in mice at two different age groups (14-15 weeks old and 51-55 weeks old). The results were presented in Fig 3b-e.

4. How is the position and volume of interscapular BAT in mice? It might be useful for the authors to explore ex vivo autoradiography and H&E staining to demonstrate that [11C]J147 uptake occurs throughout the entire BAT, rather than being limited to a localized region.

We thank reviewer for the comment. In the revised manuscript, we provided an *in vivo* dynamic [^{11}C]J147 PET imaging for visualisation of interscapular BAT at different time frames. The imaging is presented in Figure S7 of the supplementary material. The information on the extraction, position, weight and volume of interscapular BAT in the mice investigated in this study are included in Fig S10-11 and S20-21 of the supplementary information.

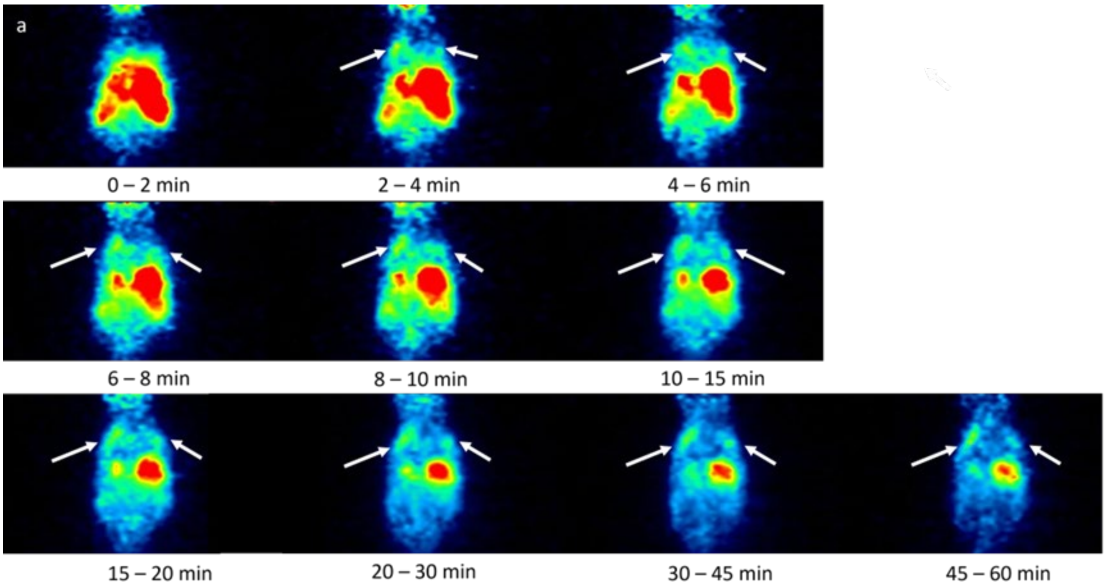

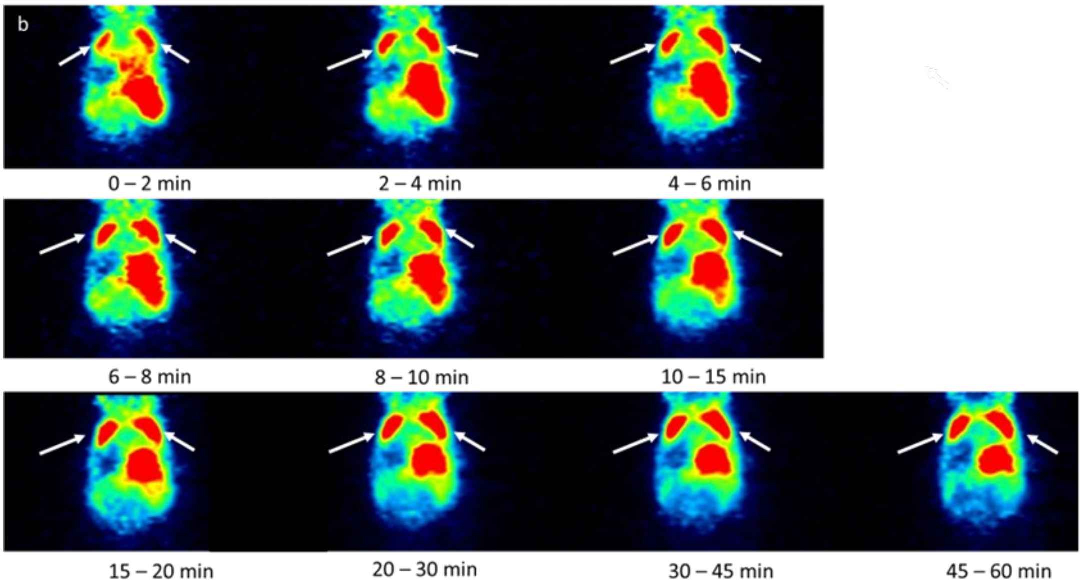

Figure S7. Representative PET images of sagittal mouse body sections at different time frames after intravenous injection of [^{11}C]J147 in vehicle-treated mice (**a**) and [^{11}C]J147 in CL316,243-treated mice (**b**). White arrows indicate interscapular BAT depots over time. SUV: 0-2.5.

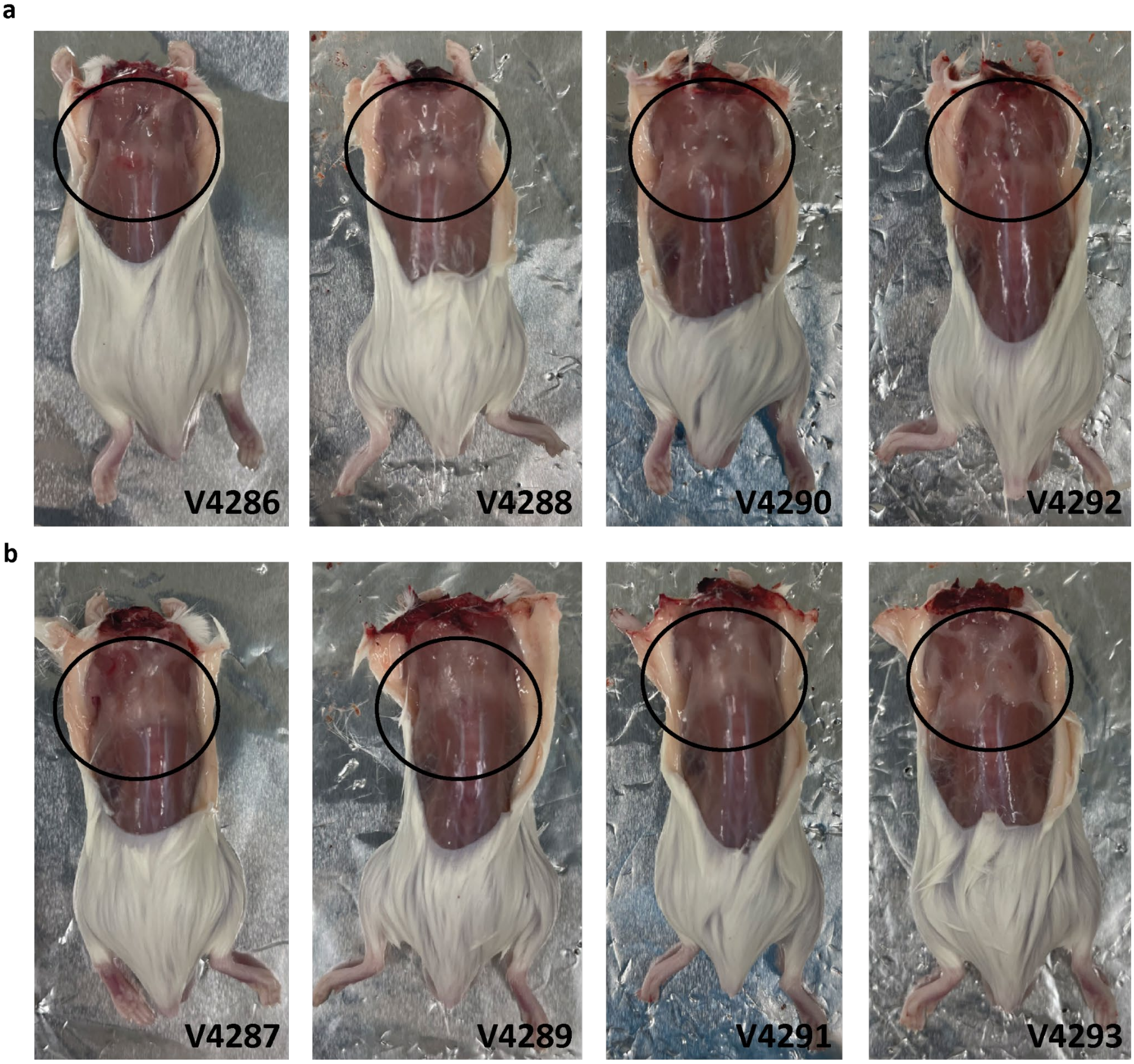

Fig. S10. Pictures show the location and size of the classical brown adipose tissues (indicated by the black circle) located on the back of the animals. (**a**) Panel shows Balb/c mice injected with CL316,243 after decapitation, skin is cut and removed to expose the adipose tissues below. (**b**) Panel show Balb/c mice injected with vehicle (PBS). No visible differences were observed.

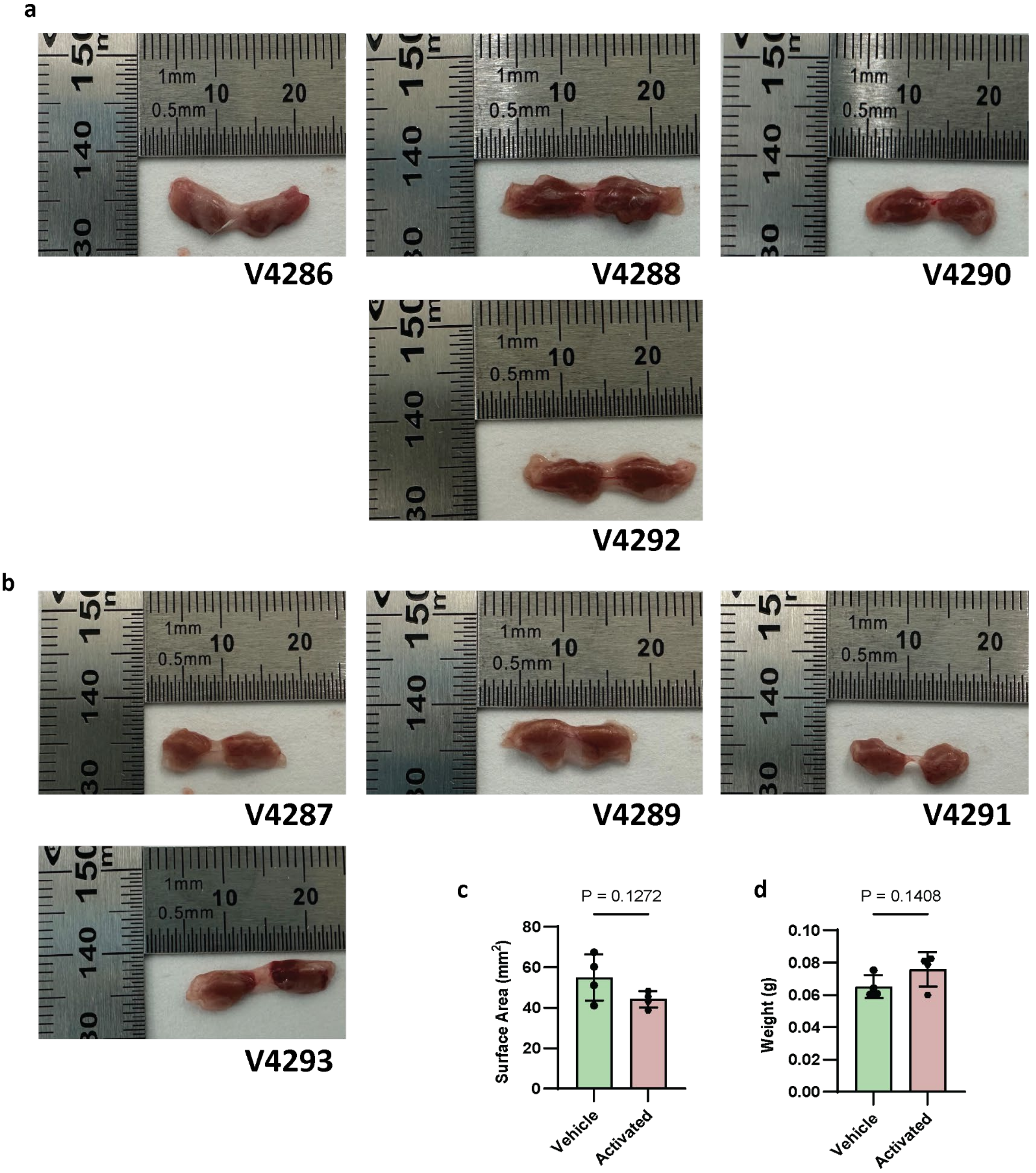

Fig. S11. Classical brown fat depots from mice injected with CL316,243 (**a**) and vehicle (**b**) were dissected and measured. (**c**) No significant difference in classical brown fat adipose tissue surface area was detected between treatment groups. (**d**) No significant difference in classical brown adipose depot weight was observed between treatment groups. Data are presented as mean ± s.d. Two-tailed Student's *t*-test with Welch correction (**c,d**).

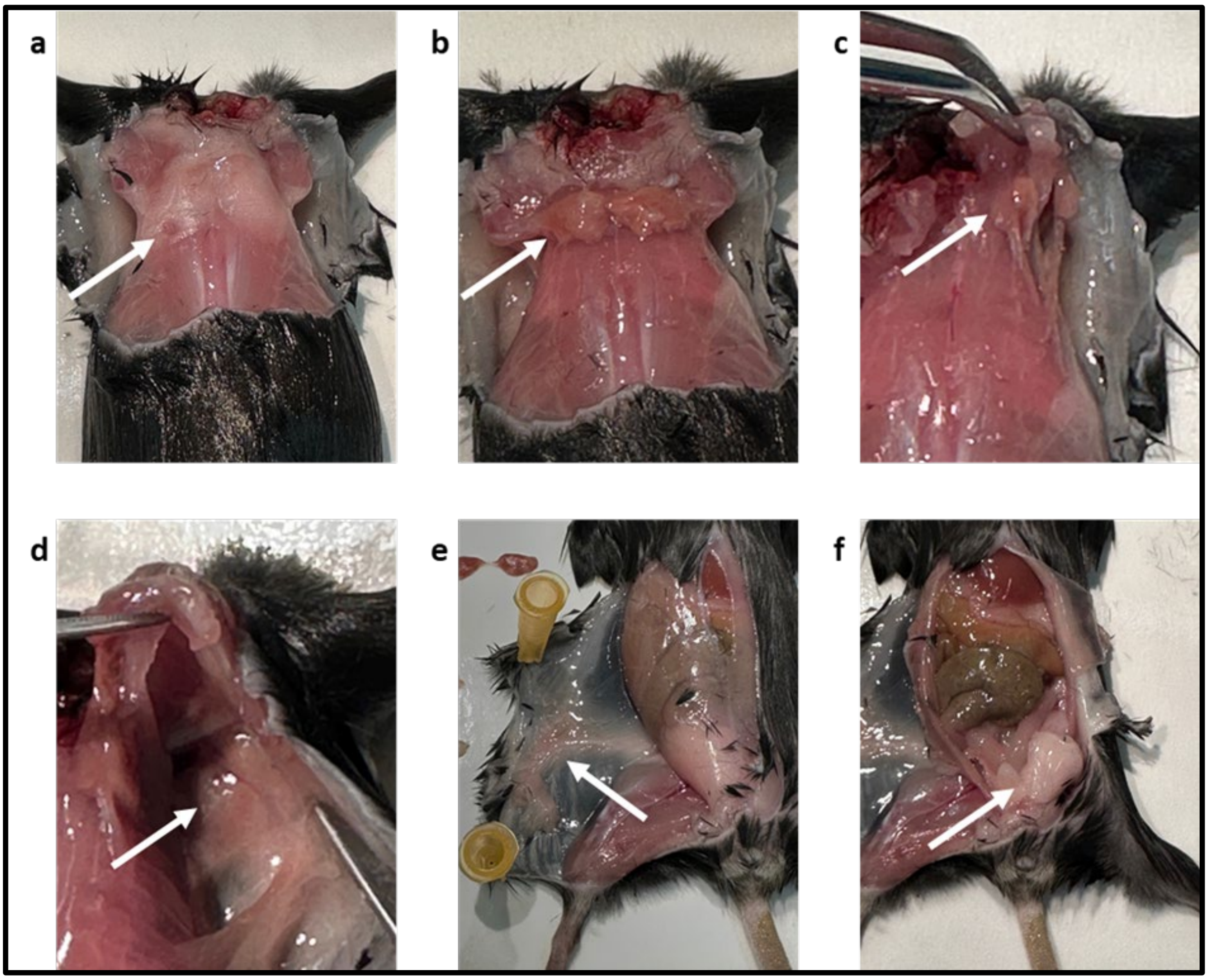

Figure S20. Dissection of adipose tissue depots in mice. To isolate adipose tissue depots, mice are first euthanized by decapitation following approval and ethical guidelines. The animal is placed in a prone position, and the skin is incised along the midline from the middle back region to the neck to expose the interscapular BAT surrounded by anterior subcutaneous WAT (**a**, white arrow). The anterior subcutaneous WAT is carefully excised, avoiding contamination with adjacent muscle or fat. The interscapular BAT (white arrow) can be visualized (**b**) as a distinct tissue located between the shoulder blades, overlaying the dorsal surface of the upper thoracic spine. The infrascapular BAT can be found under the shoulder plate, it appears as a dark-reddish-brown tissue. Shoulder plate is lifted and the infrascapular BAT, indicated with a white arrow, is sampled (**c**). The axillary BAT depots are located lateral to the upper thoracic spine and close to the junction of the forelimb and thorax. These depots appear as small, darker brown tissues compared to surrounding white and fat and muscle, and are positioned medially to the forelimb, indicated by a white arrow (**d**). The animal is then placed in a supine position, and the skin is incised along the midline from the lower abdomen to the neck. Skin is removed from internal organs and fixed to the dissection surface. Inguinal WAT (**e**, indicated by a white arrow) is located subcutaneously along the lower abdomen flanks, lateral to the hindlimbs, and is carefully separated from the skin tissue using forceps and scissors. Visceral WAT (**f**) is found within the peritoneal cavity adjacent to the testes, in males, and is dissected by gently lifting them and isolating the surrounding fat. In the picture is indicated by a white arrow.

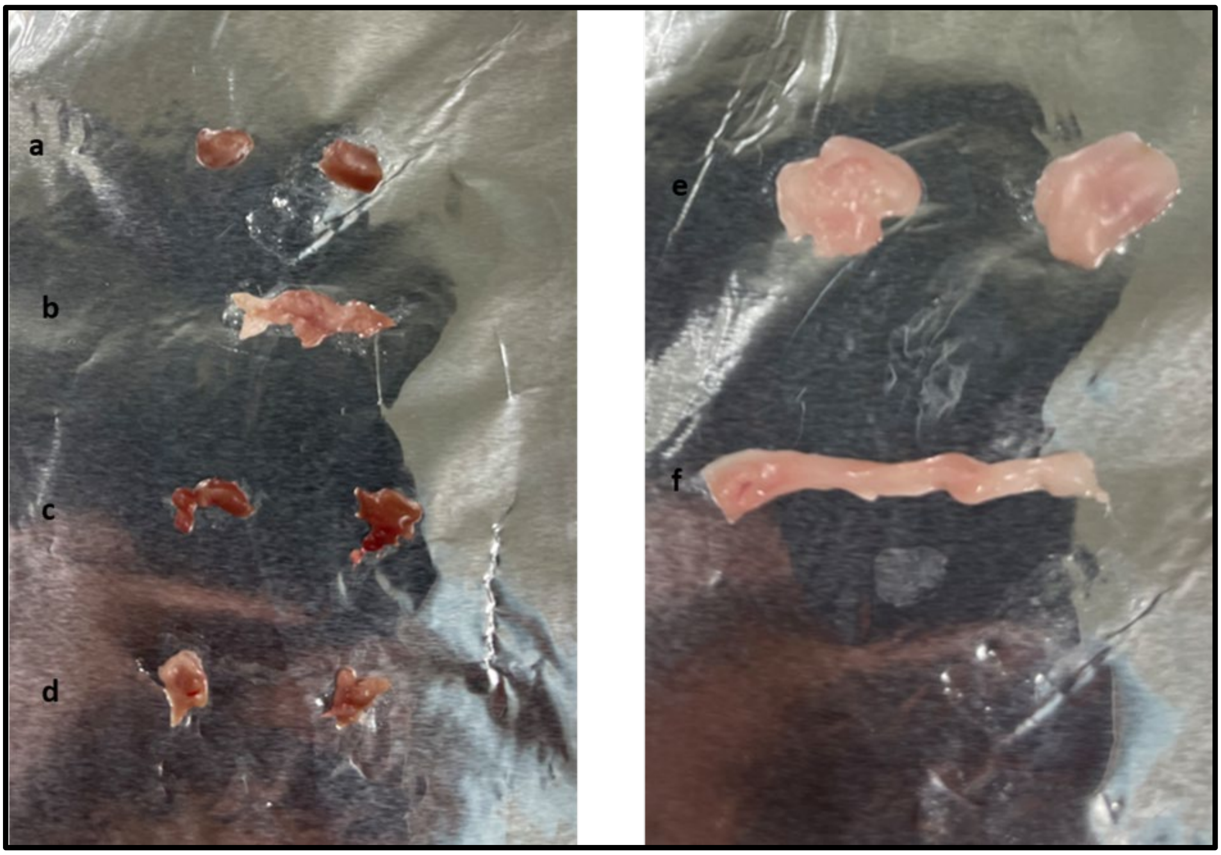

Figure S21. Dissection of adipose tissue depots in mice. The different fat depots dissected in this study are shown in this figure. Interscapular BAT, also known as the classical BAT (**a**). Anterior subcutaneous WAT (**b**). Infrascapular BAT (**c**). Axillary BAT (**d**). Visceral WAT (**e**). Inguinal WAT from one side of the body (**f**).

5. In line 256, authors should add statement on the attributes and limitations of these PET-ligands targeting glucose metabolism, translocate protein and mitochondrial membrane.

The following content was included and added to the revised manuscript.

"Despite this range of tracers, imaging adipose tissue remains challenging. Whilst tracers such as [^{18}F]fmPBR28-d_2 and [^{18}F]BODIPY1 could detect interscapular BAT in thermoneutral and cold-exposed conditions[13,14], others like [^{18}F]FEPPA and [^{18}F]BCPP-EF displayed no increased uptake in β_3-adrenergic agonist-activated BAT, or even demonstrated decreased uptake after activation of adipose tissue via cold exposure in the case of [^{18}F]FBnTP[10,15,16]. The current gold standard, [^{18}F]FDG, targets glucose metabolism but has limited specificity in distinguishing increased tracer uptake arising from adipocyte metabolic activity versus neuroinflammatory processes. Consequently, it cannot reliably or conclusively detect the presence or absence of BAT in some cases[17–19]."

And in the discussion part: "Recent studies indicate that, in addition to classical thermogenesis mediated by UCP1-driven uncoupled respiration, certain thermogenic adipocytes can generate heat through ATP-

dependent futile cycles[30,45,46]. This mechanism has been reported in both brown and white adipose tissues where beige adipocytes are recruited following cold exposure or β3-adrenergic activation. For example, ATP synthase levels were reported to increase in white adipose depots following a 7-day CL316,243 treatment[47]. Moreover, two independent studies identified populations of adipocytes in inguinal fat rely on futile cycle-dependent thermogenesis under cold exposure[31,48]. Notably, currently available PET imaging targets, including glucose metabolism, translocate protein and mitochondrial membrane potential among others, are not directly monitoring ATP synthase-related processes and thus are not well-suited for detecting adipocytes engaged in ATP-dependent futile cycles."

6. It would be helpful for the authors to provide more detailed information in the introduction about the relationship between ATP synthase and BAT, including how they are regulated, the potential metabolic pathways they are involved in, and their possible roles in metabolic diseases.

We thank reviewer for this helpful suggestion. In response, we have expanded the Introduction to provide additional background on the relationship between ATP synthase and brown adipose tissue (BAT), including their regulation and involvement in mitochondrial and metabolic pathways. The following text has been added to the Introduction:

"Beyond the well-established upregulation of UCP1 following BAT activation (e.g. cold stimulation), increases in BAT mass and ATP synthase content have been reported[23]. Several studies have further demonstrated that UCP1 increase is accompanied by the upregulation of mitochondrial genes and proteins involved in the respiratory chain, including ATP synthase as well as markers of mitochondrial biogenesis[23–27]. Consistent with these results, ATP synthase upregulation has also been reported in UCP1-recombinant yeast cells, with a 3.6-fold increase observed in strains exhibiting high UCP1 expression[28]. These findings support a positive association between BAT activation, UCP1 expression and increased ATP synthase abundance."

In addition, to address the potential roles of ATP synthase in metabolic disease, we have added new discussion text (highlighted in yellow in the revised manuscript) that interprets our findings from the "Evaluating the potential application of [^{11}C]J147 in the STAM model and GLP1 treatment".

7. For [11C]J147, its application in animal models of metabolic diseases, such as obesity and diabetes, should be evaluated to assess its imaging effects under pathological conditions. Additionally, further investigation is required to explore its imaging performance and pharmacokinetic properties in non-human primates (NHPs).

To address the reviewer's comment, we have added the STAM animals to investigate ATP synthase as a valuable imaging target in metabolic disease and biodistribution of liraglutide treatment mice to assess the potential application of [^{11}C]J147 in drug discovery. A new section of "Evaluating the potential application of [^{11}C]J147 in the STAM model and GLP1 treatment" and figure 7 are added in the revised

manuscript. We agree with the reviewer that for the future human application, non-human primates (NHPs) are required to explore its imaging performance and pharmacokinetic properties.

Evaluating the potential application of [^{11}C]J147 in the STAM model and GLP1 treatment

To further investigate the relevance of ATP synthase as a potential imaging target in metabolic disease, we studied the ATP5A expression in various adipose tissues of a mouse model of diabetes, namely the mouse model of non-alcohol steatohepatitis and hepatocellular carcinoma (STAM mice)[43]. ATP5A expression in inguinal WAT was significantly reduced compared with controls at 8 weeks (P = 0.043) and showed a further decrease at 17 weeks of age (P = 0.0104) by Western blotting studies (Fig. 7a,c; Extended Fig. 5). Similar results were found by immunohistochemistry at 17 weeks of age (Fig 7e). In contrast, ATP5A expression in interscapular BAT did not differ from age-matched control mice at either 8 or 17 weeks of age, as assessed by both Western blotting and immunohistochemistry (Fig. 7b,d,e).

We furthermore investigated whether ATP synthase has potential as an imaging target for therapy monitoring in metabolic disease. Therapeutic compounds that activate the glucagon-like peptide-1 (GLP-1) receptor, including liraglutide, are increasingly recognised for their benefits in diabetes and obesity management[44]. To assess how liraglutide influences ATP synthase expression under physiological conditions, we conducted biodistribution studies in liraglutide-treated (0.2 mg/kg, s.c., at 24h and 4h), 12-week-old female Balb/c mice. At 70 minutes p.i., [^{11}C]J147 uptake was largely comparable to the control across most adipose tissues. However, interscapular BAT exhibited a significant increase in tracer accumulation following this short liraglutide exposure (P = 0.044; Fig. 7f). Moreover, significantly increased brain uptake of [^{11}C]J147 (P = 0.0028; Supplementary Figure S16) was observed in liraglutide treated mice compared to the controls.

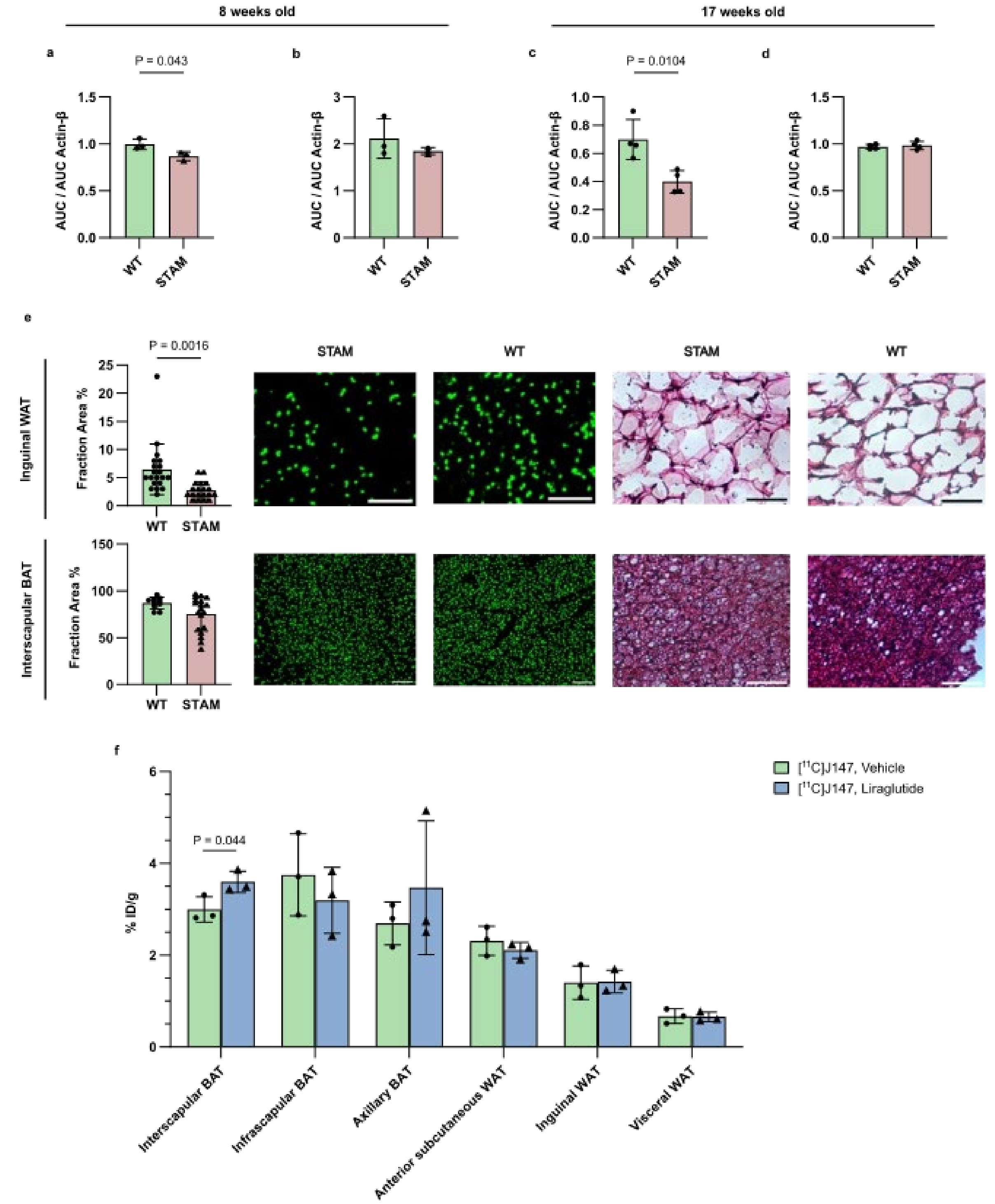

Fig. 7. **a-d,** Semiquantification of ATP5A expression via western blot in inguinal WAT (**a**,**c**) and interscapular BAT (**b**,**d**) in 8 weeks old (**a**,**b**; n=3 per group) or 17 weeks old (**c**,**d**; n=4 per group) STAM and control mice. **e,** Semiquantification of ATP5A via immunohistochemistry in inguinal WAT and interscapular BAT and H&E staining (scale bar 100 µm) in STAM or control 17 weeks mice (n = 3-4 per group). **f,** Biodistribution of [^{11}C]J147 in adipose tissues in vehicle-treated (n=3) and liraglutide-treated Balb/c mice (n=3) reported as % injected dose/weight. Data are represented as mean ± s.d., two-tailed Student's *t*-test with Welch correction.

Specific Comments:

1. In line 85 "The molar activities of [11C]J147 were 257 ± 154 GBq/µmol (n =19) at the end of synthesis."

Please provide calibration curves for determining molar activity and provide the molar activities of [18F]BCPP-EF in manuscript.

The calibration curves of [^{11}C]J147 and [^{18}F]BCPP-EF are added in the supplementary information Fig S19. The molar activities are now mentioned in the result part and in the detailed experimental part in the methods of supplementary information.

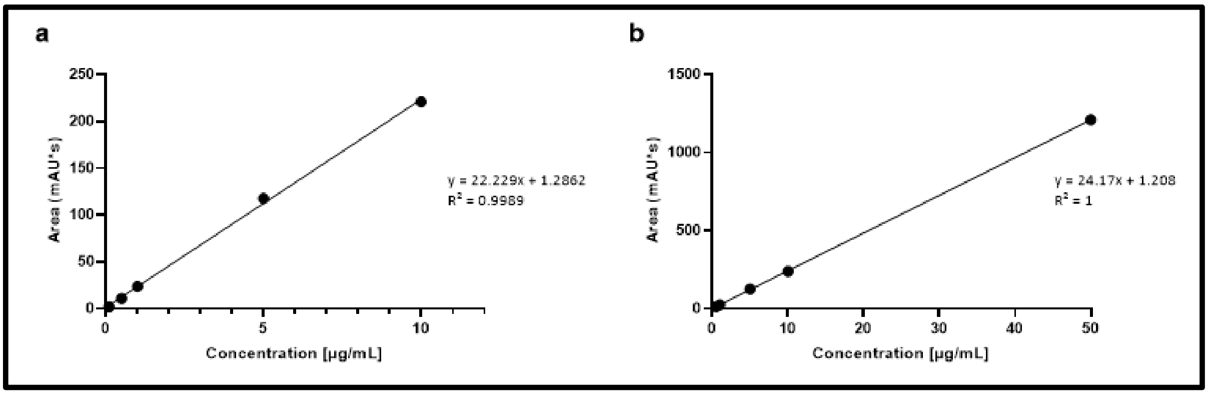

Fig. S19. Calibration curve of [^{11}C]J147 (a) and [^{18}F]BCPP-EF (b) for determining their molar activity.

2. In line 90, lack of blocking conditions (J147: 100 μM). The author uses such a high concentration because lower concentrations do not have a blocking effect?

We thank reviewer for this question. The target ATP synthase is indeed an abundant target. In our additional autoradiography studies, different concentration of CJ1-34 from 10 μM, 100 μM, and 1 mM were used and a concentration-dependent competition binding effect was observed.

3. In line 101, why was siRNA used to transfect PC-3 cells? Could the author provide a reason for not using a standard tool cell line?

We appreciate the reviewer's suggestion regarding tool cell lines. We chose the siRNA transient knockdown approach rather than generating stable knockout or knockdown cell lines for the following reasons:

1). ATP5A is an essential gene: Complete and stable knockout of ATP5A would likely be lethal to cells, as ATP synthase is critical for cellular energy production. Several studies have shown that ATP5A knockout results in severe growth defects or cell death (PMID: 17387143). Therefore, a transient knockdown approach allows us to achieve partial reduction of ATP5A while maintaining cell viability for functional studies.

2). Transient knockdown is appropriate for validation: To validate tracer specificity, transient siRNA knockdown (achieving 60-80% reduction in ATP5A expression) is sufficient to demonstrate the correlation between ATP5A expression levels and [^{11}C]J147 uptake.

3). Time and resource considerations: The generation and validation of stable cell lines (knockout or knockdown) would require several months, and given ATP5A's essential role, might not be feasible without compensatory mechanisms that could confound interpretation.

4. In figure 1, scale for autoradiography can be listed with real-time numeric (instead of low and high).

We have replaced low and high with 0.0 and 1.0.

5. In line 137, why are Balb/c mice used for immunofluorescence staining and C57BL6 mice used for PET imaging? Please explain the reason.

We thank reviewer for this question. In our studies, C57BL/6;C3H mice was used for comparing the uptake of [^{11}C]J147 and [^{18}F]BCPP-EF in mice at two different age groups. All other PET/CT experiments related to vehicle- or CL316,243-treatment were conducted in Balb/c mice, and the BAT and WAT slices from the same brand of mice were analysed by immunohistochemistry. Balb/c mice were used as a model for fat tissue activation; we had also previously developed this method in our lab.

6. In figure 2b-d and 2g-2l, the author should provide PET-CT or PET-MRI fusion images to better help identify the locations of organs and BAT.

We understand that reviewer means Figure 3b-e, see above in answers to question 2, we have replaced the PET-only images with PET-CT images in Figure 3.

7. In section "[11C]J147 detects interscapular BAT in healthy mice in vivo", the author could provide dynamic time-activity curves (TAC) of different brain regions in mice, given that [11C]J147 is used as an AD imaging tracer. Investigating the uptake and distribution in the brain is equally important, and this information could be included in the supplementary materials.

We thank reviewer for this thoughtful suggestion. Time-activity curves of [^{11}C]J147 in two different aged mice brain were included in the supplementary information (Fig S4). Consistent with the results in BAT, higher [^{11}C]J147 brain uptake was observed in younger mice than older mice.

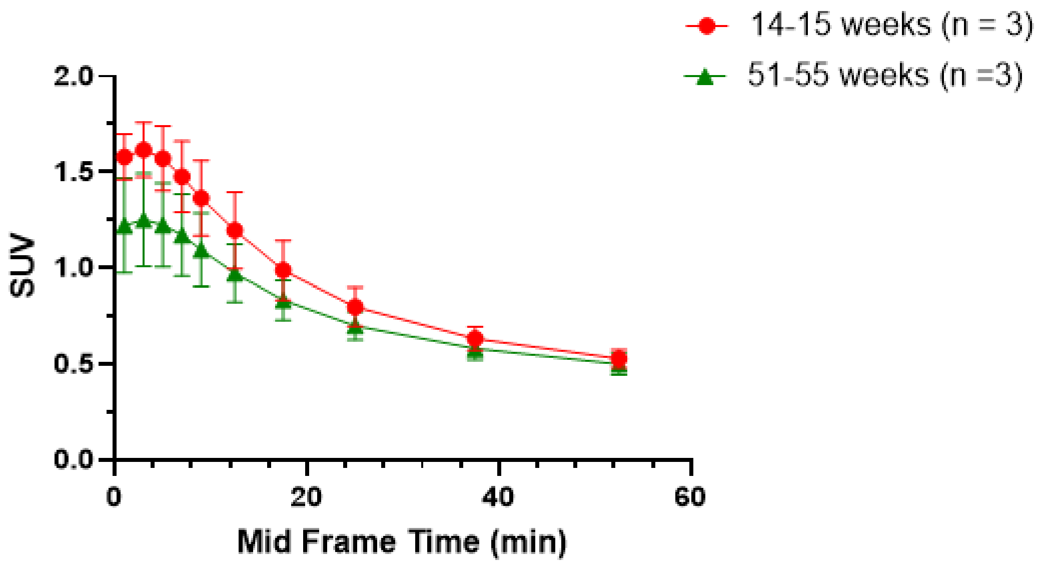

Fig. S4. Time activity curves (TACs) of [^{11}C]J147 in the whole brain in 14-15 weeks old (n=3) and 51-55 weeks old (n=3) C57BL/6;C3H mice.

8. In figure 4, the author performed a 0-60 minute PET scan, and it can be seen that [11C]J147 has a relatively slow wash-out. Were PET scans done at 90 minutes or 120 minutes?

Thank you for the comment. [^{11}C]J147 has indeed shown a relatively slow wash-out in adipose tissue, but a normal kinetics in the brain (see Fig S4 in the Supplementary materials). We have included this information in our revised manuscript.

9. In line 313 "Female C57BL/6;C3H mice (n=3, 14-15 weeks old, 19-25 g and n=4, 51.0-52.9 weeks old, 22-28 g)were supplied by Jackson laboratory and female Balb/c (n=6, 14-15 weeks old, 19-22 g) were supplied by Charles River (Sulzberg, Germany)". The author should clarify the reason for choosing female mice for the experimental design in the manuscript. Were male mice considered? and if so, what factors led to the selection of female mice? Additionally, is the BAT expression level consistent between male and female mice?

To address the reviewer's comment, we have included male mice (n=12 in total) in our PET and biodistribution studies of using [^{11}C]J147 and [^{18}F]FDG in vehicle- and CL316,243-treated mice. The new results are included in Figures 4 e-f, Figure 5, Figure 6. In the supplementary information Figure S8-9, averaged SUVs of [^{11}C]J147 for the interscapular BAT of the vehicle-treated female and male groups were compared and indicating there is no significant difference of [^{11}C]J147 uptake between male and female mice.

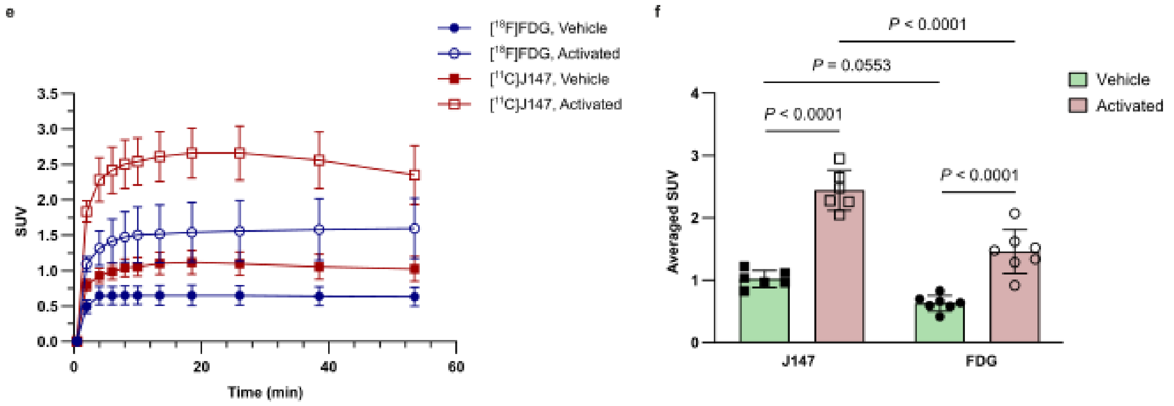

Fig. 4. e, Time activity curves of [11C]J147 (n=6 per group) and [18F]FDG (n=7 per group) in interscapular BAT in vehicle-treated and CL316,243-treated groups. **f,** Averaged SUV of the vehicle-treated and CL316,243-treated groups. Data are represented as mean ± s.d. one-way ANOVA with Tukey's post-hoc correction for multiple comparison.

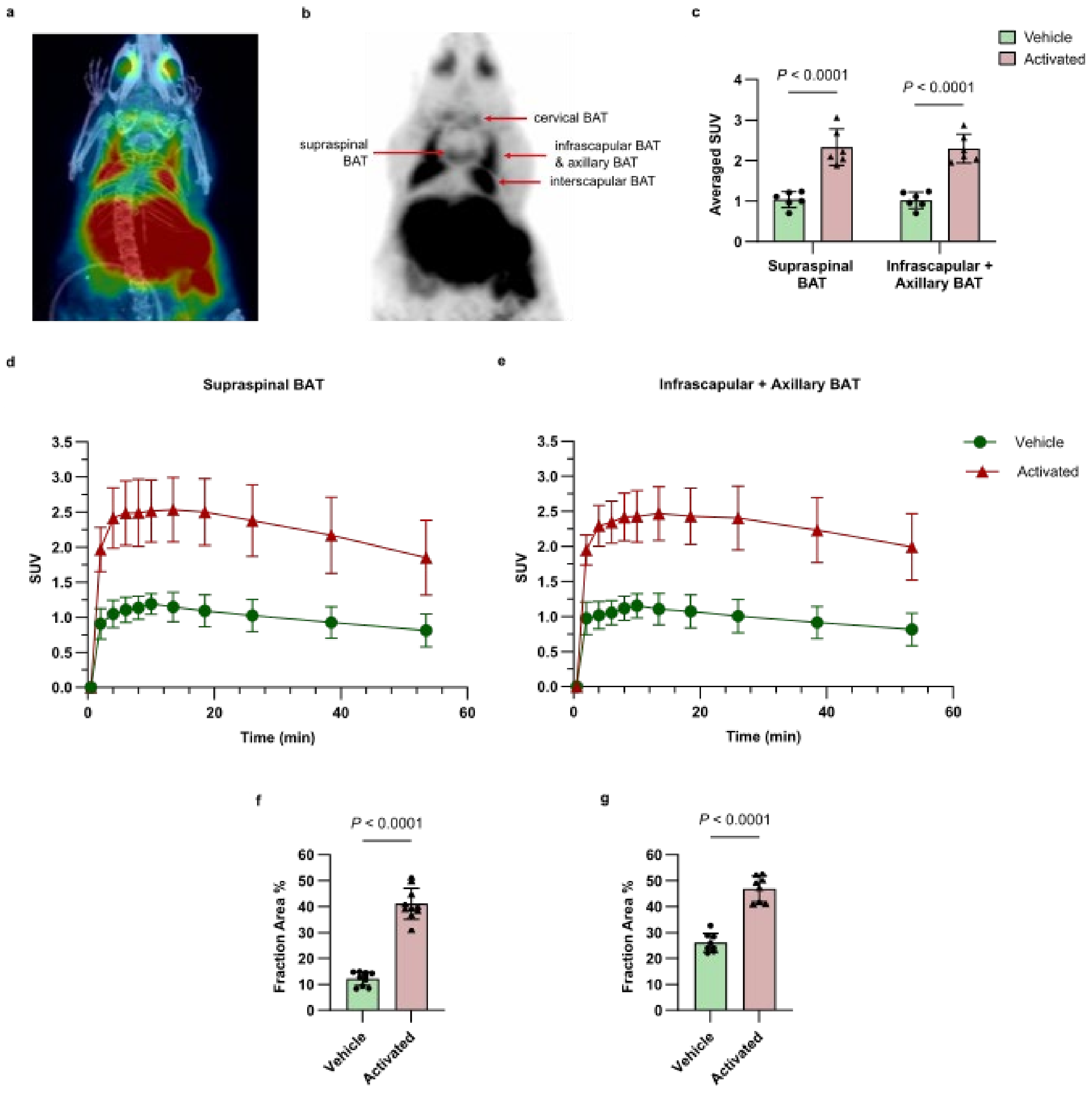

Fig. 5. **a,** Averaged PET image (time frame 0-60 min) of [^{11}C]J147 in CL316,243-treated Balb/c mice, superimposed on CT (grey). **b**, Maximum intensity projection (MIP) with the following BAT depots labelled: supraspinal BAT, cervical BAT, combined infrascapular and axillary BAT, and interscapular BAT. **c,** Averaged SUV of the supraspinal BAT and combined infrascapular and axillary BAT in vehicle-treated or CL316,243-treated Balb/c mice (n = 6). Data are represented as mean ± s.d., one-way ANOVA with Tukey's post-hoc correction for multiple comparison. **d,** Time activity curve of the supraspinal BAT (n= 6 per group). **e**, Time activity curve of the combined infrascapular and axillary BAT (n = 6 per group). **f,g**, Semiquantification of the immunofluorescence images of ATP5A in infrascapular BAT (**f**) and axillary BAT (**g**) depicted in fraction area percentage. Data are represented as mean ± s.d. Two-tailed Student's *t*-test with Welch correction (**f,g**). Same mice as in Figure 4.

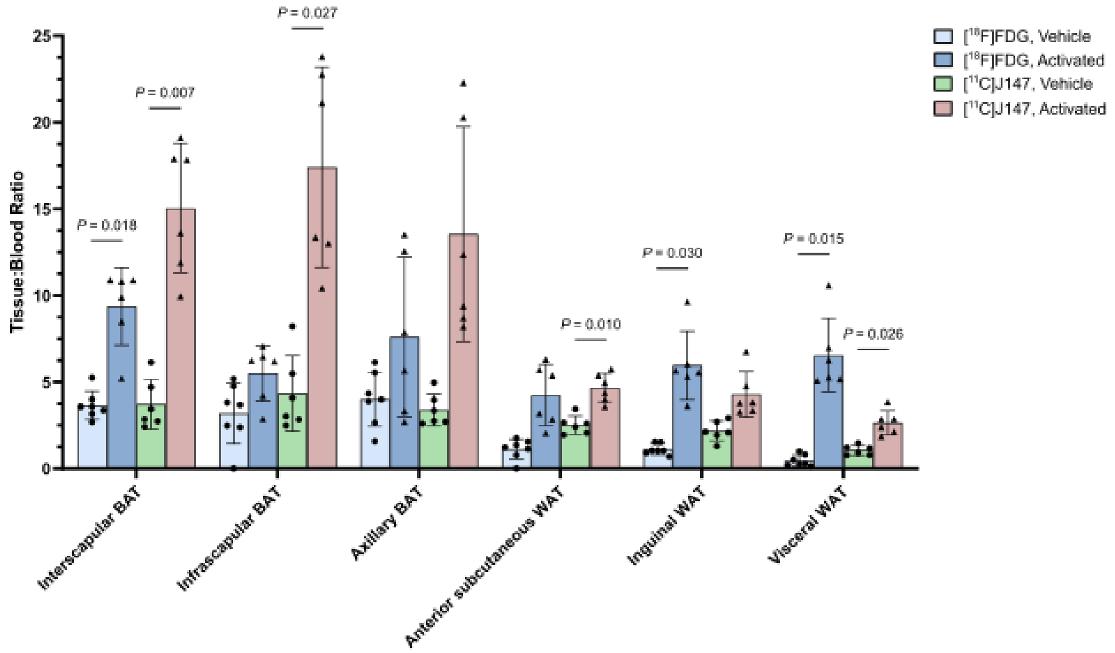

Fig. 6. Biodistribution of [18F]FDG in vehicle-treated (n=7) or CL316,243-treated Balb/c mice (n=6) and biodistribution of [11C]J147 in vehicle-treated (n=6) or CL316,243-treated Balb/c mice (n=6) reported as tissue-to-blood ratio (mean ± SD). Multiple unpaired t-tests with Welch correction and correction for multiple comparisons using Holm-Šídák method.

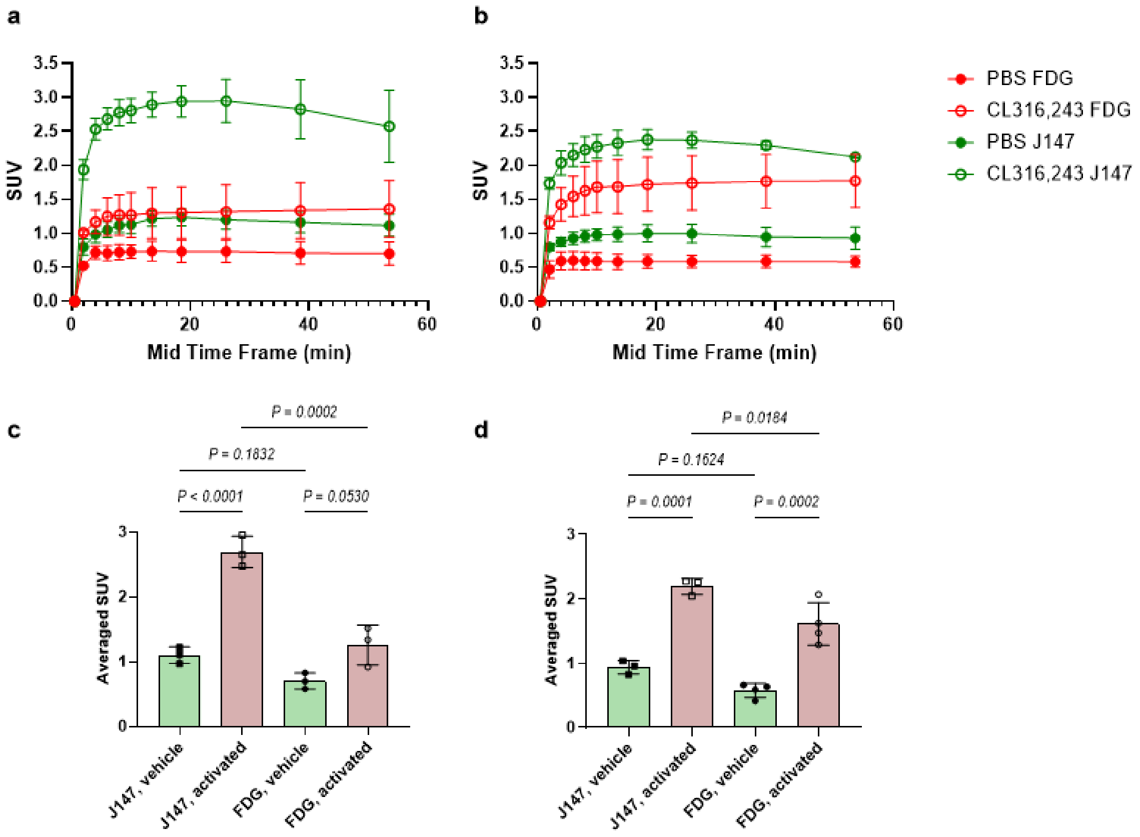

Fig. S8. *In vivo* PET imaging study with [11C]J147 and [18F]FDG in vehicle (or PBS)-treated or CL316,243-treated mice. Time activity curves of [11C]J147 (n=3) and [18F]FDG (n=3) in interscapular BAT in vehicle-treated and CL316,243-treated females (a).

Time activity curves of [11C]J147 (n=3) and [18F]FDG (n=4) in interscapular BAT in vehicle-treated and CL316,243-treated males (b). Averaged SUVs of the vehicle-treated and CL316,243-treated female (c) and male (d) groups. Data are represented as mean ± s.d. One-way ANOVA with Turkey's post-hoc correction for multiple comparisons.

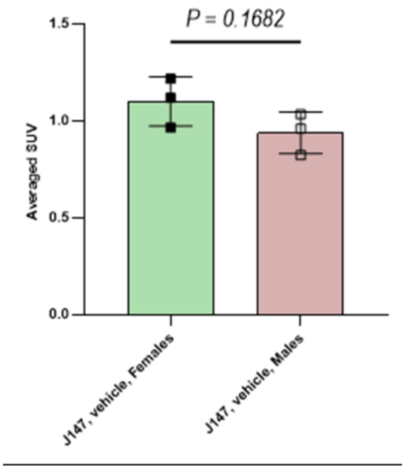

Figure S9. Averaged SUVs of [11C]J147 for the interscapular BAT of the vehicle-treated female and male groups. Data are represented as mean ± s.d. Two-tailed Student's *t*-tests with Welch correction.

10. In line 384, is the radiotracer and blocker co-incubated for 30 minutes, or are they incubated sequentially for 30 minutes each?

The radiotracer and blocker were co-incubated for 30 min. Added "(co-)incubated" in the methods section for clarity.

11. In line 386, the dose of radiotracer should be clearly specified in autoradiography studies.

Added the dose of radiotracer in the methods section. It was already included in the figure legends.

Reviewer #2 (Remarks to the Author):

I co-reviewed this manuscript with one of the reviewers who provided the listed reports. This is part of the Nature Communications initiative to facilitate training in peer review and to provide appropriate recognition for Early Career Researchers who co-review manuscripts.

We thank you for your feedback and your time spent for carefully evaluating our manuscript.

Reviewer #3 (Remarks to the Author):

In this work the authors explore ATP synthase (ATP5A) as a potential imaging target for identifying activated and non-activated adipose tissue. They synthesized [11C]J147, a curcumin derivative, as the novel radiotracer for Positron Emission Tomography (PET) imaging of brown adipose tissue (BAT) and white adipose tissue (WAT). The radiotracer was tested under vehicle or CL316, 243 stimulation of the fat depots. In vivo the radiotracer was also evaluated in mice of young and old age. Uptake of the radiotracer was compared to [18F]FDG, current clinical standard for identification of activated fat, and [18F]BCPP-EF, a mitochondrial complex 1 marker. Efforts to validate imaging findings were immunohistochemistry for ATP5A and UCP1, autoradiography, siRNA knockdown of ATP5A, and biodistribution studies. Given that adipose tissue is a therapeutic target to combat obesity and other metabolic diseases, imaging biomarkers for drug screening are an important need.

The strengths of the study include, well-defined methodology including radiosynthesis and imaging validation attempts, evaluation of the specificity of the radiotracer, comparison to previously evaluated radiotracers, multi-time point imaging, and sufficient methodological detail to reproduce the studies. The major findings of the study are: (1) Higher uptake of [11C]J147 in activated BAT compared to [18F]FDG and [18F]BCPP-EF; (2) ATP synthase expression correlated with interscapular BAT activation and visceral WAT activation, validating it as a potential biomarker; and (3) Compared to [18F]FDG, superior visualization of other BAT fat depots, as identified in reference 11 using a lipid based imaging agent, including supraspinal, axillary, and infrascapular; and acknowledgement of limitations in the discussion.

We appreciate the reviewer's positive feedback on our manuscript and the clear and precise summary of our work.

There are major weaknesses and concerns that should be addressed before this radiotracer can considered for further evaluation as a potential novel imaging tool in humans:
(1) The experimental design lacks mechanistic studies to determine whether [11C]J147 directly binds ATP synthase or if other mitochondrial proteins contribute to the uptake;

We thank reviewer for the valuable comments. We added the following experiments in the revised manuscript. a) An in vitro ATP hydrolysis assay was used to quantify the specific inhibitory effect of J147 on the ATP synthase in isolated mouse liver mitochondria; b) different blockers were included in vitro autoradiography experiments; c) Competition binding studies were performed using J147 and another ATP inhibitor CJ1-34 to investigate the in vivo specificity of [^{11}C]J147. Figures 1e, 1g and figures 3h-i were prepared based on these results. We have added the following sentences to the experiment a). "The

specificity was further investigated in an in vitro functional assay with isolated mitochondria, monitoring ATP hydrolysis[41]. Both J147 and oligomycin A, a full inhibitor of ATP synthase, significantly reduced the mitochondrial ATP synthase activity at 100 µM. The mitochondrial complex I inhibitor rotenone had no effect at the same concentration (P = 0.951) (Fig. 1g)." For details about our results on b) and c), please refer to our response to general question 1 from reviewer 1.

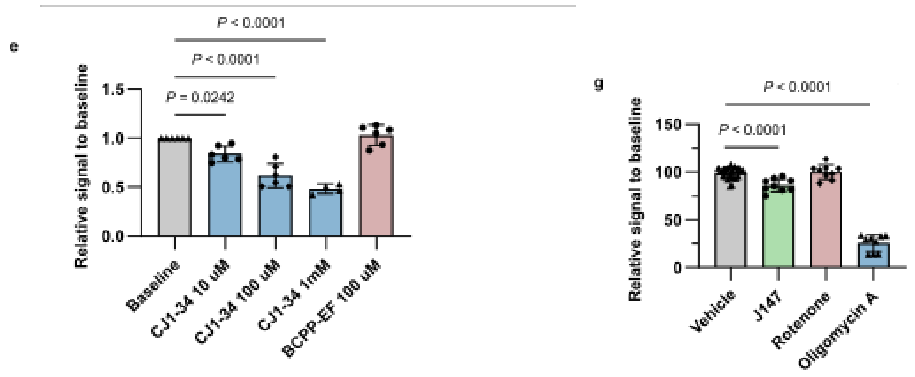

Fig. 1. e, Semiquantification of autoradiographs from interscapular BAT slices of CL316,243-treated mice incubated with [^{11}C]J147 (3–5 nM; baseline condition) and co-incubated with indicated concentrations of CJ1-34 or BCPP-EF (100 µM, served as a negative control) (n = 4-6 for each). **g**, ATP hydrolysis assay showing significant effect by J147 treatment (n=9) and oligomycin A treatment (n=9) but no effect by rotenone (all 100 µM, n=9.

(2) The sample sizes for PET imaging and biodistribution studies (n=3/group) are small which reduces the statistical power and increases the risk of type II errors. Limited ability to generalize findings, especially across BAT depots; The in vitro siRNA knockdown studies also had a small sample size (n=3) for qPCR and radiotracer uptake studies, but the effects sizes were large, reducing impact of variability;

We have increased the sample size for the PET imaging and biodistribution studies to n=6/7 per group to increase the statistical power and reduce risk of type II errors. The new results are presented in Figures 4-6 (see answers to the first reviewer).

(3) While in general the validations studies support the PET imaging there are concerns that should be addressed to strengthen the manuscript and the potential of ATP synthase as an imaging biomarker: (a) No direct enzyme activity assay was performed, correlation coefficients between ATP5A expression and [11C]J147 SUV were not provided, and there was no quantitative western blot validation of ATP5A expression; (b) In vitro autoradiography only measures binding, not enzyme activity, there was no comparison to another ATP synthase inhibitor or competitor to confirm selectivity and no ATP synthase knockout models were used to validate loss of tracer binding; (c) It remains unclear whether the results obtained in PC3 prostate cancer cells would translate to BAT function; (d) No complementary overexpression studies were performed to show increased uptake with higher ATP5A levels and western blot validation of ATP5A knockdown was missing; (e) In the biodistribution studies there is confirmation of PET imaging but lack of direct enzymatic activity validation. The missing enzyme activity assays in the validation methods would have provided direct evidence that ATP synthase function, not just expression, correlates with the PET tracer uptake. This could help distinguish between ATP synthase as an active metabolic regulator versus a passive biomarker and would confirm that [11C]J147 is tracking ATP production, not just ATP synthase protein levels.

We thank reviewer for the comments.

(a) We have added the ATP hydrolysis assay to demonstrate the impact of J147 on ATP synthase activity, and compared it with rotenone and oligomycin, which are specific inhibitors of complex I and ATP synthase, respectively. The data we collected from immunohistochemistry and PET imaging data were consistent with each other. However, correlation coefficients between ATP5A expression and [^{11}C]J147 SUV were not analysed due to the limited data. In the revised manuscript, we have changed the term "correlate" to "in agreement" or "consistent".

(b) We included an additional blocker (compound CJ1-34), which has recently been identifies as an ATP synthase inhibitor, in the autoradiography studies. Following results were included in the revised manuscript: "to corroborate the specificity of [^{11}C]J147 accumulation in activated BAT, we co-incubated slices of intrascapular BAT of CL316,243-treated mice with [^{11}C]J147 (3-5 nM) and our recently developed partial inhibitor of ATP synthase, CJ1-3440, at 10 µM, 100 µM and 1 mM. BCPP-EF, which binds to complex I, was used as a negative control. CJ1-34 competed with [^{11}C]J147 binding in a concentration-dependent manner, while no significant blocking was found for BCPP-EF (P = 0.973) (Fig. 1e). Representative radioautographs are shown in Extended Data Figure 1." We have inquired vendor about ATP synthase knockout models for *in vivo* validation. However, knocking out such an essential protein is lethal for the animals and would make it challenging to perform experiments from an ethical standpoint.

(c) We acknowledge that PC-3 prostate cancer cells are different than brown adipose tissues. However, evidence has shown that the ATP synthase can be ectopically expressed on plasma membrane of tumour cells and adipocytes (Chi SL and Pizzo SV: Cell surface F1Fo ATP synthase: A new paradigm? Ann Med. 38:429–438. 2006; Li W, Li Y, Li G, Zhou Z, Chang X, Xia Y, Dong X, Liu Z, Ren B, Liu W, Liu W, et al: Ectopic expression of the ATP synthase β subunit on the membrane of PC-3M cells supports its potential role in prostate cancer metastasis. Int J Oncol 50: 1312-1320, 2017). Another reason to use this model was the fact that during cell-uptake experiments cells experience multiple washing, and from our experience many cell types detach after this procedure. PC-3 cells instead show extremely good adherence to the bottom of the culture dish.

(d) We appreciate this important question. For quantify ATP5A protein levels, immunofluorescence staining of ATP5A was conducted and the results align well with the in vitro autoradiography results (Fig 1 and 2). In addition, by knocking down ATP5A at the mRNA level (confirmed by qPCR) and observing reduced tracer binding in autoradiography, we directly demonstrate that the tracer signal depends on ATP5A expression. For ATP5A, multiple studies have demonstrated good correlation between mRNA and protein expression levels, as ATP5A is a structural component of ATP synthase and is not subject to significant post-transcriptional regulation under normal conditions. We agree that Western blot would provide a more accurate measurement of protein levels. Therefore, we used Western blot to quantify the ATP5A expression in the adipose tissue of a mouse model of diabetes (see Figure 7 above for reviewer 1). The results pf Western blot analysis was consistent with those obtained using immunofluorescence staining.

Fig. 1.

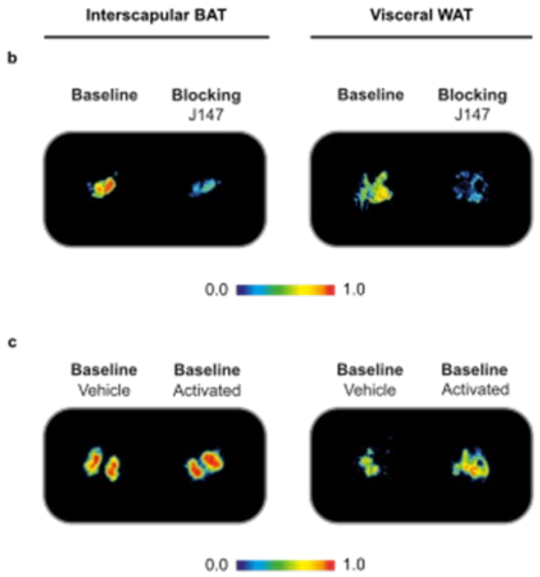

Fig. 2.

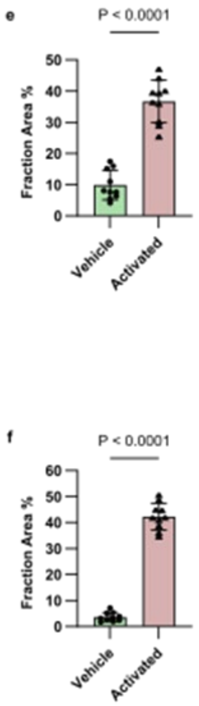

Fig. 1. b, c, *In vitro* autoradiography with [^{11}C]J147 on tissue slices from interscapular BAT and visceral WAT, representative autoradiographs for each group. **b**, Incubation with [^{11}C]J147 (3-5 nM; baseline condition) or [^{11}C]J147 (3-5 nM) together with 100 µM unlabelled J147 for competition (blocking condition). **c,** Mice were treated with vehicle (control) or CL316,243 (activated), and tissue slices were incubated with [^{11}C]J147 (3-5 nM).
Fig. 2. e,f, Semiquantification of the immunofluorescence images of ATP5A in interscapular BAT (**e**, n = 10) and visceral WAT (**f**, n = 10) depicted in fraction area percentage. Data are represented as mean ± s.d. Two-tailed Student's *t*-test with Welch correction (**e,f**)

(e) The primary purpose of the radiotracer [^{11}C]J147 is to quantify available ATP synthase expression levels in vitro and in vivo. Although our PET tracer cannot directly measure enzymatic activity, ATP synthase abundance reflects mitochondrial content and potential ATP-dependent thermogenic activity, including futile cycle–driven heat production in brown and beige adipocytes. It is important to note that the amount of [^{11}C]J147 administered in vivo is extremely low, far below levels that would have a pharmacological effect, ensuring that the injection of the tracer does not interfere with the biological system under study.

(4) The authors primarily used Student's t-test (unpaired, two tailed) to compare tracer uptake between activated and non-activated BAT, Autoradiography and siRNA knockdown studies, and SUV differences between [11C]J147 and [18F]FDG. There was no multiple comparisons correction (acknowledged in Figure 1), no power analysis reported. ANOVA with post-hoc correction would have been more appropriate for multiple comparisons across fat depots;

We have adjusted to ANOVA with post-hoc correction for multiple comparisons or have included post-hoc correction for the biodistribution studies using Welch correction and Holm-Šídák method to correct for multiple comparisons. This correction was already done but incorrectly stated in the manuscript which has now been appropriately corrected.

(5) The impact of anesthesia used in the PET imaging is not optimally considered. The authors report using isoflurane anesthesia for all PET/CT imaging, giving internal control but its impact was not addressed. There is no discussion of the potential suppressive effects of isoflurane on BAT activity. It is known to blunt BAT activation by reducing norepinephrine signaling. Alternatives such as ketamine-xylazine that better preserve BAT function were not considered. While activated and non-activated groups were treated equally this does not rule out the possibility that overall tracer uptake was lower than it would be under awake conditions. This is particularly important given that [18F]FDG uptake is known to be sensitive to anesthesia and in light of the low SUVs reported in some of the figures (~3 or even less than 1 in some older mice). In clinical practice, it is quite possible that such low values would not allow the radiologist to confidently identify these depots above surrounding background tissue.

We thank reviewer for the valuable suggestion. Unfortunately, we do not have the animal licence of using different alternative anaesthesia reagent for PET imaging. Therefore, we have included biodistribution studies under awake conditions. We indeed observed higher [^{18}F]FDG uptake in wake mice compared to the anesthetized mice. However, [^{11}C]J147 demonstrated still comparable results. The results are included in the revised manuscript.

"The potential influence from the anaesthetics used on tracer uptake was investigated by performing the similar biodistribution study in awake male animals. [^{11}C]J147 or [^{18}F]FDG was administrated in conscious mice (n = 3 for each group) which were euthanized under anaesthesia at 15 min p.i. to collect the adipose tissues. The results showed consistently higher uptake of [^{11}C]J147 in all fat depots compared with [^{18}F]FDG, aligning with the biodistribution patterns observed under anaesthesia (Supplementary Figure S15)."

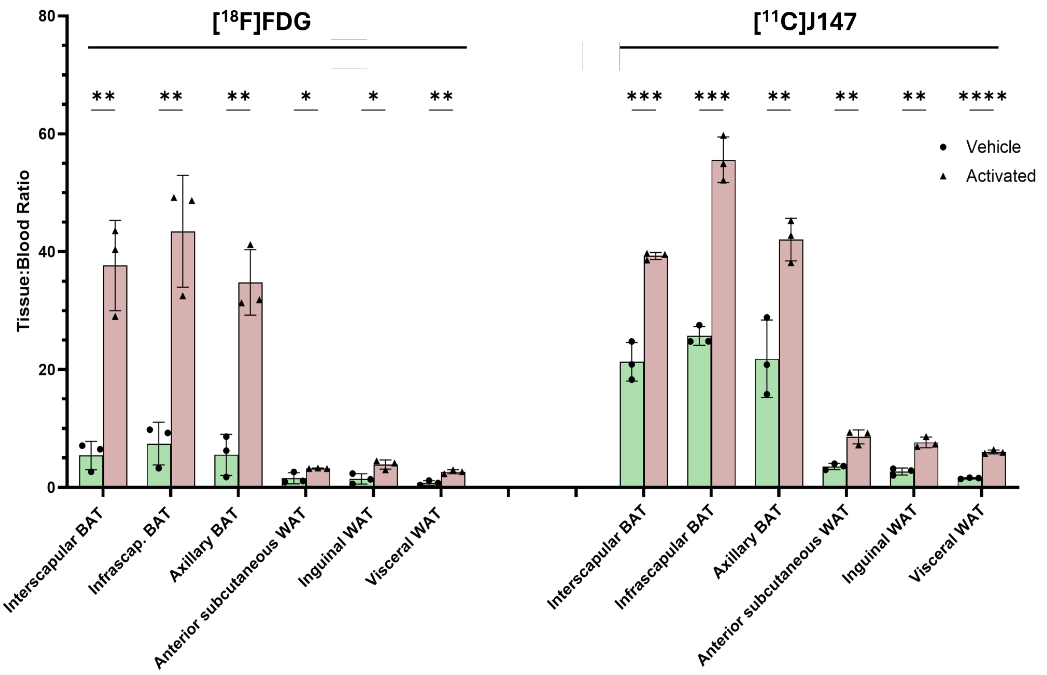

Figure S15. Biodistribution of [^{18}F]FDG in vehicle-treated (n=3) or CL316,243-treated Balb/c mice (n=3) and biodistribution of [^{11}C]J147 in vehicle-treated (n=3) or CL316,243-treated Balb/c mice (n=3) reported as tissue-to-blood ratio (mean ± SD). Animals were injected awake for both tracers and sacrificed at 15 min after iv injection. Multiple unpaired t-tests with Welch correction and correction for multiple comparisons using Holm-Šídák method.

There are other minor issues that should be addressed. Reference 11 is not a mitochondrial potential targeting tracer as stated in the text. It is a CD36 substrate and marker of lipid oxidation but cannot be metabolized. Its uptake is related to the use of this pathway for fuel. There are multiple types of "SUV". The authors do not state which SUV they are reporting, e.g. mean, max, peak etc. The PMOD program is not reconstruction software as the authors imply on line 469. They should state how the images were reconstructed and subsequent analysis was performed using PMOD.

Thank you for pointing out the mistake on reference 11. We have now included it separately as a fatty acid tracer (ref 12). We have revised our Methods part to clarify the related message about "SUV" and "PMOD". Details on how the images were reconstructed and analysed are also provided in the Methods part.

"PET data were reconstructed with 2D ordered-subsets expectation maximisation (2D-OSEM), applying random scatter correction and decay correction but no correction for attenuation and analysed with PMOD software (PMOD Technologies, Zurich, Switzerland; version 4.5) with user-defined time frames with a voxel size of 0.3875 × 0.3875 × 0.755 mm3. PET/CT images were generated with PMOD software. Time activity curves (TACs) were calculated by PMOD with manually defined regions of BAT. The brain regions of interest (ROIs) were defined on an MRI T2 (W. Schiffer) template provided by PMOD. Results are presented as mean standardised uptake value (SUV), indicating the decay-corrected radioactivity per cm3 divided by the injected radioactivity per gram of body weight – assuming a tissue density of 1.0 g/cm^3."

Dear Reviewers,

Thank you for your positive feedback. Please see our point-by-point responses to the requests below.

REVIEWERS' COMMENTS

Reviewer #1 (Remarks to the Author):

The authors have addressed most concerns. In the revised manuscript, the specificity of [11C]J147 has now been comprehensively validated through both in vitro autoradiography and in vivo PET imaging studies. Importantly, the imaging performance of [11C]J147 has been further evaluated in metabolic disease models, including STAM and GLP-1 treatment, which substantially strengthens the translational relevance of the study. Overall, the experimental design is now more comprehensive, and the data is more convincing.

We thank reviewer's clear summary and encouraging statements.

I have only a few minor comments that should be addressed prior to publication:

1. To facilitate a clearer comparison between the radiotracer and its blocking agent, I recommend that the chemical structure of CJ1-34 be included in the Supplementary Materials. This would help readers better understand the structural relationship and differences between the tracer and the blocker.

The chemical structures of CJ1-34 and J147 were added in the Supplementary Figure S9 as required.

2. In the PET imaging studies, after CJ1-34 blocking, tracer uptake appears to continue increasing and approaches baseline levels at later time points. The authors are encouraged to discuss possible explanations for this observation, including incomplete receptor occupancy or reversible binding kinetics, contributions from non-specific uptake, tracer metabolism or radiometabolite accumulation. More discussion of these potential mechanisms would improve the interpretation of the blocking results.

We thank reviewer for the insightful comments. Following sentences are added in the revised manuscript:

Notably, the PET time–activity curve under CJ1-34 blocking conditions (Fig. S10) demonstrated a marked reduction in tracer uptake at early time points, followed by a gradual increase over the course of the PET scan. At later time points, tracer uptake approached the levels observed in the absence of the blocker. This pattern is consistent with competitive tracer binding during the early phase and reflects the lower binding

potency of CJ1-34 relative to J147, resulting in reduced and less sustained target occupancy.

Reviewer #2 (Remarks to the Author):

I co-reviewed this manuscript with one of the reviewers who provided the listed reports. This is part of the Nature Communications initiative to facilitate training in peer review and to provide appropriate recognition for Early Career Researchers who co-review manuscripts.

We thank you for your time spent for carefully evaluating our manuscript.

Reviewer #3 (Remarks to the Author):

Thank the authors for making serious attempts to address the limitations posed in the review. My concerns have been adequately addressed within a reasonable way. The rather limited blocking of the [11C]J174 remains disappointing but the authors offer a plausible explanation, though other possibilities exist such as affinity mismatch between the blockers and their [11C]J147, some degree of non-specific uptake of [11C]J147, or levels of occupancy on the ATPase by endogenous ligands.

We thank reviewer for the insightful comments. Following sentences are added in the revised manuscript:

The relatively modest effects observed may be attributed to incomplete receptor occupancy, partial non-specific uptake of [^{11}C]J147, and the high abundance of ATP synthase, which may require higher doses and continuous infusion, rather than a single bolus injection, to achieve adequate target engagement.
